# “Gender dimensions of quality of life”: The determinants of life satisfaction among South Africans residing in the Gauteng province—Using a multilevel psychodemographic analysis of the GCRO’s quality of life survey (2009–2024)

**DOI:** 10.1371/journal.pone.0332626

**Published:** 2025-09-15

**Authors:** Monica Ewomazino Akokuwebe, Salmon Likoko, Shamsunisaa Miles-Timotheus, Moyahabo Mabala

**Affiliations:** 1 SAMRC Developmental Pathways for Health Research Unit, Department of Paediatrics, School of Clinical Medicine, Faculty of Health Sciences, University of the Witwatersrand, Johannesburg, South Africa; 2 Demography and Population Studies, School of Social Sciences, University of Witwatersrand, Johannesburg, South Africa; 3 Gauteng City-Region Observatory (GCRO), University of the Witwatersrand, University of Johannesburg, Johannesburg, South Africa; 4 Group Finance, Johannesburg, South Africa; University of Fort Hare, SOUTH AFRICA

## Abstract

This study examined gender-related differences in life satisfaction among 70,314 South Africans (33,325 males and 36,989 females) using data from the Gauteng City-Region Observatory (2009–2024). Life satisfaction levels (suffering, struggling, thriving) were analyzed in relation to demographic factors including age, education, employment, income, and population group. Using multilevel psychodemographic analyses, the study uniquely identifies how these factors operate differently for males and females over a 15-year period. Education, employment, and income were significant predictors of life satisfaction (all p < 0.01); for males, income had stronger indirect effects, while for females, education (β = 0.40, p < 0.01) and income (β = 0.93, p < 0.001) exerted direct effects. These findings reveal gender-specific pathways to life satisfaction, highlighting the importance of considering both individual and structural determinants. The results have policy relevance by underscoring the need for targeted, gender-sensitive interventions in education, employment, and income support to enhance well-being and promote equity among South Africans in Gauteng province.

## 1. Introduction

Understanding Quality of Life (QoL) is central to evaluating subjective well-being, with life satisfaction recognized as a key socio-economic indicator [1–4]. Psychodemographic research highlights how demographic characteristics, gender identity, language, socialization, racial group membership, and cultural individuality influence subjective well-being through the interplay of external conditions and internal psychological processes [3–7]. Life satisfaction thus reflects how individuals compare their current circumstances to their ideal life, considering both material and spiritual aspects [8,9]. In South Africa, integrating life satisfaction measures into national policy frameworks could improve monitoring of demographic indicators such as education, employment, income, and access to resources [10,11]. South Africa’s multicultural society presents a complex landscape for studying life satisfaction, where social status and persistent inequalities strongly influence well-being [12–17]. Despite social progress, the country remains one of the most unequal globally, with a predicted Gini coefficient of 0.63 in 2024 [11]. Previous studies have shown that while improved living standards enhanced quality of life for Black South Africans, disparities persisted compared to other groups [7]. Economic challenges, unemployment, political changes, and the COVID-19 pandemic have further shaped public trust, governance, and life satisfaction [4,14].

Life satisfaction reflects a multidimensional comparative process that spans happiness, economic stability, social relationships, and well-being [8,12]. Existing research in South Africa has reported mixed findings on the effects of economic and demographic determinants [18,19]. Although numerous theoretical and empirical studies exist, none provide universal explanations [20–23], and gender-based differences remain underexplored. Psychodemographic influences such as education, employment, and income often vary by gender, complicating the task of distinguishing direct effects from social and cultural selection processes [1,2]. Globally, determinants of life satisfaction include age, gender, race, marital status, health, education, and income [14,24]. Similar associations have been reported across sub-Saharan Africa, including Ghana, Tanzania, Ethiopia, and Malawi [1,16]. In South Africa, factors such as religion, migration, social investment, job security, poverty, and class status have also been found to influence well-being [23–27]. Recent South African studies have investigated social networks, migration, demographics, and racial differences [4,8], but methodological limitations remain. Determinants such as chronic illness, poor healthcare, unemployment, and inequality continue to shape life satisfaction outcomes [2,27].

The Gauteng City-Region Observatory (GCRO) Quality of Life Survey (2009–2024) [28–34] provides a unique opportunity to address these gaps. This study uniquely examines life satisfaction in Gauteng using a multilevel psychodemographic approach to a large, longitudinal dataset over a 15-year period (2009–2024), integrating individual, household, and community-level factors while explicitly considering gender differences. Unlike previous research, it combines long-term survey data with multilevel modeling to reveal how socio-economic, cultural, and environmental determinants intersect to shape life satisfaction across sexes. Importantly, it emphasizes gender differences in life satisfaction—a dimension rarely examined systematically in South Africa. Several studies have shown that education, work, and societal factors are more strongly associated with male life satisfaction, while female life satisfaction is linked to marital status and the quality of social relationships [12,18]. It is therefore expected that our findings will reflect these gendered differences, addressing a gap in existing research. This study examines life satisfaction by sex distribution among South Africans, with specific aims to: (1) measure life satisfaction across sex and age groups; (2) assess bivariate associations between life satisfaction and demographic determinants by sex; and (3) propose a mediation model linking demographic determinants to life satisfaction by sex across survey years.

## 2. Methods

### 2.1. Study area

This study was conducted in Gauteng Province, South Africa’s smallest and most urbanized province [35]. South Africa, officially the Republic of South Africa (RSA), has a population of nearly 60 million and borders six countries while surrounding Lesotho. It operates a tripartite capital system: Pretoria (executive), Bloemfontein (judicial), and Cape Town (legislative) [35]. The country is ethnically diverse, with Black South Africans making up over 81% of the population, and Christianity as the predominant religion [35,36]. Despite being an upper-middle-income economy and a member of G20, BRICS, and the Commonwealth, South Africa faces high levels of poverty, unemployment, and inequality, with 25% of its population living on less than US$1.25 per day as of 2008 [36]. A notably youthful demographic, with 37% aged 14–35, characterizes its population profile [36,37]. Gauteng is South Africa’s most populous and urbanized province, with 15 million residents in 2022, accounting for 26.6% of the national population [38,39]. It contributes about a third of the country’s GDP, producing R1.59 trillion in 2016 and attracting over a third of internal migrants, while provinces like Limpopo and Eastern Cape experienced outflows [35,37,38]. By 2022, 89.7% of households had access to flush or chemical toilets, up from 86.5% in 2011, reflecting infrastructure progress [40–42]. The province’s cosmopolitan character is evident in its diverse languages, with isiZulu (25%), Sesotho (13.4%), Sepedi (12%), English (10.7%), and Setswana (9.8%) widely spoken, alongside all eleven official languages [33,35,37,40].

### 2.2. Study design and data source

This study draws on the Gauteng City-Region Observatory (GCRO) Quality of Life (QoL) survey data collected between 2009 and 2024. The GCRO is a partnership between the University of Johannesburg, the University of the Witwatersrand, the Gauteng Provincial Government, and the South African Local Government Association [28]. The QoL survey is a large cross-sectional household survey targeting adults (18+) in Gauteng, designed to capture socioeconomic conditions, perceptions of public services, psychological outlooks, values, and overall quality of life [28–34]. Data support provincial monitoring, Sustainable Development Goals (SDGs) tracking, and policy development [43–45]. The survey employed a multi-stage, stratified cluster sampling approach. Since its inception in 2009, GCRO has completed seven rounds: Round 1 (2009) [28], Round 2 (2011) [29], Round 3 (2013–2014) [30], Round 4 (2015–2016) [31], Round 5 (2017–2018) [32], Round 6 (2020–2021) [33], and Round 7 (2023–2024) [34]. Across these rounds, 86,832 enumeration areas were sampled, with 70,314 individuals forming the final analytic dataset (male = 33,325; female = 36,989) [28–34]. Within municipalities, sampling was stratified by urban–rural distribution and household counts, ensuring representativeness.

The authors accessed anonymized secondary data via the GCRO website on 7 August 2024. No personally identifiable information was available during collection or analysis, as datasets were provided under GCRO’s data-sharing protocols.

### 2.3. Study population and sample size

The study population comprised South African nationals stratified by sex who resided in the Gauteng province of South Africa. South African nationals refers to individuals who hold South African citizenship. By conceptual definition and operationalization in this study, a South African national is any person born to at least one South African parent who receives citizenship at birth; children born to a legal resident of the country are permitted to obtain South African citizenship only when they reach the age of majority. Also, foreign nationals may be granted citizenship after meeting a residence requirement (usually five years). This definition was used in line with the South African nationality law detailing the conditions by which a person is a national of South Africa. The primary law governing such nationality requirements is the South African Citizenship Act 1995, which came into force on the 6th of October 1995 [46]. The total number of South African nationals from the 2009–2024 GCRO datasets was 70,314 comprising 33,325 males and 36,990 females for the sample size for this study, after the exclusion of missing variables in the datasets cleaning. The sample size of 70,314 included the age range from 18 to 48 + years [28–34], and this comes after the datasets from different survey years were combined. The data from each year was combined to create a comprehensive dataset, enabling a broader perspective on trends and changes over time. The datasets from 2009–2024 were added together to expand the range of generalization in order to improve the statistical power of analysis for reliable conclusions, allowing comparative analysis for potential causal relationships and control for confounding variables, hence justifying its inclusion [47–49]. In addition, an iteration variable was created to be able to identify changes over time.

### 2.4. Operational definition of concepts

Sex and gender are two different concepts, but they are often used interchangeably [50,51]. Sex refers to biological differences (such as chromosomes, hormones, and reproductive organs) [50] while gender refers to socially constructed roles, behaviours, activities, and expectations associated with femininity and masculinity [51]. Therefore, in this study, we treated sex and gender as distinct constructs. Sex was used to stratify the population into male and female categories, while gender was utilized to explore roles, identities, and societal expectations related to life satisfaction.

### 2.5. Measures

The GCRO Quality of Life Rounds 1–7 (2009‒2024) [28–34] survey—Full Questionnaires were included in the field data collection instrument, which comprised questions focused on: (1) demographic details of the enumerated population (population group, sex, age, language), (2) housing (dwelling type, tenure, satisfaction with dwelling, perceived quality of housing and housing allocation), (3) household services (water, sanitation, refuse removal, energy sources), (4) migration and health (including disability), (5) education and employment (including employment sector), (6) community services (amenities, transport, leisure activities, safety and crime), (7) financial data (including debts, income, and social grants), (8) household assets (Telephone, Television, Computer, Radio, Music system, Satellite TV [e.g., MNET, DSTV], Internet connection, Car, Bicycle, Fridge), (9) public participation and governance, (10) perceived personal well-being, and 11) quality of life of respondents. We used data from questionnaires administered to randomly selected women and men living in the surveyed households [28–34].

### 2.6. Outcome variable

The outcome variable, overall life satisfaction, was measured using the Satisfaction with Life Scale (SWLS), a versatile tool that can be adapted to different cultural contexts. Initially developed by Diener et al. [52], this five‐item instrument asks respondents to rate aspects of their lifestyle on a scale from 1 (“very dissatisfied”) to 5 (“very satisfied”), with total scores calculated as recommended by Diener et al. [53]. Building on this, Deaton [54] proposed a further evaluation by integrating Cantril’s Self-Anchoring Ladder of Life Satisfaction [55] alongside Gallup’s measure [56]; in this approach, respondents indicate their life satisfaction on a scale ranging from 0 to 10. This modification was designed to provide a more nuanced exploration of life satisfaction determinants while accounting for various psychodemographic factors. Thus, during the survey, participants reported their satisfaction level and position on the ladder. Following the adoption of Cantril’s and Gallup’s scales, the SWLS scores were reclassified into three categories: “Thriving” for scores 7–10 (coded as 2), “Struggling” for scores 5–6 (coded as 1), and “Suffering” for scores 0–4 (coded as 0). This recording enhances interpretability and offers a clearer picture of overall well-being in the South African context. Prior studies have validated the SWLS with Cantril’s Ladder and Gallup’s approach as reliable and robust tools for assessing life satisfaction in various settings [56–60].

#### 2.6.1. Operationalization of life satisfaction levels.

The operational definitions of the levels of life satisfaction (suffering, struggling, and thriving) provide a clear understanding of the respondents’ varying levels of life satisfaction and well-being, using Gallup [54] and Cantril’s Self-Anchoring Ladder of Life Satisfaction measures [55]. Thus,

- **Suffering (0–4)**: Respondents in this category face significant challenges, including health, social, or economic issues. They often struggle to achieve satisfaction in their life, or maintain a stable quality of life, leading to poor overall well-being [52–55].
- **Struggling (5–6)**: Respondents in this category are moderately satisfied with their lives. Despite occasional health challenges, they manage life’s obstacles, alternating between periods of stability and hardship [52–55].
- **Thriving (7–10)**: Respondents in this category experience high life satisfaction, well-being, and optimism. Access to resources and support networks enhances their overall happiness and contentment [52–55].

### 2.7. Explanatory variables

The explanatory variables in the study were grouped into three distinct categories: individual, household, and community-level factors. Variables at the individual level included age, gender, educational attainment, demographic classification, income, and employment status; the number of persons living in the home, the number of individuals under the age of 18, child hunger, the SASSA social grant, the health facility, and medical aid were factors at the household level. Dwelling type, media accessibility, municipalities, and survey years are examples of community-level characteristics [28–34]. The availability of the variables and earlier research findings served as the basis for selecting these predictors [1,21]. Certain variables from the dataset retained their original categorization during this analysis, while others were meticulously redefined and recorded to enhance the study’s precision [28–34]. Most selected variables were evaluated as straightforward binary measures, requiring “yes” or “no” responses. Additionally, some variables were derived by aggregating answers from multiple questions. Comprehensive descriptions of these variables are provided in another section [28–34] (Refer to Table 1 below).

**Table 1:** Detailed explanations of individual-level, household-level and community-level variables categorization.

Individual-level factors	Categorization
Age	1 = 18–27; 2 = 28–37; 3 = 38–47; 4 = 48 + years
Sex	1 = male; 2 = female
Highest education	1 = no education; 2 = primary; 3 = secondary; 4 = higher
Population group	1 = Black African; 2 = Coloured; 3 = White; 4. Indian/Asian
Respondent’s income	1 = no income; 2 = low (R1–R12,800); 3 = middle (R12,801–R25,600); 4 = high (R25,601+)
Employment	1 = not working; 2 = working
Medical aid	1 = no; 2 = yes
Healthcare facility	1 = public; 2 = private; 3 = both public and private; 4 = traditional
Life satisfaction	0 = suffering; 1 = struggling; 2 = thriving
**Household-level factors**	
Number of people living in H/H	1 = 1; 2 = 2; 3 = 3; 4 = 4 + persons
Number of people under 18 in H/H	1 = 0; 2 = 1; 3 = 2; 4 = 3; 5 = 4 + persons
Not enough to feed children	1 = No; 2 = Yes; 3 = No children in H/H
Receiving SASSA social grant	1 = No; 2 = Yes
**Community-level factor**	
Type of dwelling	1 = Formal; 2 = Informal
Basic services	1 = No; 2 = Yes
Municipality	1 = City of Ekurhuleni; 2 = City of Johannesburg; 3 = City of Tshwane; 4 = Emfuleni; 5 = Lesedi; 6 = Midvaal; 7 = Merafong; 8 = Mogale City; 9 = Rand West
Survey years	1 = 2009; 2 = 2011; 3 = 2013–2014; 4 = 2015–2016; 5 = 2017–2018; 6 = 2020–2021; 7 = 2023–2024

Source: Authors’ compilation, 2024.

### 2.8. How questions derived from the 2009–2024 GCRO QoL questionnaire instrument will be analyzed

Questions were retrieved from the Quality of Life I (2009–2020) and Quality of Life 7 (2023/2024) Survey: Full Questionnaire. The questions from the GCRO QoL instrument will be analyzed as presented in Table 2.

**Table 2:** Distribution of variables showing the instrument variable names and how they were analyzed.

S/N	Variable Description	Variable Name	Variable Analysis
1	What is the sex of the respondent?	a2_sex	Categorization of variables and the use of descriptive statistics for analysis
2	How old are you?	q14_2_age	Categorization of variables and the use of summary and descriptive statistics for analysis
3	To which population group does the respondent belong?	a1_pop_group	Categorization of variables and the use of summary and descriptive statistics for analysis
4	What is the highest level of education you have completed?	q14_1_education	Categorization of variables and the use of summary and descriptive statistics for analysis
5	What is the total amount of money brought into the household per month by all household members? Please include all the money coming into the household, from all sources. This includes grants, help from friends and family, and rental or business income. Please exclude amounts deducted for tax, medical aid and pension contributions.	q15_3_income	Categorization of variables and the use of summary and descriptive statistics for analysis
6	In the past 7 days, did you do any type of work, business, or activity for which you got paid or expected to be paid (even if just for one hour)? This includes selling things, piece work, and work that’s part of your own business.	q10_2_working	Categorisation of variables and the use of summary and descriptive statistics for analysis
7	Are you personally covered by any form of medical aid or other medical insurance?	q13_5_medical_aid	Categorisation of variables and the use of summary and descriptive statistics for analysis
8	Where do you usually go for health care?	q13_1_healthcare	Categorisation of variables and the use of summary and descriptive statistics for analysis
9	How many people, including you, live in this household? Please include babies and children.	q14_5_people	Categorisation of variables and the use of summary and descriptive statistics for analysis
10	How many members of this household are under 18 years old?	q14_6_under18	Categorisation of variables and the use of summary and descriptive statistics for analysis
11	In the past 12 months, has there ever been a time when there was not enough money to feed the children in the household?	q6_5_feed_children	Categorisation of variables and the use of summary and descriptive statistics for analysis
12	Does anybody in this household receive a social grant of any type, such as an old age pension, child care or disability grant?	q14_10_social_grant	Categorisation of variables and the use of summary and descriptive statistics for analysis
13	Which type of dwelling does this household occupy?	a3_dwelling_type	Categorisation of variables and the use of summary and descriptive statistics for analysis
14	Does this household have any of the following that are in good working order, that is not broken?	q6_3_1_landline; q6_3_2_cell; q6_3_3_tv; q6_3_4_computer; q6_3_5_radio; q6_3_6_dstv; q6_3_7_internet;	Categorisation of variables and the use of summary and descriptive statistics for analysis
15	Region/Municipality Name	municipality_coded	Categorisation of variables and the use of summary and descriptive statistics for analysis
16	How satisfied are you with your life AS A WHOLE these days?	q9_9_life	Bivariate and multivariate regression analysis will be employed
17	In which province or country were you born?	q3_1_birth_prov	Will be used to filter for South African nationals

**Source:** Authors’ compilation, 2024.

### 2.9. Data preparation and analysis

**Fig 1:**
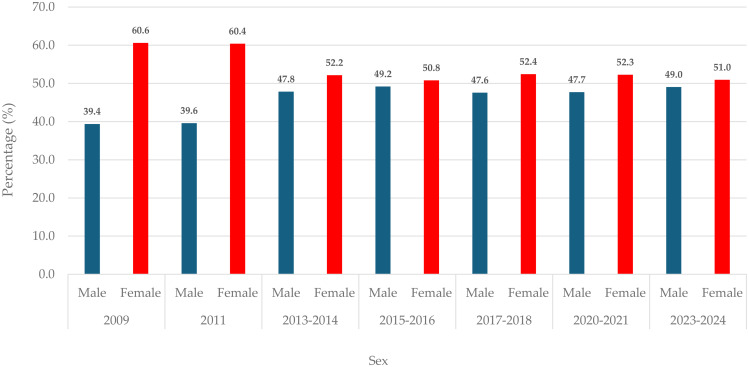
Sex distribution of respondents. The vertical bar chart depicts the percentage distribution of male and female respondents residing in Gauteng, South Africa. The chart illustrates the percentage distribution of observations for each data point or the grouping of the data points of sex by survey years (2009–2024).

**Fig 2:**
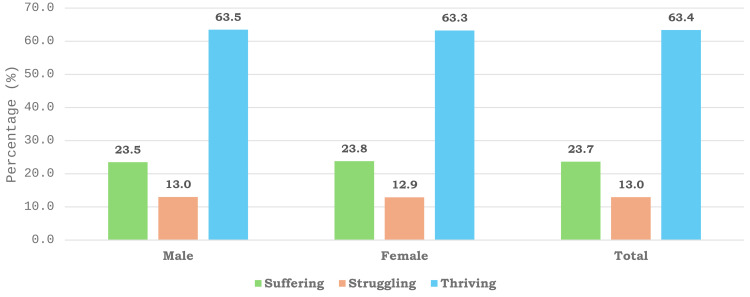
Distribution of life satisfaction levels—suffering, struggling, and thriving—by sex. The grouped bar chart shows that the majority of both male and female respondents reported thriving life satisfaction in Gauteng Province, South Africa. The percentages were calculated from a representative sample, with life satisfaction levels stratified by sex (male and female).

**Fig 3:**
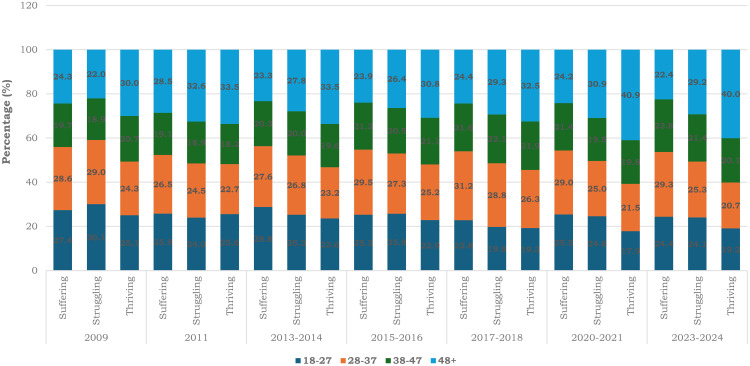
Shows the life satisfaction levels by age as reported by the respondents who are South Africans, residing in Gauteng province, South Africa. The percentage was determined from the representative sample of the number of life satisfaction scales distributed by the different ages across the survey years (2009–2024).

**Fig 4:**
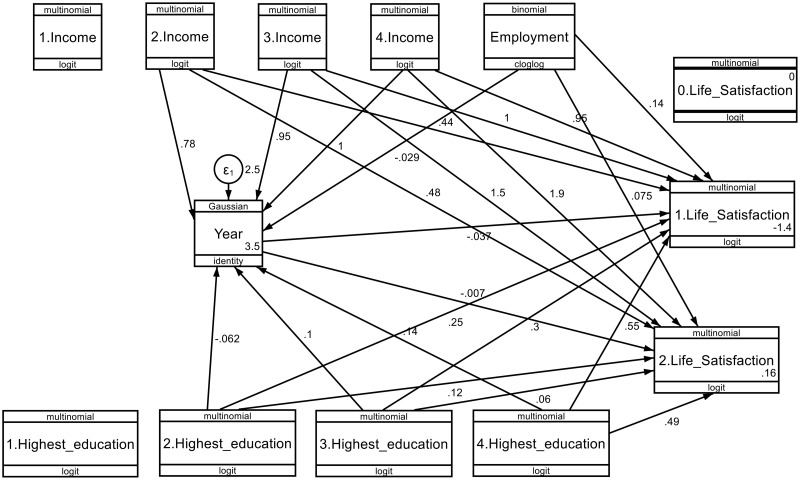
Structural equation model for demographic factors (education, employment, and income), and life satisfaction (struggling and thriving) by sex (males) across the survey years. * p < 0.005; ** p ≤ 0.001.

**Fig 5:**
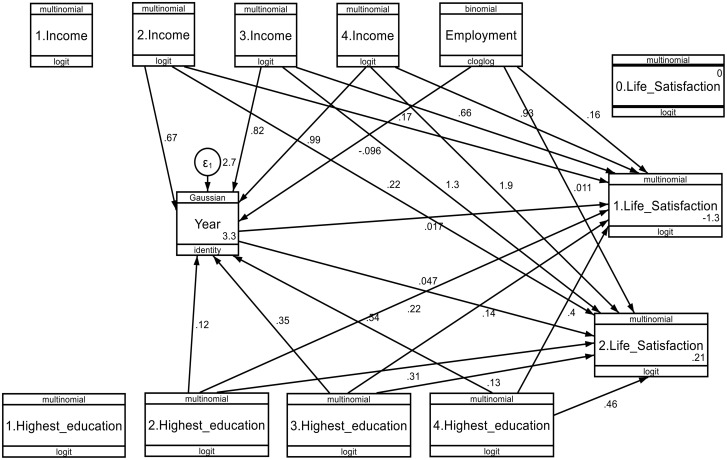
Structural equation model for demographic factors (education, employment, and income), and life satisfaction (struggling and thriving) by sex (female) across the survey years. * p < 0.05; ** p ≤ 0.01.

STATA version 2021 statistical software was used for data management and analysis. The study examined the individual-level, household-level and community-level factors determining life satisfaction among South African nationals aged 18 years or above, residing in the Gauteng Province. The dataset was analyzed according to the study’s objectives and specific measures. Weighting variables from each survey were utilized alongside the “svy command” to address sampling imbalances and account for over- and under-representation. This approach also accommodated the intricate survey design and enhanced the generalizability of the results. The findings derived from the 2009–2024 datasets were presented as follows: First, univariate analysis was carried out to examine the distribution and characteristics using the descriptive statistics to report the demographic characteristics of the respondents, percentage and frequency distributions, and visualizations (vertical bar chart, stacked bar chart, grouped/clustered bar chart) (specific objective 1) (Figs 1–3, and Table 3). Second, bivariate analysis using Chi-square test of associations was employed to test the associations between life satisfaction and demographic determinants stratified by sex using 5% level of significance threshold (specific objective 2) (Table 4). Third, pathway analysis (or pathway model), mediation analysis and generalized structural equation modelling (gSEM) were employed to analyze the specific objective 3. Mediation analysis was utilized to explain the effects of the explanatory variable on the outcome variable (Table 5), while the pathway model was used to identify the direct, and indirect effects of explanatory variables on the outcome variable (Figs 4 and 5), and the generalized structural equation modelling (gSEM) was used to study the effects of the determinants (education, employment, income, and survey years) on life satisfaction as the outcome variable; the variables were either ordinal or binary in nature (Tables 6 and 7) as well as the percentages of the mediation analysis (Table 8). Therefore, combining the pathway models with gSEM will create more robust and comprehensive models to study the direct and indirect effects influencing life satisfaction [61–63]. Further, nonlinear combinations of parameter estimates (nlcom) were generated using the nlcom command on STATA to generate direct and total effects between the outcome, mediator, and independent variables [64,65], and the gSEM and nlcom were both stratified by sex, with statistical significance of ρ < 0.05 signifying level of precision (specific objective 4).

**Table 3:** Socio-demographic Characteristics of Respondents stratified by Sex (n = 70,314).

Demographics	Male (n = 33,325)		Female (n = 36,989)		Total (N = 70,314)	
**Individual-level factors**						
**Life satisfaction**	**Frequency**	**%**	**Frequency**	**%**	**Frequency**	**%**
Suffering	7826	23.5	8813	23.8	16639	23.7
Struggling	4342	13.0	4779	12.9	9121	13.0
Thriving	21157	63.5	23397	63.3	44554	63.4
**Age group**						
18-27	7806	23.4	8046	21.8	15852	22.5
28-37	8742	26.2	9422	25.5	18164	25.8
38-47	7022	21.1	7453	20.1	14475	20.6
48+	9755	29.3	12068	32.6	21824	31.0
**Highest education**						
No education	557	1.7	880	2.4	1437	2.0
Primary	3223	9.7	4377	11.8	7600	10.8
Secondary	21826	65.5	24444	66.1	46270	65.8
Higher	7719	23.2	7288	19.7	15007	21.3
**Pop group**						
Black African	27670	83.0	30692	83.0	58362	83.0
Coloured	1033	3.1	1256	3.4	2289	3.3
Indian/Asian	762	2.3	820	2.2	1581	2.2
White	3860	11.6	4221	11.4	8082	11.5
**Income**						
No income	2203	6.6	1834	5.0	4037	5.7
Low	24762	74.3	29448	79.6	54210	77.1
Middle	3456	10.4	3257	8.8	6713	9.5
High	2904	8.7	2450	6.6	5354	7.6
**Employment**						
Not employed	18893	56.7	26044	70.4	44937	63.9
Employed	14432	43.3	10945	29.6	25377	36.1
**Household-level factors**						
**HH members**						
1	8111	24.3	3557	9.6	11668	16.6
2	6308	18.9	6542	17.7	12850	18.3
3	5357	16.1	7078	19.1	12435	17.7
4 and above	13549	40.7	19812	53.6	33361	47.4
**Children under 18 in HH**						
None	17802	53.4	11846	32.0	29648	42.2
1	5746	17.2	8094	21.9	13840	19.7
2	5256	15.8	8466	22.9	13722	19.5
3	3224	9.7	5525	14.9	8749	12.4
4+	1297	3.9	3058	8.3	4355	6.2
**Child hunger**						
No	19279	57.9	24312	65.7	43591	62.0
Yes	3811	11.4	6506	17.6	10317	14.7
No children in HH	10235	30.7	6171	16.7	16406	23.3
**Social Grant**						
No	20187	60.6	15257	41.2	35444	50.4
Yes	13138	39.4	21732	58.8	34870	49.6
**Health facility**						
Public	11429	34.3	11343	30.7	22772	32.4
Private	18236	54.7	22062	59.6	40298	57.3
Both public and private	3216	9.6	3369	9.1	6585	9.4
Traditional	444	1.3	215	0.6	659	0.9
**Medical aid**						
No	27839	83.5	31683	85.7	59522	84.7
Yes	5486	16.5	5306	14.3	10792	15.3
**Community-level factors**						
**Dwelling type**						
Formal	27931	83.8	31510	85.2	59441	84.5
Informal	5394	16.2	5479	14.8	10873	15.5
**Access to media**						
No	426	1.3	380	1.0	806	1.1
Yes	32899	98.7	36609	99.0	69508	98.9
**Municipality**						
City of Ekurhuleni	8927	26.8	9692	26.2	18619	26.5
City of Johannesburg	11727	35.2	12870	34.8	24597	35.0
City of Tshwane	8024	24.1	8956	24.2	16980	24.1
Emfuleni	1958	5.9	2344	6.3	4302	6.1
Lesedi	271	0.8	324	0.9	595	0.8
Midvaal	264	0.8	245	0.7	509	0.7
Merafong	502	1.5	619	1.7	1121	1.6
Mogale City	990	3.0	1163	3.1	2153	3.1
Rand West	662	2.0	776	2.1	1438	2.0
**Survey year**						
2009	1888	5.7	2907	7.9	4795	6.8
2011	906	2.7	1381	3.7	2287	3.3
2013-2014	8044	24.1	8769	23.7	16813	23.9
2015-2016	8136	24.4	8401	22.7	16537	23.5
2017-2018	6211	18.6	6846	18.5	13057	18.6
2020-2021	4036	12.1	4421	12.0	8457	12.0
2023-2024	4104	12.3	4264	11.5	8368	11.9
**Total**	33325	100	36989	100	70314	100

**Table 4:** Chi-square analysis showing the associations between life satisfaction and factor-related indicators (individual, household, and community) among male and female respondents (n = 70,314).

	Males								Females							
Individual-level factors	Suffering		Struggling		Thriving		X^2^	p-value	Suffering		Struggling		Thriving		X^2^	p-value
Age group	Frequency	%	Frequency	%	Frequency	%			Frequency	%	Frequency	%	Frequency	%		
18-27	2,020	25.9	1,096	14.0	4,690	60.1	248.519	0.000	2,170	27.0	1,098	13.6	4,777	59.4	412.362	0.000
28-37	2,364	27.0	1,228	14.0	5,150	58.9			2,578	27.4	1,282	13.6	5,561	59.0		
38-47	1,651	23.5	945	13.5	4,427	63.0			1,862	25.0	945	12.7	4,646	62.3		
48+	1,791	18.4	1,073	11.0	6,891	70.6			2,203	18.3	1,454	12.0	8,412	69.7		
**Highest education**																
No education	166	29.9	62	11.1	329	59.1	637.891	0.000	270	30.6	113	12.8	498	56.5	445.155	0.000
Primary	871	27.0	416	12.9	1,937	60.1			1,070	24.4	570	13.0	2,738	62.5		
Secondary	5,743	26.3	3,009	13.8	13,074	59.9			6,392	26.1	3,239	13.3	14,813	60.6		
Higher	1,046	13.6	855	11.1	5,817	75.4			1,082	14.8	858	11.8	5,349	73.4		
**Population group**																
Black African	7,298	26.4	3,791	13.7	16,580	59.9	890.601	0.000	8,232	26.8	4,177	13.6	18,284	59.6	1,000.000	0.000
Coloured	192	18.6	128	12.4	713	69.0			246	19.6	176	14.0	833	66.4		
Indian/Asian	92	12.1	95	12.5	574	75.4			94	11.5	80	9.8	645	78.7		
White	244	6.3	327	8.5	3,289	85.2			240	5.7	347	8.2	3,634	86.1		
**Income**																
No income	807	36.6	274	12.5	1,122	50.9	1,200.000	0.000	579	31.6	240	13.1	1,015	55.4	1,100.000	0.000
Low	6,449	26.0	3,451	13.9	14,862	60.0			7,744	26.3	4,011	13.6	17,693	60.1		
Middle	368	10.6	395	11.4	2,693	77.9			342	10.5	331	10.2	2,583	79.3		
High	203	7.0	221	7.6	2,480	85.4			147	6.0	197	8.1	2,105	85.9		
**Employment**																
Not employed	4,905	26.0	2,444	12.9	11,543	61.1	136.461	0.000	6,558	25.2	3,304	12.7	16,182	62.1	95.314	0.000
Employed	2,921	20.2	1,898	13.2	9,613	66.6			2,255	20.6	1,475	13.5	7,215	65.9		
**Household-level factors**																
**HH members**																
1	2,117	26.1	1,099	13.5	4,895	60.4	70.421	0.000	757	21.3	487	13.7	2,313	65.0	98.794	0.000
2	1,365	21.6	785	12.4	4,158	65.9			1,289	19.7	807	12.3	4,446	68.0		
3	1,119	20.9	677	12.6	3,560	66.5			1,599	22.6	925	13.1	4,554	64.3		
4 and above	3,225	23.8	1,781	13.1	8,544	63.1			5,168	26.1	2,561	12.9	12,084	61.0		
**Children under 18 in HH**																
None	4,135	23.2	2,305	12.9	11,361	63.8	48.661	0.000	2,349	19.8	1,480	12.5	8,018	67.7	252.620	0.000
1	1,299	22.6	755	13.1	3,693	64.3			1,855	22.9	1,065	13.2	5,174	63.9		
2	1,186	22.6	665	12.7	3,405	64.8			2,110	24.9	1,091	12.9	5,264	62.2		
3	815	25.3	453	14.0	1,956	60.7			1,499	27.1	777	14.1	3,249	58.8		
4+	392	30.2	164	12.6	742	57.2			1,001	32.7	366	12.0	1,691	55.3		
**Child hunger**																
No	3,848	20.0	2,603	13.5	12,828	66.5	698.616	0.000	5,058	20.8	3,266	13.4	15,988	65.8	1,400.000	0.000
Yes	1,563	41.0	496	13.0	1,752	46.0			2,661	40.9	844	13.0	3,001	46.1		
No children in HH	2,416	23.6	1,242	12.1	6,577	64.3			1,094	17.7	670	10.8	4,407	71.4		
**Social Grant**																
No	4,405	21.8	2,529	12.5	13,252	65.6	88.228	0.000	2,818	18.5	1,868	12.2	10,571	69.3	375.864	0.000
Yes	3,421	26.0	1,812	13.8	7,905	60.2			5,995	27.6	2,912	13.4	12,825	59.0		
**Health facility**																
Public	1,997	17.5	1,309	11.5	8,123	71.1	901.520	0.000	1,859	16.4	1,386	12.2	8,097	71.4	906.394	0.000
Private	5,342	29.3	2,497	13.7	10,397	57.0			6,470	29.3	2,858	13.0	12,734	57.7		
Both public and private	368	11.4	441	13.7	2,406	74.8			442	13.1	492	14.6	2,434	72.3		
Traditional	119	26.9	94	21.2	230	51.9			41	18.9	43	20.1	131	60.9		
**Medical aid**																
No	7,230	26.0	3,805	13.7	16,804	60.4	721.611	0.000	8,263	26.1	4,259	13.4	19,160	60.5	749.110	0.000
Yes	596	10.9	537	9.8	4,352	79.3			549	10.4	520	9.8	4,236	79.8		
**Community-level factors**																
**Dwelling type**																
Formal	5,674	20.3	3,569	12.8	18,687	66.9	1,000.000	0.000	6,563	20.8	4,016	12.7	20,931	66.4	1,400.000	0.000
Informal	2,152	39.9	772	14.3	2,469	45.8			2,250	41.1	764	13.9	2,466	45.0		
**Access to media**																
No	182	42.7	52	12.2	192	45.0	117.279	0.000	161	42.3	54	14.1	166	43.6	88.412	0.000
Yes	7,644	23.2	4,289	13.0	20,965	63.7			8,652	23.6	4,726	12.9	23,231	63.5		
**Municipality**																
City of Ekurhuleni	2,308	25.9	1,153	12.9	5,465	61.2	153.315	0.000	2,573	26.5	1,173	12.1	5,946	61.3	218.616	0.000
City of Johannesburg	2,551	21.8	1,643	14.0	7,533	64.2			2,909	22.6	1,842	14.3	8,120	63.1		
City of Tshwane	1,815	22.6	923	11.5	5,286	65.9			1,965	21.9	1,064	11.9	5,926	66.2		
Emfuleni	468	23.9	255	13.0	1,236	63.1			565	24.1	319	13.6	1,461	62.3		
Lesedi	49	18.0	29	10.8	193	71.2			63	19.3	41	12.6	220	68.1		
Midvaal	57	21.8	39	14.8	167	63.3			50	20.5	23	9.2	172	70.3		
Merafong	133	26.5	78	15.5	291	58.0			182	29.4	78	12.7	359	58.0		
Mogale City	257	26.0	147	14.9	586	59.2			289	24.9	155	13.3	718	61.8		
Rand West	188	28.4	75	11.3	399	60.3			216	27.9	85	11.0	475	61.2		
**Survey year**																
2009	575	30.4	405	21.4	909	48.1	543.243	0.000	1,105	38.0	531	18.3	1,271	43.7	984.975	0.000
2011	333	36.8	144	15.9	429	47.4			512	37.0	213	15.4	657	47.6		
2013-2014	1,835	22.8	794	9.9	5,416	67.3			1,926	22.0	815	9.3	6,028	68.7		
2015-2016	1,602	19.7	1,208	14.8	5,326	65.5			1,503	17.9	1,263	15.0	5,635	67.1		
2017-2018	1,407	22.7	713	11.5	4,091	65.9			1,530	22.4	843	12.3	4,474	65.3		
2020-2021	1,085	26.9	552	13.7	2,399	59.4			1,163	26.3	558	12.6	2,700	61.1		
2023-2024	990	24.1	527	12.8	2,587	63.0			1,074	25.2	557	13.1	2,633	61.7		

X^2^ = Chi-square; Note ρ-value (ρ): *ρ < 0 001.

**Table 5:** Showing the gSEM of the determinants of life satisfaction levels among male respondents (n = 33,325).

Males						
Life Satisfaction (Outcome)	Coef. (base outcome)	Robust Std Err.	z	P > |Z|	[95% Conf. Interval]	
**Struggling**						
No education	**Ref.**					
Primary	0.251	0.183	1.370	0.170	−0.107	0.609
Secondary	0.304	0.171	1.780	0.075	−0.031	0.638
Higher	0.553	0.181	3.060	0.002	0.199	0.908
Survey years	−0.037	0.017	−2.170	0.030	−0.070	−0.004
Not employed	**Ref.**					
Employed	0.140	0.050	2.780	0.005	0.041	0.239
No income	**Ref.**					
Low	0.436	0.086	5.070	0.000	0.267	0.604
Middle	1.009	0.125	8.050	0.000	0.763	1.255
Higher	0.953	0.150	6.350	0.000	0.659	1.247
_cons	−1.419	0.206	−6.890	0.000	−1.823	−1.015
**Thriving**						
No education	**Ref.**					
Primary	0.124	0.123	1.000	0.316	−0.118	0.365
Secondary	0.060	0.115	0.530	0.599	−0.165	0.285
Higher	0.493	0.122	4.040	0.000	0.254	0.732
Survey years	−0.007	0.011	−0.610	0.543	−0.029	0.015
Not employed	**Ref.**					
Employment	0.075	0.035	2.140	0.033	0.006	0.143
No income	**Ref.**					
Low	0.480	0.058	8.300	0.000	0.367	0.594
Middle	1.462	0.089	16.460	0.000	1.288	1.637
Higher	1.875	0.110	16.970	0.000	1.658	2.091
_cons	0.159	0.136	1.170	0.242	−0.108	0.425
**Survey years**						
No education	**Ref.**					
Primary	−0.062	0.092	−0.670	0.500	−0.243	0.118
Secondary	0.104	0.086	1.220	0.224	−0.064	0.273
Higher	0.138	0.089	1.560	0.120	−0.036	0.313
Not employed	**Ref.**					
Employment	−0.029	0.025	−1.170	0.243	−0.078	0.020
No income	**Ref.**					
Low	0.783	0.033	23.660	0.000	0.718	0.847
Middle	0.945	0.048	19.610	0.000	0.851	1.040
Higher	1.028	0.054	19.170	0.000	0.923	1.133
_cons	3.510	0.093	37.810	0.000	3.328	3.692
var(e.year)	2.476	0.021			2.435	2.517

Note: * p < 0 05, ** p < 0 01, *** p < 0 001.

**Table 6:** Showing the gSEM of the determinants of life satisfaction levels among female respondents (n = 36,989).

		Females				
Life Satisfaction (Outcome)	Coef. (base outcome)	Robust Std. Err.	z	p > |Z|	[95% Conf. Interval]	
**Struggling**						
No education	**Ref.**					
Primary	0.224	0.127	1.760	0.078	−0.025	0.472
Secondary	0.139	0.117	1.180	0.238	−0.091	0.368
Higher	0.396	0.129	3.080	0.002	0.144	0.648
Survey years	0.017	0.014	1.210	0.228	−0.010	0.044
Not employed	**Ref.**					
Employed	0.156	0.045	3.440	0.001	0.067	0.245
No income	**Ref.**					
Low	0.175	0.081	2.160	0.031	0.016	0.334
Middle	0.664	0.123	5.380	0.000	0.422	0.906
Higher	0.932	0.155	6.010	0.000	0.628	1.236
_cons	−1.275	0.152	−8.410	0.000	−1.572	−0.978
**Thriving**						
No education	**Ref.**					
Primary	0.309	0.086	3.600	0.000	0.140	0.477
Secondary	0.131	0.079	1.660	0.096	−0.023	0.285
Higher	0.456	0.088	5.180	0.000	0.284	0.629
Survey years	0.047	0.010	4.740	0.000	0.028	0.066
Not employed	**Ref.**					
Employment	0.011	0.033	0.340	0.733	−0.054	0.077
No income	**Ref.**					
Low	0.221	0.057	3.880	0.000	0.109	0.332
Middle	1.295	0.089	14.590	0.000	1.121	1.469
Higher	1.872	0.118	15.850	0.000	1.640	2.103
_cons	0.205	0.105	1.950	0.052	−0.001	0.412
**Survey years**						
No education	**Ref.**					
Primary	0.118	0.068	1.730	0.083	−0.015	0.251
Secondary	0.354	0.062	5.670	0.000	0.231	0.476
Higher	0.537	0.067	8.030	0.000	0.406	0.668
Not employed	**Ref.**					
Employment	−0.096	0.025	−3.860	0.000	−0.145	−0.047
No income	**Ref.**					
Low	0.672	0.034	20.040	0.000	0.606	0.737
Middle	0.821	0.052	15.870	0.000	0.719	0.922
Higher	0.985	0.056	17.560	0.000	0.875	1.095
_cons	3.321	0.072	45.940	0.000	3.179	3.463
var(e.year)	2.692	0.020			2.653	2.731

Note: * p < 0 05, ** p < 0 01, *** p < 0 001.

**Table 7:** Generalized structural equation modelling for the determinants of life satisfaction as a confirmed factor for the pooled samples of the male respondents (n = 33,325).

		Direct effects				Indirect effects				Total effects			
		Coef.	P > |Z|	[95% Conf. Interval]		Coef.	P > |Z|	[95% Conf. Interval]		Coef.	P > |Z|	[95% Conf. Interval]	
**Effect of highest education on life satisfaction via year of survey**													
Primary	Suffering	Ref.											
	Struggling	0.251	0.170	−0.107	0.609	0.002	0.520	−0.005	0.009	0.253	0.166	−0.105	0.611
	Thriving	0.124	0.316	−0.118	0.365	0.000	0.651	−0.001	0.002	0.124	0.314	−0.117	0.365
Secondary	Suffering	Ref.											
	Struggling	0.304	0.075	−0.031	0.638	−0.004	0.287	−0.011	0.003	0.300	0.079	−0.035	0.634
	Thriving	0.060	0.599	−0.165	0.285	−0.001	0.588	−0.003	0.002	0.060	0.604	−0.165	0.284
Higher	Suffering	Ref.											
	Struggling	0.553	0.002	0.199	0.908	−0.005	0.204	−0.013	0.003	0.548	0.002	0.193	0.903
	Thriving	0.493	0.000	0.254	0.732	−0.001	0.571	−0.004	0.002	0.492	0.000	0.253	0.731
**Effect of employment on life satisfaction via year of survey**													
Employed	Suffering	Ref.											
	Struggling	0.140	0.005	0.041	0.239	0.001	0.321	−0.001	0.003	0.141	0.005	0.043	0.240
	Thriving	0.075	0.033	0.006	0.143	0.000	0.594	−0.001	0.001	0.075	0.032	0.006	0.144
**Effect of income on life satisfaction via year of survey**													
Low	Suffering	Ref.											
	Struggling	0.436	0.000	0.267	0.604	−0.029	0.030	−0.055	−0.003	0.407	0.000	0.238	0.575
	Thriving	0.480	0.000	0.367	0.594	−0.005	0.543	−0.023	0.012	0.475	0.000	0.362	0.588
Middle	Suffering	Ref.											
	Struggling	1.009	0.000	0.763	1.255	−0.035	0.031	−0.067	−0.003	0.974	0.000	0.729	1.219
	Thriving	1.462	0.000	1.288	1.637	−0.007	0.543	−0.028	0.015	1.456	0.000	1.282	1.629
High	Suffering	Ref.											
	Struggling	0.953	0.000	0.659	1.247	−0.038	0.031	−0.073	−0.003	0.915	0.000	0.622	1.207
	Thriving	1.875	0.000	1.658	2.091	−0.007	0.543	−0.030	0.016	1.868	0.000	1.652	2.083

Note: * p < 0 05, ** p < 0 01, *** p < 0 001.

**Table 8:** Generalized structural equation modelling for the determinants of life satisfaction as a confirmed factor for the pooled samples of the female respondents (n = 36,989).

Demographic Determinants		Direct effects				Indirect effects				Total effects			
		Coef.	P > |Z|	[95% Conf. Interval]		Coef.	P > |Z|	[95% Conf. Interval]		Coef.	P > |Z|	[95% Conf. Interval]	
**Effect of highest education on life satisfaction via year of survey**													
Primary	Suffering												
	Struggling	0.224	0.078	−0.025	0.472	0.002	0.321	−0.002	0.006	0.226	0.075	−0.023	0.474
	Thriving	0.309	0.000	0.140	0.477	0.006	0.105	−0.001	0.012	0.314	0.000	0.145	0.484
+ Secondary	Suffering												
	Struggling	0.139	0.238	−0.091	0.368	0.006	0.237	−0.004	0.016	0.144	0.219	−0.086	0.374
	Thriving	0.131	0.096	−0.023	0.285	0.017	0.000	0.008	0.026	0.148	0.062	−0.008	0.303
Higher	Suffering												
	Struggling	0.396	0.002	0.144	0.648	0.009	0.233	−0.006	0.024	0.405	0.002	0.153	0.657
	Thriving	0.456	0.000	0.284	0.629	0.025	0.000	0.013	0.037	0.482	0.000	0.308	0.655
**Effect of employment on life satisfaction via year of survey**													
Employed	Suffering												
	Struggling	0.156	0.001	0.067	0.245	−0.002	0.251	−0.004	0.001	0.155	0.001	0.066	0.243
	Thriving	0.011	0.733	−0.054	0.077	−0.005	0.003	−0.008	−0.002	0.007	0.836	−0.058	0.072
**Effect of income on life satisfaction via year of survey**													
Low	Suffering												
	Struggling	0.175	0.031	0.016	0.334	0.011	0.228	−0.007	0.029	0.186	0.022	0.027	0.345
	Thriving	0.221	0.000	0.109	0.332	0.032	0.000	0.018	0.045	0.252	0.000	0.140	0.364
Middle	Suffering												
	Struggling	0.664	0.000	0.422	0.906	0.014	0.229	−0.009	0.036	0.678	0.000	0.436	0.920
	Thriving	1.295	0.000	1.121	1.469	0.039	0.000	0.022	0.055	1.334	0.000	1.159	1.508
High	Suffering												
	Struggling	0.932	0.000	0.628	1.236	0.016	0.228	−0.010	0.043	0.948	0.000	0.645	1.251
	Thriving	1.872	0.000	1.640	2.103	0.046	0.000	0.026	0.066	1.918	0.000	1.688	2.148

Note: * ρ < 0 05, ** ρ < 0 01, *** ρ < 0 001.

### 2.10. Handling of missing data and bootstrapping in mediation analysis

Missing data in this study were carefully assessed to maintain methodological rigor. Irrelevant variables were excluded, and only relevant ones retained for analysis. The extent of missingness in key variables was examined using descriptive statistics, including frequencies and percentages. To address potential biases, bootstrapping with 5,000 resamples was applied in the Generalized Structural Equation Modeling (GSEM) and mediation analyses, improving the stability and reliability of indirect effect estimates. Chi-square tests confirmed that missing data were randomly distributed and not systematically associated with demographic variables. Visual tools such as charts was used to explore missing data patterns across respondent groups, and these procedures ensured that the mediation analysis remained robust, replicable, and free from distortions, strengthening the credibility of the findings.

### 2.11. Ethics

The Gauteng City-Region Observatory (GCRO) obtained ethical clearance from the Human Research Ethics Committee (Non-Medical) at the University of the Witwatersrand, Johannesburg (clearance number [H19/11/19]). Verbal consent was transparently obtained from participants aged 18 and above. All respondents were informed of the voluntary nature of participation and assured of confidentiality and anonymity throughout the study.

### 2.12. Ethics approval and consent to participate

The Human Research Ethics Committee (non-medical) at the University of the Witwatersrand, through the Johannesburg Research Office, granted ethical clearance to the GCRO organisation for the design, procedures, and survey instruments used in its regular Quality of Life (QoL) surveys. Additionally, the University of the Witwatersrand’s Institutional Review Board (IRB) in Johannesburg reviewed the GCRO QoL survey methodology. Before taking part in the study, all human subjects provided their informed consent, according to the GCRO Field Staff. The Human Research Ethics Committee (non-medical), University of the Witwatersrand, Johannesburg, has examined and approved the protocols and questions for the standard GCRO QoL surveys. This study has no human volunteers; it only uses secondary data. Consequently, no official ethical approval was needed. However, to utilise the 2009–2021 GCRO QoL survey datasets from the GCRO repository (https://www.datafirst.etc.ac.za/dataportal/index.php/collections/GCRO), authorisation was secured by submitting a written request to the GCRO. The study was carried out in accordance with the Declaration of Helsinki and the applicable ethical standards and laws. The Declaration of Helsinki and all applicable ethical rules and regulations were followed in the conduct of the study. The University of the Witwatersrand, Johannesburg GCRO QoL surveys’ Ethics Committee (Project identification code: Round 1 GCRO QoL 2009; Round 2 GCRO QoL 2011; Round 3 GCRO QoL 2013‒2014; Round 4 GCRO QoL 2015‒2016; Round 5 GCRO QoL 2017‒2018; Round 6 GCRO QoL 2020‒2021, and Round 6 GCRO QoL 2021‒2024) approved the protocol. Only the use of the data for this research topic and the publication of the results in a peer-reviewed journal were conditions of the permission granted.

### 2.13. Institutional review board statement

The GCRO obtains ethical clearance from the Human Research Ethics Committee (non-medical) from the University of the Witwatersrand, Johannesburg Research Office. Verbal consent was obtained from the respondents aged 18 + . All respondents were informed about the voluntary nature of participation, including confidentiality and anonymity.

## 3. Results

Table 3 presents the weighted sample of 70,314 respondents stratified by sex (male: 33,325 [47.4%]; female: 36,989 [52.6%]) across survey years (2009–2024) in Gauteng province, South Africa. It also summarizes respondents’ demographic characteristics, organized into three categories: individual-level, household-level, and community-level factors.

### 3.1. Percentage distribution of male and female respondents

The multiple bar chart shows the percentage distribution of respondents by sex across the survey years as shown in Fig 1. Across the survey years, the majority of the respondents who participated in the survey from 2009–2024 were female (Fig 1).

### 3.2. Percentage distribution of life satisfaction by sex

Fig 2 illustrates the distribution of life satisfaction scales, categorized by sex, among individuals residing in Gauteng province, South Africa. The grouped bar chart revealed slight differences in respondents’ reported life satisfaction between males (63.5%) and females (63.3%) (Fig 2). This finding suggests that the slight difference in life satisfaction between males and females in Gauteng may be influenced by social, economic, and cultural factors, yet the minimal gap indicates overall comparable well-being across sexes.

### 3.3. Percentage distribution of respondents’ age by life satisfaction

Fig 3 shows the proportion by age indicating the life satisfaction scale among South Africans residing in Gauteng province across the survey years. From the stacked bar graph, struggling and suffering life satisfaction scales were mostly reported among age groups 18–27 years and 38–47 years. However, thriving life satisfaction level were majorly reported by age groups 28–37 years and 48 + years (Fig 3).

### 3.4. Demographic characteristics of the respondents

For the individual-level, a majority of both male (29.3%) and female (32.6%) respondents were aged 48 years above, and more respondents were found to be educated to the secondary level (males—65.5% and females—66.1%). 83% of both male and female respondents were Black African (Table 3). Moreover, more than half of the respondents reported earning low income (male—74.3%; female – 79.6%) and were not employed (males—56.7%; females—70.4%). By household factors, male (40.7%) and female (53.6%) respondents have 4 and more household members, and more reported that they have no children under 18 (male—53.4%; female—32%). Also, a majority of the respondents reported having no medical aid (males—83.5%; females—85.7%) and attend a private health facility (males—54.7%; females—59.6%). Furthermore, when considering community-level factors, most respondents indicated that they lived in a formal dwelling place (males—83.8%; females—85.2%), have access to media (males—98.7%; females—99.0%), and are resident in major municipalities of Gauteng—City of Ekurhuleni: male (26.8%); female (26.2%); City of Johannesburg: male (35.2%) and female (34.2%); and City of Tshwane: male (24.1%) and female (24.2%) (Table 3).

### 3.5. Respondent’s life satisfaction and associated factors (Individual-, Household-, and Community-Levels)

Table 4 presents the association between life satisfaction and factors-related indicators (individual-level, household-level, and community-level), stratified by sex using Chi-square. The results showed that there is a significant association between life satisfaction levels and individual-level factors (age group, education, population group, income, and employment) (p < 0 001) (Table 4). Regarding household factors, the Chi-square findings revealed that there is a significant association between life satisfaction levels and household-level factors (HH members, children under 18 in HH, child hunger, social grant, health facility, and medical aid) (p < 0 001) (Table 4). Further, concerning community-level factors, the Chi-square results showed that there is a significant association between life satisfaction levels and community-level factors (dwelling type, access to media, municipality, and survey year) (p < 0 001) (Table 4).

### 3.6. Generalized structural equation modelling (gSEM)

The Generalized Structural Equation Modelling (gSEM) presented the estimates of the model parameters to infer the underlying relationships of the direct, indirect, and total effects to understand the relationships between the explanatory variables and life satisfaction. Tables 5 and 6 present the analysis of independent variables (with reference categories) and life satisfaction levels using gSEM, which comprehensively accounts for both direct and indirect effects.

#### 3.6.1. GSEM showing the determinants and life satisfaction among male respondents.

Table 5 shows the analysis of the determinants (explanatory variables) and life satisfaction levels. As shown in Table 5, the findings revealed that demographic factors (education- higher: β = 0.553, SE = 0.181, 95% CI [0.199–0.908], ρ < 0.002), employment- working: β = −0.037, SE = 0.017, 95% CI [−0.070–0.004], ρ < 0.030), and income- low: β = 0.436, SE = 0.086, 95% CI [0.267–0.604], ρ < 0.000; middle: β = 1.009, SE = 0.125, 95% CI [0.763–1.255], ρ < 0.000); higher: β = 0.953, SE = 0.150, 95% CI [0.659–1.247], ρ < 0.000) significantly predicted struggling life satisfaction levels (Table 5). Also for thriving life satisfaction, demographic factors such as education (higher: β = 0.493, SE = 0.122, 95% CI [0.254–0.732], ρ < 0.000), employment (working: β = 0.075, SE = 0.035, 95% CI [0.006–0.143], ρ < 0.033), income (low: β = 0.480, SE = 0.058, 95% CI [0.367–0.594], ρ < 0.000; middle: β = 1.462, SE = 0.089, 95% CI [1.288–1.637], ρ < 0.000; higher: β = 1.875, SE = 0.110, 95% CI [1.658–2.091], ρ < 0.000) significantly predicted thriving life satisfaction levels (Table 5).

#### 3.6.2. GSEM showing the determinants and life satisfaction among female respondents.

Table 6 shows the analysis of the determinants (explanatory variables) and life satisfaction levels. As shown in Table 6, the findings revealed that demographic factors (education- higher: β = 0.396, SE = 0.129, 95% CI [0.144–0.648], p < 0.002), employment- employed: β = −0.156, SE = 0.045, 95% CI [0.067–0.245], p < 0.001), and income- low: β = 0.175, SE = 0.081, 95% CI [0.016–0.334], p < 0.031; middle: β = 0.664, SE = 0.123, 95% CI [0.422–0.906], p < 0.000); higher: β = 0.932, SE = 0.155, 95% CI [0.628–1.236], p < 0.000) predicted struggling life satisfaction levels (Table 6). Also, for thriving life satisfaction, the findings showed that demographic factors such as education (primary: β = 0.309, SE = 0.086, 95% CI [0.140–0.477], ρ < 0.000; higher: β = 0.456, SE = 0.088, 95% CI [0.284–0.629], p < 0.000), and income (low: β = 0.221, SE = 0.057, 95% CI [0.109–0.332], p < 0.000; middle: β = 1.295, SE = 0.089, 95% CI [1.121–1.469], p < 0.000; high: β = 1.872, SE = 0.118, 95% CI [1.640–2.103], p < 0.000) significantly predicted thriving life satisfaction levels (Table 6).

### 3.7. Pathway model (pathway analysis) of the structural equation modelling (SEM)

The pathway model of the structural equation modelling (SEM) showed the impact of selected determinants (education, employment, income) on life satisfaction, with survey years, to estimate the direct and indirect paths (Figs 4 and 5).

#### 3.7.1. Pathway model of the determinants influencing life satisfaction among male respondents.

Fig 4 shows the pathway model to illustrate the relationship between the independent variables (demographic factors and year) and the dependent variables (life satisfaction) by sex (males) (Fig 4).

#### 3.7.2. Pathway model of the determinants influencing life satisfaction among female respondents.

Fig 5 below shows the pathway model to illustrate the relationship between the independent variables (demographic factors and year) and the dependent variables (life satisfaction) by sex (females) (Fig 5).

### 3.8. Generalized structural modelling (gSEM) analyses for male respondents (n = 33,325)

Tables 7 and 8 involves the generalized structural equation modelling (gSEM), which indicated the strength and direction of the relationships between the determinants and life satisfaction and the effects they have on each other, as direct, indirect and total effects.

#### 3.8.1. Generalized structural modelling (gSEM) analyses for male respondents (n = 33,325).

Table 7 presents the effects of education, employment, and income on life satisfaction (suffering, struggling, thriving). Our results show that higher education, employment, and income significantly enhance life satisfaction (p < 0.000). Higher education directly influences life satisfaction, promoting struggling and thriving among males, while employment status notably increases life satisfaction for those struggling or thriving. Income levels (low, middle, high) also positively affect life satisfaction (p < 0.000). Indirectly, higher education shapes employment status, which in turn influences life satisfaction (p < 0.000). The combined direct and indirect effects highlight the comprehensive role of higher education, employment, and income in improving life satisfaction. Overall, employment status emerges as a key driver, directly boosting life satisfaction and amplifying the effects of education and income.

#### 3.8.2. Generalized structural modelling (gSEM) analyses for female respondents (n = 36,989).

Table 8 presents the effects of demographic factors—education, employment, and income—on life satisfaction (suffering, struggling, and thriving) among female respondents. Significant positive total effects were observed for struggling and thriving life satisfaction across higher education, employment status, and income levels (p ≤ 0.000). Education at all levels (primary, secondary, higher) directly increased struggling and thriving life satisfaction, as did employment and income status (p ≤ 0.000). Indirectly, education influenced life satisfaction by improving employment status, which in turn raised income levels, both contributing to higher life satisfaction (p ≤ 0.000). The combined direct and indirect pathways highlight the comprehensive impact of education, employment, and income on life satisfaction among females.

#### 3.8.3. Percentage distribution of GSEM mediation analysis of life satisfaction among male and female respondents.

Table 9 below shows SEM mediation percentages by gender, highlighting differences in life satisfaction determinants in Gauteng. Income was the strongest predictor, with high-income individuals reporting the highest total effects for both sexes. Thriving high-income women had a total effect of 1.918 (p < 0.001) with 2.4% mediation; men had slightly higher effects with similar mediation. Education positively influenced life satisfaction, especially at higher levels. Among thriving women, 5.2% of higher education’s effect was mediated by survey year, compared to a lower rate for men. Secondary education for thriving women showed the highest mediation (11.5%), reflecting sensitivity to historical and policy shifts; men showed lower, more stable mediation. Employment effects differed by gender: for struggling women, employment increased life satisfaction (0.155, p = 0.001) with negligible mediation (−1.3%), while men had stronger direct and slightly higher indirect effects mediated by survey year. Overall, mediation was low (< 5%) except for secondary education among thriving women, indicating socio-economic factors primarily drive life satisfaction with modest gendered mediation by temporal and policy changes.

**Table 9:** GSEM Mediation Percentages for Life Satisfaction Determinants among Male (n = 33,325) and Female (n = 36,989) Respondents.

Demographic determinants	Males						Females				
	Category	Direct effects	Indirect effects	Total effect	% Mediated	Significance (Total)	Direct effects	Indirect effects	Total effect	% Mediated	Significance (Total)
Education (primary)	Suffering	**RC**	**RC**	**RC**	**RC**	**RC**	**RC**	**RC**	**RC**	**RC**	**RC**
	Struggling	0.251	0.002	0.253	0.79%	0.166	0.224	0.002	0.226	0.88%	0.075
	Thriving	0.124	−0.001	0.124	−0.81%	0.314	0.309	0.006	0.314	1.91%	0.000
Education (secondary)	Suffering	**RC**	**RC**	**RC**	**RC**	**RC**	**RC**	**RC**	**RC**	**RC**	**RC**
	Struggling	0.304	−0.004	0.300	−1.33%	0.079	0.139	0.006	0.144	4.17%	0.219
	Thriving	0.060	−0.003	0.060	−5.00%	0.604	0.131	0.017	0.148	11.9%	0.062
Education (higher)	Suffering	**RC**	**RC**	**RC**	**RC**	**RC**	**RC**	**RC**	**RC**	**RC**	**RC**
	Struggling	0.553	−0.005	0.548	−0.91%	0.002	0.396	0.009	0.405	2.22%	0.002
	Thriving	0.493	−0.004	0.492	−0.81%	0.000	0.456	0.025	0.482	5.19%	0.000
Employment (employed)	Suffering	**RC**	**RC**	**RC**	**RC**	**RC**	**RC**	**RC**	**RC**	**RC**	**RC**
	Struggling	0.140	−0.001	0.141	−0.71%	0.005	0.156	−0.002	0.155	−1.29%	0.001
	Thriving	0.075	−0.001	0.075	−1.33%	0.032	0.011	−0.002	0.007	−28.57%	0.836
Income (low)	Suffering	**RC**	**RC**	**RC**	**RC**	**RC**	**RC**	**RC**	**RC**	**RC**	**RC**
	Struggling	0.436	0.030	0.407	7.37%	0.000	0.175	0.007	0.186	3.76%	0.022
	Thriving	0.480	0.012	0.475	2.53%	0.000	0.221	0.018	0.252	7.14%	0.000
Income (middle)	Suffering	**RC**	**RC**	**RC**	**RC**	**RC**	**RC**	**RC**	**RC**	**RC**	**RC**
	Struggling	1.009	0.035	0.974	3.59%	0.000	0.664	0.014	0.678	2.06%	0.000
	Thriving	1.462	0.015	1.456	1.03%	0.000	1.295	0.039	1.334	2.92%	0.000
Income (higher)	Suffering	**RC**	**RC**	**RC**	**RC**	**RC**	**RC**	**RC**	**RC**	**RC**	**RC**
	Struggling	0.953	0.031	0.915	3.39%	0.000	0.932	0.016	0.948	1.69%	0.000
	Thriving	1.875	0.016	1.868	0.86%	0.000	1.872	0.066	1.918	3.44%	0.000

Note: * p < 0 05, ** p < 0 01, *** p < 0 001.

#### 3.8.4. Key gendered findings.

Income is the strongest predictor of life satisfaction for both men and women, with only modest influence from historical or policy changes. Education positively affects life satisfaction across genders, but secondary education among women shows the greatest responsiveness to socio-political shifts. Employment enhances life satisfaction for both sexes, with more stable and slightly better mediation for men; women’s gains are most evident among vulnerable groups, showing little policy-driven change over time. Overall, mediation effects remain small for most socio-economic factors, though gender differences appear in education and employment.

## 4. Discussion

This study identified key determinants of life satisfaction among adults in Gauteng, South Africa, highlighting the influence of age, education, income, employment, household size, medical aid, and media access. While gender differences were modest, women generally reported higher satisfaction levels, shaped by cultural and socioeconomic factors. The findings support the U-curve hypothesis, with life satisfaction lowest in midlife and higher in early adulthood and after 48 years [66–68]. Regional variations persist, as life satisfaction declines with age in many low- and middle-income contexts due to economic and social instability [69–78]. These findings underscore the importance of addressing employment, education, and health access while considering gendered and age-specific needs to improve life satisfaction outcomes. Policymakers should implement age-targeted social programs, including financial literacy training, employment support, and healthcare access for middle-aged adults, and ensure social engagement and support systems for older adults to maintain high life satisfaction.

Career fulfilment, job satisfaction, financial stability, and concerns about retirement and debt significantly shape life satisfaction in middle age [70]. Supportive family and social relationships improve satisfaction, while conflict and poor work-life balance reduce it [76,79,80]. Personal development opportunities and a sense of purpose further enhance well-being [79,80]. For older adults, life satisfaction depends on health, financial security, social ties, living conditions, and healthcare access, with good health and economic stability promoting satisfaction and chronic illness or financial strain diminishing it [77,81]. A strong sense of community and accessible healthcare are also vital [77]. This study found significant associations between individual-, household-, and community-level factors and life satisfaction, underscoring their interconnected roles. Positive links between personal achievement, household stability, and community resources reinforce the importance of social cohesion. These findings support integrated policies addressing inequality, promoting equity, and advancing sustainable development goals [1,78]. Interventions targeting these key factors can meaningfully enhance life satisfaction across age groups. Governments and local authorities can promote workplace wellness programmes, career development initiatives, and retirement planning support. Community-based programmes that strengthen social networks, provide accessible healthcare, and improve housing conditions can enhance well-being across age groups.

Younger and older adults report higher life satisfaction than middle-aged individuals, reflecting differences in life stages, responsibilities, and stress levels [1,82]. Higher education improves life satisfaction by enhancing job prospects, income, and problem-solving abilities [82,83]. Cultural background and social identity also shape satisfaction through differing expectations and support systems [1,2]. Although higher income improves life satisfaction, its impact decreases once basic financial needs are met and access to essential resources is secured [1,24]. Employment contributes more to life satisfaction than unemployment by providing financial security, purpose, and social interaction [1,17]. In South Africa, key drivers of life satisfaction include higher income, stable employment, good health, education, supportive relationships, and low fear of crime [1,24]. Smaller households report greater satisfaction owing to reduced financial strain [84,85], and medical insurance is strongly associated with well-being [86,87]. Higher household income improves living conditions, healthcare, and education opportunities [57,87], while social grants help ease financial burdens in low-income households [1,24]. Access to media enhances life satisfaction by offering information, entertainment, and social connection [88,89]. Lastly, stable, quality housing contributes to security and life satisfaction, whereas poor housing diminishes it [1,90,91]. Targeted interventions should focus on improving access to quality education, equitable employment opportunities, and financial support for low-income households. Expanding affordable healthcare, social grants, and housing programmes can reduce inequality and enhance well-being. Policies promoting safe neighborhoods and community engagement can further support life satisfaction.

Community-level factors such as safety, housing quality, media access, and public amenities play a crucial role in shaping life satisfaction for both men and women in South Africa [88–91]. Access to media fosters connection and reduces isolation, while safe, well-maintained housing offers stability and improves psychological well-being. Communities with strong social support networks, clean environments, and accessible facilities report higher life satisfaction [1,92]. In Gauteng, urban municipalities report greater life satisfaction due to better infrastructure, healthcare, education, and employment opportunities, while rural areas experience lower satisfaction due to limited services and resources [1,24]. These findings underscore the importance of physical living conditions, community infrastructure, and social cohesion in promoting life satisfaction across diverse settings. Employment opportunities and safety perceptions impact men’s and women’s life satisfaction differently. Urban areas offer diverse jobs, boosting life satisfaction, while rural areas have limited, gender-specific employment [1,24]. Higher urban crime rates particularly affect women’s safety and satisfaction [92]. Stronger community ties and traditional gender roles in rural areas influence life satisfaction differently by gender [93–95]. Tailored policies addressing the distinct needs of men and women in urban and rural settings, improving infrastructure, services, and safety can enhance overall life satisfaction. Urban and rural planning should prioritize equitable infrastructure development, improve public transportation, enhance safety (particularly for women), and expand access to healthcare, education, and media. Programs promoting rural employment opportunities, skills training, and gender-sensitive job placement can reduce disparities.

This study used generalized structural equation modeling (gSEM) to examine how education, employment, and income mediate the relationship between sex and life satisfaction (struggling and thriving). For males, primary to higher education, employment, and income significantly influenced struggling life satisfaction, with higher education, employment, and income boosting thriving life satisfaction consistently across survey years [96–102]. Stable, well-paying jobs and job security were linked to better male life satisfaction. Similarly, these determinants influenced female life satisfaction throughout the survey period, with higher education and income promoting thriving life satisfaction [99,100,103–122]. Education enhances job opportunities, income, and personal achievement, while income reduces stress and offers fulfilling experiences [1,57,89,111]. Employment provides purpose and financial independence, with job quality and work-life balance being key [89,112]. Women’s physical and mental health, along with strong social networks, are crucial for life satisfaction, offering emotional support and belonging [69,99,103,110]. These findings align with prior research showing that demographic factors foster life satisfaction for both sexes, moderated by individual and cultural differences [1,24]. Gender-sensitive policies should focus on improving women’s access to quality education, fair employment, and income-generating opportunities. Workplace policies addressing job quality, flexible schedules, and work-life balance can enhance life satisfaction for both sexes.

Female-headed households often face lower quality of life and greater economic vulnerability than male-headed ones, partly due to commuting challenges that limit employment access [89–94,97,101]. These households rely more on social grants, reflecting gender disparities in urbanization and intersecting with race and ethnicity to affect resource access and well-being [90,91,94]. Programmes targeting female-headed households, such as subsidized childcare, transportation support, skills training, and income support, can reduce economic vulnerability and promote equity. Urban planning should improve accessibility to essential services and employment opportunities. Addressing these inequalities requires targeted policies to support female-headed households, often single-parent or skipped-generation families by enhancing earning capacities and reducing grant dependency [90,98,103,106]. Mapping data from the GCRO (2009–2024) can guide transportation and urban planning to improve accessibility, especially for women, informing strategies to promote gender equality and service access in Gauteng [28–34,98,103]. Income remains the strongest determinant of life satisfaction for both genders, with limited mediation over time, highlighting persistent socio-economic inequalities shaping well-being in South Africa.

Education’s impact on life satisfaction shows clear gender differences. While higher education improves life satisfaction for both sexes, women especially with secondary education are more affected by policy and temporal changes, indicating middle-tier education as a key intervention point [123–125]. Employment effects also differ as men experience more stable and mediated benefits over time, whereas women, particularly those struggling, see direct but less mediated gains. Policies should promote gender-responsive education programmes, support women’s progression beyond secondary education, and address structural labour market barriers. Employment programmes should consider gender-specific needs, including flexible work arrangements, mentorship, and skills development for women [125,126]. Income remains the strongest determinant of life satisfaction for both genders, highlighting persistent socio-economic inequalities shaping well-being. This reflects persistent structural barriers in the labour market for women, where employment alone may not improve quality of life without gender-responsive policies [127,128]. These findings call for gender-sensitive approaches that address the evolving and complex socio-economic inequalities beyond income and education. Broader socio-economic policies addressing income inequality, social protection, and access to essential services are crucial for improving overall life satisfaction across Gauteng.

### 4.1. Further discussion: Implementation science for gender-sensitive psychodemographic and sociology practices

Gender sensitivity promotes respect across sexes, helping to avoid stereotypes and benefit all [113,114]. Gender-transformative interventions challenge harmful norms, especially around masculinity, and are vital to addressing complex inequalities in resources, power, and opportunities [115]. Effective gender-sensitive policies must address specific needs to promote equality and life satisfaction. Implementation science plays a crucial role by translating research into practice, identifying effective interventions, overcoming barriers, and enabling continuous monitoring and adjustment [112,115–117]. Engaging stakeholders ensures relevance and acceptance, supporting sustainable, impactful gender equality initiatives [101,118]. South Africa’s gender equality efforts, including the National Policy Framework and UN Women’s strategy [104,108], seek to close gender gaps, but disparities persist. Gender-sensitive policies that ensure equal access to resources, opportunities, and protections can improve life satisfaction and promote equity [114,115]. Implementation science is essential to making these policies effective and advancing outcomes for all genders [115–118]. Raising awareness and promoting gender equality further enhances life expectations and social well-being.

### 4.2. Strengths and limitations

This study’s key strength lies in its gender-specific analysis of life satisfaction determinants within Gauteng’s social context, providing nuanced insights into male and female experiences. Utilizing the provincially representative GCRO QoL dataset (2009–2024) enhances reliability and generalizability while minimizing self-report biases. Integrating perspectives from sociology, psychology, and demography further deepens understanding of how individual, household, and community factors shape life satisfaction and highlight areas for improvement. However, the cross-sectional design limits causal inference and trend analysis. Future research should adopt longitudinal approaches and advanced methods like multilevel modeling to better capture gender-specific variations over time. The omission of variables such as marital status and religion due to non-response may affect model accuracy, so including these in future surveys is recommended. Overall, this study expands the literature on life satisfaction in Gauteng, offering a comprehensive foundation for future gender-focused research.

### 4.3. Implications for sociology of life satisfaction and pyschodemographic research

Our research shows that social factors mediate the relationship between demographic determinants and life satisfaction. Understanding these determinants aids in creating targeted interventions to improve life satisfaction in South Africa. Gender equality plays a crucial role by challenging traditional roles, improving resource access, and promoting fairness, which correlates with higher life satisfaction for both men and women [70,110]. Gender equality empowers women’s decision-making and reduces pressures on men to conform, fostering diverse interests and careers. Psychodemographic and sociological research should guide gender-sensitive interventions that address the distinct needs of both sexes. Implementing evidence-based policies and programmes based on these insights can build a more equitable society where everyone can achieve greater well-being.

## 5. Conclusions

Our study examined how demographic determinants and gender influence life satisfaction among men and women. We found strong links between education, employment, income, and gendered experiences of struggling or thriving life satisfaction. Gender moderated the association between municipalities and suffering-related life satisfaction, reflecting how lived experiences and social roles vary by location. Including gender in life satisfaction research is vital, as it shapes career, family, and identity outcomes. Promoting gender equality and challenging stereotypes fosters a society with equal rights and opportunities for all [100,101]. Gender-responsive programmes enhance well-being across genders [100,101]. Although socio-economic factors directly affect life satisfaction in Gauteng, the impact of historical and policy changes remains modest, especially for women. These results highlight the need for targeted, gender-sensitive policies that address structural inequalities and strengthen public policy’s mediating role. Based on these findings, we propose three key recommendations for South Africa:

### Research and data collection

The Gauteng City Region Observatory (GCRO) should integrate variables from sociology, psychology, and psychodemographics into its surveys to enable intersectional analyses of gendered life satisfaction and well-being. This will enhance the understanding of how gender intersects with socio-economic and spatial inequalities, informing more targeted, inclusive policy responses.

### Targeted intervention and programme initiatives

Municipalities facing economic and social decline need inclusive economic empowerment initiatives for both women and men. Concurrently, public sensitisation campaigns on gender roles and stereotypes should promote equality, enabling individuals to pursue personal and professional goals free from traditional constraints.

### Policy advocacy

Provinces must adopt comprehensive, gender-responsive policy reforms addressing systemic inequalities. Strengthening and funding gender advocacy networks is essential to ensure sustained support for the diverse needs of women and men and to advance equitable socio-economic development.

## Supporting information

S1 File Do files as stata file. (ZIP)

## Abbreviations

QoL: Quality of Life
GCRO QoLS: Gauteng City-Region Observatory’s Quality of Life Survey
RSA: Republic of South Africa
SALGA: South African Local Government Association
GCR: Gauteng City Region
SWLS: Satisfaction with Life Scale
gSEM: generalized Structural Equation Modelling
HH: household
SASSA: South African Social Security Agency
GCRO: the Gauteng City Region Observatory.
