# Supplementary material for: “Gender dimensions of quality of life”: The determinants of life satisfaction among South Africans residing in the Gauteng province—Using a multilevel psychodemographic analysis of the GCRO’s quality of life survey (2009–2024)
Source: PLoS One. 2025 Sep 15;20(9):e0332626. doi: 10.1371/journal.pone.0332626 (PMC12435731; doi:10.1371/journal.pone.0332626)
Supplement: Attachment — Submitted filename: PONE-D-25-37543-review.pdf [file pone.0332626.s003.pdf]

PLOS ONE

“Gender Dimensions of Quality of Life”: The Determinants of Life Satisfaction Among South Africans Residing in the Gauteng Province—Using a Multilevel Psychodemographic Analysis of the GCRO’s Quality of Life Survey (2009–2024)

--Manuscript Draft--

**Manuscript Number:**	PONE-D-25-37543
**Article Type:**	Research Article
**Full Title:**	“Gender Dimensions of Quality of Life”: The Determinants of Life Satisfaction Among South Africans Residing in the Gauteng Province—Using a Multilevel Psychodemographic Analysis of the GCRO’s Quality of Life Survey (2009–2024)
**Short Title:**	“Gender Dimensions of Quality of Life”: Using a Multilevel Psychodemographic Analysis.
**Corresponding Author:**	Monica Ewomazino Akokuwebe, Ph.D.<br>Witwatersrand Health Sciences: University of the Witwatersrand Johannesburg Faculty of Health Sciences<br>Johannesburg, Gauteng Province SOUTH AFRICA
**Keywords:**	Determinants; gender dimensions; Life satisfaction; Psychodemographics; South Africa
**Abstract:**	This study aimed to assess the gender-related differences in life satisfaction among South Africans, by comparing levels of life satisfaction between males and females. A total of 70,314 (ages 18+) respondents comprising 33,325 males and 36,989 females were sampled from the GCRO (Gauteng City-Region Observatory) cross-sectional data. The data were analyzed using Pearson Multiple correlation and multi-group mediation analysis, using STATA version 18.0. The findings demonstrated a significant association between life satisfaction levels (suffering, struggling and thriving) and individual-level factors (age group, education, population group, income, and employment) (p < 0 001). Struggling or thriving life satisfaction levels are associated with education, employment, income, and the survey period (2009–2024). Thriving respondents experience high life satisfaction, well-being, and optimism, supported by access to resources and networks. The mediation analysis revealed that demographic determinants significantly predicted struggling life satisfaction levels among male respondents. Higher education (β = 0.553, SE = 0.181, 95% CI [0.199–0.908], p < 0.002), working employment status (β = −0.037, SE = 0.017, 95% CI [−0.070–0.004], p < 0.030), and different income levels (low: β = 0.436, SE = 0.086, 95% CI [0.267–0.604], p < 0.000; middle: β = 1.009, SE = 0.125, 95% CI [0.763–1.255], p < 0.000; higher: β = 0.953, SE = 0.150, 95% CI [0.659–1.247], p < 0.000) were all significant predictors. Also, among the female respondents, the findings revealed that struggling life satisfaction significantly predicted demographic determinants (education-higher: β = 0.396, SE = 0.129, 95% CI [0.144–0.648], p < 0.002), employment-employed: β = −0.156, SE = 0.045, 95% CI [0.067–0.245], p < 0.001), and income-low: β = 0.175, SE = 0.081, 95% CI [0.016–0.334], p < 0.031; middle: β = 0.664, SE = 0.123, 95% CI [0.422–0.906], p < 0.000); higher: β = 0.932, SE = 0.155, 95% CI [0.628–1.236], p < 0.000). Also, income has indirect effects on life satisfaction among male respondents, while education and income have direct effects on life satisfaction among female respondents. These findings inferred that life satisfaction of females was different from males in South Africa. Therefore, gender-sensitive initiative programmes should be optimized to address gender-specific needs, so as to strive for more and effective gender equality approaches to improve the overall well-being of South Africans residing in Gauteng province.
**Order of Authors:**	Monica Ewomazino Akokuwebe, Ph.D.
	Salmon Likoko, M.Sc.
	Shamsunisaa Miles-Timotheus, M.Sc.
	Moyahabo Mabala, M.Sc.
**Additional Information:**	

Powered by Editorial Manager® and ProduXion Manager® from Aries Systems Corporation

Question	Response
**Financial Disclosure**<br><br>Enter a financial disclosure statement that describes the sources of funding for the work included in this submission. Review the submission guidelines for detailed requirements. View published research articles from *PLOS ONE* for specific examples.<br><br>This statement is required for submission and **will appear in the published article** if the submission is accepted. Please make sure it is accurate.<br><br>**Funded studies**<br>Enter a statement with the following details:<br>• Initials of the authors who received each award<br>• Grant numbers awarded to each author<br>• The full name of each funder<br>• URL of each funder website<br>• Did the sponsors or funders play any role in the study design, data collection and analysis, decision to publish, or preparation of the manuscript?<br><br>Did you receive funding for this work?	The author(s) received no specific funding for this work.
**Competing Interests**<br><br>Use the instructions below to enter a competing interest statement for this submission. On behalf of all authors, disclose any competing interests that could be perceived to bias this work—acknowledging all financial support and any other relevant financial or non-financial competing interests.<br><br>This statement is required for submission and **will appear in the published article** if the submission is accepted. Please make sure it is accurate and that any funding sources listed in your Funding Information later in the submission form are also	No competing interests.

Powered by Editorial Manager® and ProduXion Manager® from Aries Systems Corporation

declared in your Financial Disclosure statement.<br><br>View published research articles from *PLOS ONE* for specific examples.<br><br>**NO authors have competing interests**<br><br>Enter: *The authors have declared that no competing interests exist.*<br><br>**Authors with competing interests**<br><br>Enter competing interest details beginning with this statement:<br><br>*I have read the journal's policy and the authors of this manuscript have the following competing interests: [insert competing interests here]*<br><br>* typeset	
**Ethics Statement**<br><br>Enter an ethics statement for this submission. This statement is required if the study involved:<br><br>• Human participants<br>• Human specimens or tissue<br>• Vertebrate animals or cephalopods<br>• Vertebrate embryos or tissues<br>• Field research<br><br>Write "N/A" if the submission does not require an ethics statement.<br><br>General guidance is provided below. Consult the submission guidelines for detailed instructions. **Make sure that all information entered here is included in the Methods section of the manuscript.**	N/A – Ethical approval was not required for this study as it involved secondary data analysis using publicly available datasets obtained from the Gauteng City-Region Observatory (GCRO) for the years 2009 to 2024. The GCRO data were de-identified and made accessible for research purposes, with no direct interaction with human participants or collection of personally identifiable information. As such, the study does not meet the criteria for human subjects research requiring ethical clearance.

Powered by Editorial Manager® and ProduXion Manager® from Aries Systems Corporation

**Format for specific study types**<br><br>**Human Subject Research (involving human participants and/or tissue)**<br>• Give the name of the institutional review board or ethics committee that approved the study<br>• Include the approval number and/or a statement indicating approval of this research<br>• Indicate the form of consent obtained (written/oral) or the reason that consent was not obtained (e.g. the data were analyzed anonymously)<br><br>**Animal Research (involving vertebrate animals, embryos or tissues)**<br>• Provide the name of the Institutional Animal Care and Use Committee (IACUC) or other relevant ethics board that reviewed the study protocol, and indicate whether they approved this research or granted a formal waiver of ethical approval<br>• Include an approval number if one was obtained<br>• If the study involved *non-human primates*, add *additional details* about animal welfare and steps taken to ameliorate suffering<br>• If anesthesia, euthanasia, or any kind of animal sacrifice is part of the study, include briefly which substances and/or methods were applied<br><br>**Field Research**<br><br>Include the following details if this study involves the collection of plant, animal, or other materials from a natural setting:<br>• Field permit number<br>• Name of the institution or relevant body that granted permission	
**Data Availability**<br><br>Authors are required to make all data underlying the findings described fully available, without restriction, and from the time of publication. PLOS allows rare exceptions to address legal and ethical concerns. See the PLOS Data Policy and FAQ for detailed information.	Yes - all data are fully available without restriction

A Data Availability Statement describing where the data can be found is required at submission. Your answers to this question constitute the Data Availability Statement and **will be published in the article**, if accepted.<br><br>**Important:** Stating 'data available on request from the author' is not sufficient. If your data are only available upon request, select 'No' for the first question and explain your exceptional situation in the text box.<br><br>Do the authors confirm that all data underlying the findings described in their manuscript are fully available without restriction?	
**Describe where the data may be found in full sentences. If you are copying our sample text, replace any instances of XXX with the appropriate details.**<br><br>• If the data are **held or will be held in a public repository**, include URLs, accession numbers or DOIs. If this information will only be available after acceptance, indicate this by ticking the box below. For example: *All XXX files are available from the XXX database (accession number(s) XXX, XXX.).*<br>• If the data are all contained **within the manuscript and/or Supporting Information files**, enter the following: *All relevant data are within the manuscript and its Supporting Information files.*<br>• If neither of these applies but you are able to provide **details of access elsewhere**, with or without limitations, please do so. For example:<br><br>*Data cannot be shared publicly because of [XXX]. Data are available from the XXX Institutional Data Access / Ethics Committee (contact via XXX) for researchers who meet the criteria for access to confidential data.*<br><br>*The data underlying the results presented in the study are available from (include the name of the third party*	Data Availability Statement: Data are from the Gauteng City-Region Observatory (GCRO) and the dataset is open to qualified researchers free of charge. In order to access the data from the GCRO, a written request was submitted to the GCRO and permission was granted to use the data for this survey. To request access to the dataset, please apply at https://www.datafirst.uct.ac.za/dataportal/index.php/catalog/GCRO/?page=1&sort_by=title&sort_order=asc&ps=15&r epo=GCRO.<br><br>Supplementary Information for Data and Materials: All data generated or analysed during this study are included in this published article [and its supplementary information files].

and contact information or URL).<br>• This text is appropriate if the data are owned by a third party and authors do not have permission to share the data.<br><br>* typeset	
Additional data availability information:	Tick here if the URLs/accession numbers/DOIs will be available only after acceptance of the manuscript for publication so that we can ensure their inclusion before publication.

Powered by Editorial Manager® and ProduXion Manager® from Aries Systems Corporation

Manuscript

Click here to access/download;Manuscript;6th_Monica_Salmon GCRO

"Gender Dimensions of Quality of Life": The Determinants of Life Satisfaction Among South Africans Residing in the Gauteng Province—Using a Multilevel Psychodemographic Analysis of the GCRO's Quality of Life Survey (2009–2024)

Monica Ewomazino Akokuwebe [1,*], Salmon Likoko [2], Shamsunisaa Miles-Timotheus [3], Moyahabo Mabala [4]

[1]SAMRC Developmental Pathways for Health Research Unit, Department of Paediatrics, School of Clinical Medicine, Faculty of Health Sciences, University of the Witwatersrand, Johannesburg, South Africa; monica.akokuwebe@wits.ac.za

[2]Demography and Population Studies, School of Social Sciences, University of Witwatersrand, Johannesburg, South Africa; likokosalmon@gmail.com

[3]Gauteng City-Region Observatory (GCRO), University of the Witwatersrand, University of Johannesburg, Johannesburg, South Africa; shamsunisaa.miles-timotheus@gcro.ac.za

[4]Group Finance, City of Joburg, Jorrisen Place, 66 Jorrisen Street, Johannesburg 2017, South Africa; moyahabotom@yahoo.com

*Correspondence: monica.akokuwebe@wits.ac.za

Abstract:

This study aimed to assess the gender-related differences in life satisfaction among South Africans, by comparing levels of life satisfaction between males and females. A total of 70,314 (ages 18+) respondents comprising 33,325 males and 36,989 females were sampled from the GCRO (Gauteng City-Region Observatory) cross-sectional data. The data were analyzed using Pearson Multiple correlation and multi-group mediation analysis, using STATA version 18.0. The findings demonstrated a significant association between life satisfaction levels (suffering, struggling and thriving) and individual-level factors (age group, education, population group, income, and employment) ($p < 0\ 001$). Struggling or thriving life satisfaction levels are associated with education, employment, income, and the survey period (2009–2024). Thriving respondents experience high life satisfaction, well-being, and optimism, supported [illegible] access to resources and networks. The mediation analysis revealed that demographic determinants significantly predicted struggling life satisfaction levels among male respondents. Higher education ($\beta = 0.553$, SE = 0.181, 95% CI [0.199–0.908], $p < 0.002$), working employment status ($\beta = -0.037$, SE = 0.017, 95% CI [−0.070–0.004], $p < 0.030$), and different income levels (low: $\beta = 0.436$, SE = 0.086, 95% CI [0.267–0.604], $p < 0.000$; middle: $\beta = 1.009$, SE = 0.125, 95% CI [0.763–1.255], $p < 0.000$; higher: $\beta = 0.953$, SE = 0.150, 95% CI [0.659–1.247], $p < 0.000$) were all significant predictors. Also, among the female respondents, the findings revealed that struggling life satisfaction significantly predicted demographic determinants (education- higher: $\beta = 0.396$, SE = 0.129, 95% CI [0.144–0.648], $p < 0.002$), employment- employed: $\beta = -0.156$, SE = 0.045, 95% CI [0.067–0.245], $p < 0.001$), and income- low: $\beta = 0.175$, SE = 0.081, 95% CI [0.016–0.334], $p < 0.031$; middle: $\beta = 0.664$, SE = 0.123, 95% CI [0.422–0.906], $p < 0.000$); higher: $\beta = 0.932$, SE = 0.155, 95% CI [0.628–1.236], $p < 0.000$). Also, income has indirect effects on life satisfaction among male respondents, while education and income have direct effects on life satisfaction among female respondents. These findings inferred that life satisfaction of females was different from males in South Africa. Therefore, gender-sensitive initiative programmes should be optimized to address gender-specific needs, so as to strive for more and effective gender equality approaches to improve the overall well-being of South Africans residing in Gauteng province.

Keywords: determinants; gender dimensions; life satisfaction; psychodemographics; South Africa

1. Introduction

Understanding Quality of Life (QoL) is crucial for improving subjective life evaluation. As identified by psycho-demographics, life satisfaction is a key indicator of socio-economic well-being, which explores how population characteristics and mental processes shape subjective well-being [1–4]. This approach, which emphasizes the role of QoL in shaping subjective well-being, views life satisfaction as an interplay between external conditions and internal psychological processes [3–5]. Psychodemographic research examines how factors such as demographic characteristics, gender identity, language, socialization, racial group membership, and cultural individuality influence life satisfaction and other social indicator [5–7]. Life satisfaction reflects how individuals compare their current circumstances to their ideal life, considering both material and spiritual aspects, and is essential for a happy life [8,9]. Incorporating life satisfaction measures into South Africa's strategic policy framework could enhance the monitoring of demographic indicators, including education, employment, income, and resource accessibility [10,11].

The Healthy South Africa Strategic Policy Framework includes initiatives to improve health services [10,11]. In South Africa's multicultural society, life satisfaction is influenced by various cultural and social factors, with social status strongly associated with well-being and highlighting persistent disparities among population groups [12–17]. Despite development, South Africa remains unequal, with a predicted Gini coefficient of 0.63 in 2024 [11]. Møller's study found that improved living standards for Black South Africans enhanced their quality of life, yet they remained less satisfied than other groups [7]. Economic challenges, rising unemployment, and inequality impact well-being despite social improvements [14,16]. Political changes and the COVID-19 pandemic have affected life satisfaction, influencing public trust and governance [4]. Generational shifts have significantly shaped expectations and experiences, as documented by Møller [6] and subsequent studies. Recent comparative analyses highlight changing patterns and determinants of life satisfaction over time [3–6].

Life satisfaction represents a complex interplay between individual wants and material circumstances, mediated by a comparative process [8,12]. This process spans various aspects of human experience, including happiness, subjective well-being, economic stability, and social relationships [1,2]. Studies conducted in South Africa have reported varied findings on the impact of economic factors on life satisfaction [18,19]. Owusu et al. [14], in particular, underscored the need for a nuanced and context-sensitive approach, warning against the tendency to overgeneralize the influence of specific determinants. Although many theoretical and empirical studies exist, none offer universal applicability [20–23], presenting ongoing challenges for research endeavours. In addition, gender-based differences in life satisfaction throughout daily activities introduce complexity in distinguishing selection effects from direct psychodemographic influences [1,2].

Studies in South Africa investigating gender-based differences and social characteristics offer valuable opportunities for further research. Exploring these group distinctions and the mechanisms behind them can enhance our understanding of life satisfaction [19]. Psychodemographic factors significantly affect life satisfaction, particularly during key life stages, with gender differences being a major influence. Previous studies have established a link between cultural, social, and economic integration and how individuals in South Africa evaluate their lives [19,21]. Contemporary studies have demonstrated positive correlations between life satisfaction and factors such as favorable living conditions, economic inclusion, wealth, and access to resources among South African citizens [19,24]. These findings significantly enhance our understanding of the determinants of well-being. Moreover, despite limited resources, urban residents show a preference for a comprehensive system and greater reliance on government services [23,24].

In high-income countries, life satisfaction is linked to age, gender, race, marital status, health, education, and income [19,24]. Family income in Ghana and Asia correlates positively with satisfaction [14,16]. Marriage and childbirth increase life satisfaction, while divorce, job changes, unemployment, and relocation reduce it [23–26]. Similar patterns appear in sub-Saharan Africa, including Tanzania, Ghana, Ethiopia, and Malawi [1,27]. In South Africa, factors such as religion, migration, income, education, social investment, substance use, class status, region, job security, and marital status influence life satisfaction [25–27]. These findings highlight the need for further research on determinants, especially across racial groups [26,27]. Shen et al. [12] and Nyagweta [4] define life satisfaction by living conditions rather than pleasure, suggesting new research directions.

Recent South African studies by Ajaero et al. [24] and Nyagweta [4] examined life satisfaction through social networks, demographics, well-being, and migration, using nationally representative samples despite some methodological limitations. Earlier work by Ebrahim et al. [3] and Posel et al. [8] focused on racial groups and income perceptions, respectively. Factors like limited facility access [1], chronic illness [12], unemployment [27], poor healthcare [2], education gaps [23], poverty [14], and inequality [18] continue to affect life satisfaction. The Gauteng City-Region Observatory Quality of Life Survey (2009–2024) [28–34] further explored these determinants, with this study uniquely emphasizing gender dimensions—a gap in South African research.

Examining the relationship between sex distribution and life satisfaction determinants in South Africa offers insights into factors affecting both male and female populations, including nationals and non-citizens [3,4]. Sex refers to biological distinctions, while gender reflects socially defined roles [3,4]. Unlike most studies using simple linear methods, this study applied multi-level psychodemographic analyses to the 2009–2024 GCRO datasets [4–10]. Previous research often considers limited factors, neglecting the complexity of real-life influences [3,21], and few use longitudinal data to examine diverse groups such as the elderly, migrants, and racial populations [7,8]. This study emphasizes the importance of incorporating multiple predictors across large, diverse samples and highlights the urgent need for gender-specific programmes to improve life satisfaction in South Africa, particularly in Gauteng province.

Several studies have shown that education, work, and societal factors are more strongly associated with male life satisfaction, while female life satisfaction is linked to marital status and the quality of social relationships [12,18]. It is therefore expected that our findings will reflect these gendered differences, addressing a gap in existing research. This study examines life satisfaction by sex distribution among South Africans, with specific aims to: (1) measure life satisfaction across sex and age groups; (2) assess bivariate associations between life satisfaction and demographic determinants by sex; (3) analyze the spatial distribution of life satisfaction by sex across municipalities; and (4) propose a mediation model linking demographic determinants to life satisfaction by sex across survey years.

2. Methods

2.1. Study Area

This study was conducted in Gauteng Province, South Africa's smallest and most urbanized province [35]. South Africa, officially the Republic of South Africa (RSA), has a population of nearly 60 million and borders six countries while surrounding Lesotho. It operates a tripartite capital system: Pretoria (executive), Bloemfontein (judicial), and Cape Town (legislative) [35]. The country is ethnically diverse, with Black South Africans making up over 81% of the population, and Christianity as the predominant religion [35,36]. Despite being an upper-middle-income economy and a member of G20, BRICS, and the Commonwealth, South Africa faces high levels of poverty, unemployment, and inequality, with 25% of its population living on less than US$1.25 per day as of 2008 [36]. A notably youthful demographic,

with 37% aged 14–35, characterizes its population profile [36,37]. Gauteng is South Africa's most populous and urbanized province, with 15 million residents in 2022, accounting for 26.6% of the national population [38,39]. It contributes about a third of the country's GDP, producing R1.59 trillion in 2016 and attracting over a third of internal migrants, while provinces like Limpopo and Eastern Cape experienced outflows [35,37,38]. By 2022, 89.7% of households had access to flush or chemical toilets, up from 86.5% in 2011, reflecting infrastructure progress [40–42]. The province's cosmopolitan character is evident in its diverse languages, with isiZulu (25%), Sesotho (13.4%), Sepedi (12%), English (10.7%), and Setswana (9.8%) widely spoken, alongside all eleven official languages [33,35,37,40].

2.2. Study Design and Data Sour[illegible]e

This study utilizes Quality of Life (QoL) data collected by the Gauteng City-Region Observatory (GCRO) between 2009 and 2024. Specifically, the data come from the GCRO QoL—a cross-sectional sample survey conducted by the GCRO, which is a partnership between the University of Johannesburg (UJ), the University of the Witwatersrand (Wits), the Gauteng Provincial Government (GPG), and the South African Local Government Association (SALGA) [28]. In 2009, the GCRO conducted its first Quality of Life (QoL) survey, a comprehensive endeavours that captured the diverse characteristics of the Gauteng City Region (GCR) in all their complexity. The survey evaluated dimensions such as socioeconomic conditions, perceptions of public services, psychological outlooks, core values, and overall quality of life [28–34]. The resulting data were then used to analyze key performance indicators within the Gauteng province, monitor progress towards the Sustainable Development Goals (SDGs) and other international targets, and support developing strategies, policies, and programs [43–45]. A household survey targeting individuals aged 18 and older in Gauteng was conducted using a multi-stage, stratified cluster sampling method [28–34]. Over time, the Gauteng City-Region Observatory has been at the forefront of social research, administering a sequence of Quality of Life surveys at regular intervals: Round 1 in 2009 [28], Round 2 in 2011 [29], Round 3 in 2013–2014 [30], Round 4 in 2015–2016 [31], Round 5 in 2017–2018 [32], and Round 6 in 2020–2021 [33]. The most recent iteration, Round 7, occurred in 2023–2024 [34]. In terms of spatial sampling, the survey comprehensively covered a large number of enumeration areas, with Round 1 encompassing 5,740, Round 2 including 1,008, Round 3 covering 16,400, Round 4 encompassing 24,889, Round 5 involving 22,220, Round 6 sampling 3,075, and Round 7 surveying more than 13,500 enumeration areas [28–34]. This ongoing research sheds light on the changing dynamics of Gauteng's quality of life. This involved the collection of data from sampled enumeration areas (or selected areas), which were used to represent the broader population in the study. Within each municipality covered by the sampled enumeration areas, clusters were allocated to urban and rural segments in proportion to the population sizes reflected in the sampling frame. Additionally, primary sampling units were assigned to these urban and rural strata based on the number of households recorded in the census, providing a solid basis in empirical evidence for the allocation. The sampled enumeration areas were 86,832 from 2009–2024, and 70,314 samples were extracted from the Round 1 2009 survey to the Round 7 2024 survey. The final sample of 70,314 was stratified into male (33,325) and female (36,989) for intersectional analysis [28–34]. The authors accessed the 2009–2024 GCRO cross-sectional datasets for research purposes on 7 August 2024 via the GCRO website. The authors did not have access to any personally identifiable information of the participants during data collection or subsequent use of the data, as the datasets were secondary sources obtained with permission from the GCRO organization.

2.3. Study Population and Sample Size

The study population comprised South African nationals stratified by sex who resided in the Gauteng province of South Africa. South African nationals refers to individuals who hold South African citizenship. By conceptual definition and operationalization in this study, a South African national is any person born to at least one South African parent who receives citizenship at birth; children born to a legal resident of the country are permitted to obtain South African citizenship only when they reach the age of majority. Also, foreign nationals may be granted citizenship after meeting a

residence requirement (usually five years). This definition was used in line with the South African nationality law detailing the conditions by which a person is a national of South Africa. The primary law governing such nationality requirements is the South African Citizenship Act 1995, which came into force on the 6th of October 1995 [46]. The total number of South African nationals from the 2009–2024 GCRO datasets was 70,314 comprising 33,325 males and 36,990 females for the sample size for this study, after the exclusion of missing variables in the datasets cleaning. The sample size of 70,314 included the age range from 18 to 48+ years [28–34], and this comes after the datasets from different survey years were combined. The data from each year was combined to create a comprehensive dataset, enabling a broader perspective on trends and changes over time. The datasets from 2009–2024 were added together to expand the range of generalization in order to improve the statistical power of analysis for reliable conclusions, allowing comparative analysis for potential causal relationships and control for confounding variables, hence justifying its inclusion [47–49]. In addition, an iteration variable was created to be able to identify changes over time.

2.4. Operational Definition of Concepts

Sex and gender are two different concepts, but they are often used interchangeably [50,51]. Sex refers to biological differences (such as chromosomes, hormones, and reproductive organs) [50] while gender refers to socially constructed roles, behaviours, activities, and expectations associated with femininity and masculinity [51]. Therefore, in this study, we treated sex and gender as distinct constructs. Sex was used to stratify the population into male and female categories, while gender was utilized to explore roles, identities, and societal expectations related to life satisfaction.

2.5. Measures

The GCRO Quality of Life Rounds 1 to 7 (2009–2024) [28–34] survey—Full Questionnaires were included in the field data collection instrument, which comprised questions focused on: (1) demographic details of the enumerated population (population group, sex, age, language), (2) housing (dwelling type, tenure, satisfaction with dwelling, perceived quality of housing and housing allocation), (3) household services (water, sanitation, refuse removal, energy sources), (4) migration and health (including disability), (5) education and employment (including employment sector), (6) community services (amenities, transport, leisure activities, safety and crime), (7) financial data (including debts, income, and social grants), (8) household assets (Telephone, Television, Computer, Radio, Music system, Satellite TV [e.g. MNET, DSTV], Internet connection, Car, Bicycle, Fridge), (9) public participation and governance, (10) perceived personal well-being, and 11) quality of life of respondents. We used data from questionnaires administered to randomly selected women and men living in the surveyed households [28–34].

2.6. Outcome Variable

The outcome variable, overall life satisfaction, was measured using the Satisfaction with Life Scale (SWLS), a versatile tool that can be adapted to different cultural contexts. Initially developed by Diener et al. [52], this five-item instrument asks respondents to rate aspects of their lifestyle on a scale from 1 ("very dissatisfied") to 5 ("very satisfied"), with total scores calculated as recommended by Diener et al. [53]. Building on this, Deaton [54] proposed a further evaluation by integrating Cantril's Self-Anchoring Ladder of Life Satisfaction [55] alongside Gallup's measure [56]; in this approach, respondents indicate their life satisfaction on a scale ranging from 0 to 10. This modification was designed to provide a more nuanced exploration of life satisfaction determinants while accounting for various psychodemographic factors. Thus, during the survey, participants reported their satisfaction level and position on the ladder. Following the adoption of Cantril's and Gallup's scales, the SWLS scores were reclassified into three categories: "Thriving" for scores 7–10 (coded as 2), "Struggling" for scores 5–6 (coded as 1), and "Suffering" for scores 0–4 (coded as 0). This recording enhances interpretability and offers a clearer picture of overall well-being in the South African context. Prior studies have

validated the SWLS with Cantril's Ladder and Gallup's approach as reliable and robust tools for assessing life satisfaction in various settings [56–60].

2.6.1. Operationalization of Life Satisfaction levels

The operational definitions of the levels of life satisfaction (suffering, struggling, and thriving) provide a clear understanding of the respondents' varying levels of life satisfaction and well-being, using Gallup [54] and Cantril's Self-Anchoring Ladder of Life Satisfaction measures [55]. Thus,

- **Suffering (0–4)**: Respondents in this category face significant challenges, including health, social, or economic issues. They often struggle to achieve satisfaction in their life, or maintain a stable quality of life, leading to poor overall well-being [52–55].
- **Struggling (5–6)**: Respondents in this category are moderately satisfied with their lives. Despite occasional health challenges, they manage life's obstacles, alternating between periods of stability and hardship [52–55].
- **Thriving (7–10)**: Respondents in this category experience high life satisfaction, well-being, and optimism. Access to resources and support networks enhances their overall happiness and contentment [52–55].

2.7. Explanatory Variables

The explanatory variables in the study were grouped into three distinct categories: individual, household, and community-level factors. Variables at the individual level included age, gender, educational attainment, demographic classification, income, and employment status; the number of persons living in the home, the number of individuals under the age of 18, child hunger, the SASSA social grant, the health facility, and medical aid were factors at the household level. Dwelling type, media accessibility, municipalities, and survey years are examples of community-level characteristics [28–34]. The availability of the variables and earlier research findings served as the basis for selecting these predictors [1,21]. Certain variables from the dataset retained their original categorization during this analysis, while others were meticulously redefined and recorded to enhance the study's precision [28–34]. Most selected variables were evaluated as straightforward binary measures, requiring "yes" or "no" responses. Additionally, some variables were derived by aggregating answers from multiple questions. Comprehensive descriptions of these variables are provided in another section [28–34] (Refer to Table 1 below).

Table 1. Detailed explanations of individual-level, household-level and community-level variables categorization

**Individual-level factors**	**Categorization**
Age	1 = 18–27; 2 = 28–37; 3 = 38–47; 4 = 48+ years
Sex	1 = male; 2 = female
Highest education	1 = no education; 2 = primary; 3 = secondary; 4 = higher
Population group	1 = Black African; 2 = Coloured; 3 = White; 4. Indian/Asian
Respondent's income	1 = no income; 2 = low (R1–R12,800); 3 = middle (R12,801–R25,600); 4 = high (R25,601+)
Employment	1 = not working; 2 = working
Medical aid	1 = no; 2 = yes
Healthcare facility	1 = public; 2 = private; 3 = both public and private; 4 = traditional
Life satisfaction	0 = suffering; 1 = struggling; 2 = thriving
**Household-level factors**	
Number of people living in H/H	1 = 1; 2 = 2; 3 = 3; 4 = 4+ persons
Number of people under 18 in H/H	1 = 0; 2 = 1; 3 = 2; 4 = 3; 5 = 4+ persons
Not enough to feed children	1 = No; 2 = Yes; 3 = No children in H/H
Receiving SASSA social grant	1 = No; 2 = Yes
**Community-level factor**	
Type of dwelling	1 = Formal; 2 = Informal

Basic services	1 = No; 2 = Yes
Municipality	1 = City of Ekurhuleni; 2 = City of Johannesburg; 3 = City of Tshwane; 4 = Emfuleni; 5 = Lesedi; 6 = Midvaal; 7 = Merafong; 8 = Mogale City; 9 = Rand West
Survey years	1 = 2009; 2 = 2011; 3 = 2013–2014; 4 = 2015–2016; 5 = 2017–2018; 6 = 2020–2021; 7 = 2023–2024

Source: Authors' compilation, 2024.

2.8. How Questions Derived from the 2009–2024 GCRO QoL Questionnaire Instrument Will Be Analyzed

Questions were retrieved from the Quality of Life I (2009–2020) and Quality of Life 7 (2023/2024) Survey: Full Questionnaire. The questions from the GCRO QoL instrument will be analyzed as presented in Table 2.

Table 2. Distribution of variables showing the instrument variable names and how they were analyzed.

S/N	Variable Description	Variable Name	Variable Analysis
1	What is the sex of the respondent?	a2_sex	Categorization of variables and the use of descriptive statistics for analysis
2	How old are you?	q14_2_age	Categorization of variables and the use of summary and descriptive statistics for analysis
3	To which population group does the respondent belong?	a1_pop_group	Categorization of variables and the use of summary and descriptive statistics for analysis
4	What is the highest level of education you have completed?	q14_1_education	Categorization of variables and the use of summary and descriptive statistics for analysis
5	What is the total amount of money brought into the household per month by all household members?<br><br>Please include all the money coming into the household, from all sources. This includes grants, help from friends and family, and rental or business income. Please exclude amounts deducted for tax, medical aid and pension contributions.	q15_3_income	Categorization of variables and the use of summary and descriptive statistics for analysis
6	In the past 7 days, did you do any type of work, business, or activity for which you got paid or expected to be paid (even if just for one hour)? This includes selling things, piece work, and work that's part of your own business.	q10_2_working	Categorisation of variables and the use of summary and descriptive statistics for analysis
7	Are you personally covered by any form of medical aid or other medical insurance?	q13_5_medical_aid	Categorisation of variables and the use of summary and descriptive statistics for analysis
8	Where do you usually go for health care?	q13_1_healthcare	Categorisation of variables and the use of summary and descriptive statistics for analysis
9	How many people, including you, live in this household? Please include babies and children.	q14_5_people	Categorisation of variables and the use of summary and descriptive statistics for analysis
10	How many members of this household are under 18 years old?	q14_6_under18	Categorisation of variables and the use of summary and descriptive statistics for analysis
11	In the past 12 months, has there ever been a time when there was not enough money to feed the children in the household?	q6_5_feed_children	Categorisation of variables and the use of summary and descriptive statistics for analysis
12	Does anybody in this household receive a social grant of any type, such as an old age pension, child care or disability grant?	q14_10_social_grant	Categorisation of variables and the use of summary and descriptive statistics for analysis
13	Which type of dwelling does this household occupy?	a3_dwelling_type	Categorisation of variables and the use of summary and descriptive statistics for analysis
14	Does this household have any of the following that are in good working order, that is not broken?	q6_3_1_landline; q6_3_2_cell; q6_3_3_tv; q6_3_4_computer; q6_3_5_radio; q6_3_6_dstv; q6_3_7_internet;	Categorisation of variables and the use of summary and descriptive statistics for analysis
15	Region/Municipality Name	municipality_coded	Categorisation of variables and the use of summary and descriptive statistics for analysis

16	How satisfied are you with your life AS A WHOLE these days?	q9_9_life	Bivariate and multivariate regression analysis will be employed
17	In which province or country were you born?	q3_1_birth_prov	Will be used to filter for South African nationals

Source: Authors' compilation, 2024.

2.9. Data Preparation and Analysis

STATA version 2021 statistical software was used for data management and analysis. The study examined the individual-level, household-level and community-level factors determining life satisfaction among South African nationals aged 18 years or above, residing in the Gauteng Province. The dataset was analyzed according to the study's objectives and specific measures. Weighting variables from each survey were utilized alongside the "svy command" to address sampling imbalances and account for over- and under-representation. This approach also accommodated the intricate survey design and enhanced the generalizability of the results. The findings derived from the 2009–2024 datasets were presented as follows: First, univariate analysis was carried out to examine the distribution and characteristics using the descriptive statistics to report the demographic characteristics of the respondents, percentage and frequency distributions, and visualizations (vertical bar chart, stacked bar chart, grouped/clustered bar chart) (specific objective 1) (Figure 1, Figure 2, Figure 3, and Table 3). Second, bivariate analysis using Chi-square test of associations was employed to test the associations between life satisfaction and demographic determinants stratified by sex using 5% level of significance threshold (specific objective 2) (Table 4). Third, a choropleth map was employed to visualize the datasets for the category of 'suffering life satisfaction' stratified by sex (male and female) across the municipalities in Gauteng province in South Africa. A brown colour gradient was used to represent the data values for the male respondents, and a green colour gradient was utilized to represent the data values for the female respondents. This was done to make it easy to see how life satisfaction variable changes across the different municipalities (specific objective 3) (Figure 4 and Figure 5). Fourth, pathway analysis (or pathway model), mediation analysis and generalized structural equation modelling (gSEM) were employed to analyze the specific objective 4. Mediation analysis was utilized to explain the effects of the explanatory variable on the outcome variable (Table 5), while the pathway model was used to identify the direct, and indirect effects of explanatory variables on the outcome variable (Figure 6 and Figure 7), and the generalized structural equation modelling (gSEM) was used to study the effects of the determinants (education, employment, income, and survey years) on life satisfaction as the outcome variable; the variables were either ordinal or binary in nature (Table 7 and Table 8) as well as the percentages of the mediation analysis (Table 9). Therefore, combining the pathway models with gSEM will create more robust and comprehensive models to study the direct and indirect effects influencing life satisfaction [61–63]. Further, nonlinear combinations of parameter estimates (nlcom) were generated using the nlcom command on STATA to generate direct and total effects between the outcome, mediator, and independent variables [64,65], and the gSEM and nlcom were both stratified by sex, with statistical significance of $\rho < 0.05$ signifying level of precision (specific objective 4).

2.10 Handling of Missing Data and Bootstrapping in Mediation Analysis

Missing data in this study were carefully assessed to maintain methodological rigor. Irrelevant variables were excluded, and only relevant ones retained for analysis. The extent of missingness in key variables was examined using descriptive statistics, including frequencies and percentages. To address potential biases, bootstrapping with 5,000 resamples was applied in the Generalized Structural Equation Modeling (GSEM) and mediation analyses, improving the stability and reliability of indirect effect estimates. Chi-square tests confirmed that missing data were randomly distributed and not systematically associated with demographic variables. Visual tools such as charts and choropleth maps were used to explore missing data patterns across respondent groups. These procedures ensured that the mediation analysis remained robust, replicable, and free from distortions, strengthening the credibility of the findings.

2.10. Ethics

The Gauteng City-Region Observatory (GCRO) obtained ethical clearance from the Human Research Ethics Committee (Non-Medical) at the University of the Witwatersrand, Johannesburg (clearance number [H19/11/19]). Verbal consent was transparently obtained from participants aged 18 and above. All respondents were informed of the voluntary nature of participation and assured of confidentiality and anonymity throughout the study.

3. Results

Table 3 presents the weighted sample of 70,314 respondents stratified by sex (male: 33,325 [47.4%]; female: 36,989 [52.6%]) across survey years (2009–2024) in Gauteng province, South Africa. It also summarizes respondents' demographic characteristics, organized into three categories: individual-level, household-level, and community-level factors.

3.1. Percentage Distribution of Male and Female Respondents

The multiple bar chart shows the percentage distribution of respondents by sex across the survey years as shown in Figure 1. Across the survey years, the majority of the respondents who participated in the survey from 2009–2024 were female (Figure 1).

3.2. Percentage Distribution of Life Satisfaction by Sex

Figure 2 illustrates the distribution of life satisfaction scales, categorized by sex, among individuals residing in Gauteng province, South Africa. The grouped bar chart revealed slight differences in respondents' reported life satisfaction between males (63.5%) and females (63.3%) (Figure 2).

3.3. Percentage Distribution of Respondents' Age by Life Satisfaction

Figure 3 shows the proportion by age indicating the life satisfaction scale among South Africans residing in Gauteng province across the survey years. From the stacked bar graph, struggling and suffering life satisfaction scales were mostly reported among age groups 18–27 years and 38–47 years. However, thriving life satisfaction level were majorly reported by age groups 28–37 years and 48+ years (Figure 3).

3.4. Demographic Characteristics of the Respondents

For the individual-level, a majority of both male (29.3%) and female (32.6%) respondents were aged 48 years above, and more respondents were found to be educated to the secondary level (males—65.5% and females—66.1%). 83% of both male and female respondents were Black African (Table 3). Moreover, more than half of the respondents reported earning low income (male—74.3%; female - 79.6%) and were not employed (males—56.7%; females—70.4%). By household factors, male (40.7%) and female (53.6%) respondents have 4 and more household members, and more reported that they have no children under 18 (male—53.4%; female—32%). Also, a majority of the respondents reported having no medical aid (males—83.5%; females—85.7%) and attend a private health facility (males—54.7%; females—59.6%). Furthermore, when considering community-level factors, most respondents indicated that they lived in a formal dwelling place (males—83.8%; females—85.2%), have access to media (males—98.7%; females—99.0%), and are resident in major municipalities of Gauteng—City of Ekurhuleni: male (26.8%); female (26.2%); City of Johannesburg: male (35.2%) and female (34.2%); and City of Tshwane: male (24.1%) and female (24.2%) (Table 3).

Table 3. Socio-demographic Characteristics of Respondents stratified by Sex (n = 70,314).

Demographics	Male (n = 33,325)		Female (n = 36,989)		Total (N = 70,314)	
**Individual-level factors**						
**Life satisfaction**	**Frequency**	**%**	**Frequency**	**%**	**Frequency**	**%**
Suffering	7826	23.5	8813	23.8	16639	23.7
Struggling	4342	13.0	4779	12.9	9121	13.0
Thriving	21157	63.5	23397	63.3	44554	63.4
**Age group**						
18-27	7806	23.4	8046	21.8	15852	22.5
28-37	8742	26.2	9422	25.5	18164	25.8
38-47	7022	21.1	7453	20.1	14475	20.6
48+	9755	29.3	12068	32.6	21824	31.0
**Highest education**						
No education	557	1.7	880	2.4	1437	2.0
Primary	3223	9.7	4377	11.8	7600	10.8
Secondary	21826	65.5	24444	66.1	46270	65.8
Higher	7719	23.2	7288	19.7	15007	21.3
**Pop group**						
Black African	27670	83.0	30692	83.0	58362	83.0
Coloured	1033	3.1	1256	3.4	2289	3.3
Indian/Asian	762	2.3	820	2.2	1581	2.2
White	3860	11.6	4221	11.4	8082	11.5
**Income**						
No income	2203	6.6	1834	5.0	4037	5.7
Low	24762	74.3	29448	79.6	54210	77.1
Middle	3456	10.4	3257	8.8	6713	9.5
High	2904	8.7	2450	6.6	5354	7.6
**Employment**						
Not employed	18893	56.7	26044	70.4	44937	63.9
Employed	14432	43.3	10945	29.6	25377	36.1
**Household-level factors**						
**HH members**						
1	8111	24.3	3557	9.6	11668	16.6
2	6308	18.9	6542	17.7	12850	18.3
3	5357	16.1	7078	19.1	12435	17.7
4 and above	13549	40.7	19812	53.6	33361	47.4
**Children under 18 in HH**						
None	17802	53.4	11846	32.0	29648	42.2
1	5746	17.2	8094	21.9	13840	19.7
2	5256	15.8	8466	22.9	13722	19.5
3	3224	9.7	5525	14.9	8749	12.4
4+	1297	3.9	3058	8.3	4355	6.2
**Child hunger**						
No	19279	57.9	24312	65.7	43591	62.0
Yes	3811	11.4	6506	17.6	10317	14.7
No children in HH	10235	30.7	6171	16.7	16406	23.3
**Social Grant**						
No	20187	60.6	15257	41.2	35444	50.4
Yes	13138	39.4	21732	58.8	34870	49.6
**Health facility**						
Public	11429	34.3	11343	30.7	22772	32.4
Private	18236	54.7	22062	59.6	40298	57.3
Both public and private	3216	9.6	3369	9.1	6585	9.4
Traditional	444	1.3	215	0.6	659	0.9

**Medical aid**						
No	27839	83.5	31683	85.7	59522	84.7
Yes	5486	16.5	5306	14.3	10792	15.3
**Community-level factors**						
**Dwelling type**						
Formal	27931	83.8	31510	85.2	59441	84.5
Informal	5394	16.2	5479	14.8	10873	15.5
**Access to media**						
No	426	1.3	380	1.0	806	1.1
Yes	32899	98.7	36609	99.0	69508	98.9
**Municipality**						
City of Ekurhuleni	8927	26.8	9692	26.2	18619	26.5
City of Johannesburg	11727	35.2	12870	34.8	24597	35.0
City of Tshwane	8024	24.1	8956	24.2	16980	24.1
Emfuleni	1958	5.9	2344	6.3	4302	6.1
Lesedi	271	0.8	324	0.9	595	0.8
Midvaal	264	0.8	245	0.7	509	0.7
Merafong	502	1.5	619	1.7	1121	1.6
Mogale City	990	3.0	1163	3.1	2153	3.1
Rand West	662	2.0	776	2.1	1438	2.0
**Survey year**						
2009	1888	5.7	2907	7.9	4795	6.8
2011	906	2.7	1381	3.7	2287	3.3
2013-2014	8044	24.1	8769	23.7	16813	23.9
2015-2016	8136	24.4	8401	22.7	16537	23.5
2017-2018	6211	18.6	6846	18.5	13057	18.6
2020-2021	4036	12.1	4421	12.0	8457	12.0
2023-2024	4104	12.3	4264	11.5	8368	11.9
**Total**	33325	100	36989	100	70314	100

3.5. Respondent's Life Satisfaction and Associated Factors (Individual-, Household-, and Community-Levels)

Table 4 presents the association between life satisfaction and factors-related indicators (individual-level, household-level, and community-level), stratified by sex using Chi-square. The results showed that there is a significant association between life satisfaction levels and individual-level factors (age group, education, population group, income, and employment) (p < 0 001) (Table 4). Regarding household factors, the Chi-square findings revealed that there is a significant association between life satisfaction levels and household-level factors (HH members, children under 18 in HH, child hunger, social grant, health facility, and medical aid) (p < 0 001) (Table 4). Further, concerning community-level factors, the Chi-square results showed that there is a significant association between life satisfaction levels and community-level factors (dwelling type, access to media, municipality, and survey year) (p < 0 001) (Table 4).

Table 4. *Chi-square analysis showing the associations between life satisfaction and factor-related indicators (individual, household, and community) among male and female respondents (n = 70,314).*

	Males								Females							
**Individual-level factors**	**Suffering**		**Struggling**		**Thriving**				**Suffering**		**Struggling**		**Thriving**			
**Age group**	**Frequency**	**%**	**Frequency**	**%**	**Frequency**	**%**	**X^2**	**p-value**	**Frequency**	**%**	**Frequency**	**%**	**Frequency**	**%**	**X^2**	**p-value**
18-27	2,020	25.9	1,096	14.0	4,690	60.1	248.519	0.000	2,170	27.0	1,098	13.6	4,777	59.4	412.362	0.000
28-37	2,364	27.0	1,228	14.0	5,150	58.9			2,578	27.4	1,282	13.6	5,561	59.0		
38-47	1,651	23.5	945	13.5	4,427	63.0			1,862	25.0	945	12.7	4,646	62.3		
48+	1,791	18.4	1,073	11.0	6,891	70.6			2,203	18.3	1,454	12.0	8,412	69.7		
**Highest education**																
No education	166	29.9	62	11.1	329	59.1	637.891	0.000	270	30.6	113	12.8	498	56.5	445.155	0.000
Primary	871	27.0	416	12.9	1,937	60.1			1,070	24.4	570	13.0	2,738	62.5		
Secondary	5,743	26.3	3,009	13.8	13,074	59.9			6,392	26.1	3,239	13.3	14,813	60.6		
Higher	1,046	13.6	855	11.1	5,817	75.4			1,082	14.8	858	11.8	5,349	73.4		
**Population group**																
Black African	7,298	26.4	3,791	13.7	16,580	59.9	890.601	0.000	8,232	26.8	4,177	13.6	18,284	59.6	1,000.000	0.000
Coloured	192	18.6	128	12.4	713	69.0			246	19.6	176	14.0	833	66.4		
Indian/Asian	92	12.1	95	12.5	574	75.4			94	11.5	80	9.8	645	78.7		
White	244	6.3	327	8.5	3,289	85.2			240	5.7	347	8.2	3,634	86.1		
**Income**																
No income	807	36.6	274	12.5	1,122	50.9	1,200.000	0.000	579	31.6	240	13.1	1,015	55.4	1,100.000	0.000
Low	6,449	26.0	3,451	13.9	14,862	60.0			7,744	26.3	4,011	13.6	17,693	60.1		
Middle	368	10.6	395	11.4	2,693	77.9			342	10.5	331	10.2	2,583	79.3		
High	203	7.0	221	7.6	2,480	85.4			147	6.0	197	8.1	2,105	85.9		
**Employment**																
Not employed	4,905	26.0	2,444	12.9	11,543	61.1	136.461	0.000	6,558	25.2	3,304	12.7	16,182	62.1	95.314	0.000
Employed	2,921	20.2	1,898	13.2	9,613	66.6			2,255	20.6	1,475	13.5	7,215	65.9		
**Household-level factors**																
**HH members**																
1	2,117	26.1	1,099	13.5	4,895	60.4	70.421	0.000	757	21.3	487	13.7	2,313	65.0	98.794	0.000

2	1,365	21.6	785	12.4	4,158	65.9			1,289	19.7	807	12.3	4,446	68.0		
3	1,119	20.9	677	12.6	3,560	66.5			1,599	22.6	925	13.1	4,554	64.3		
4 and above	3,225	23.8	1,781	13.1	8,544	63.1			5,168	26.1	2,561	12.9	12,084	61.0		
**Children under 18 in HH**																
None	4,135	23.2	2,305	12.9	11,361	63.8	48.661	0.000	2,349	19.8	1,480	12.5	8,018	67.7	252.620	0.000
1	1,299	22.6	755	13.1	3,693	64.3			1,855	22.9	1,065	13.2	5,174	63.9		
2	1,186	22.6	665	12.7	3,405	64.8			2,110	24.9	1,091	12.9	5,264	62.2		
3	815	25.3	453	14.0	1,956	60.7			1,499	27.1	777	14.1	3,249	58.8		
4+	392	30.2	164	12.6	742	57.2			1,001	32.7	366	12.0	1,691	55.3		
**Child hunger**																
No	3,848	20.0	2,603	13.5	12,828	66.5	698.616	0.000	5,058	20.8	3,266	13.4	15,988	65.8	1,400.000	0.000
Yes	1,563	41.0	496	13.0	1,752	46.0			2,661	40.9	844	13.0	3,001	46.1		
No children in HH	2,416	23.6	1,242	12.1	6,577	64.3			1,094	17.7	670	10.8	4,407	71.4		
**Social Grant**																
No	4,405	21.8	2,529	12.5	13,252	65.6	88.228	0.000	2,818	18.5	1,868	12.2	10,571	69.3	375.864	0.000
Yes	3,421	26.0	1,812	13.8	7,905	60.2			5,995	27.6	2,912	13.4	12,825	59.0		
**Health facility**																
Public	1,997	17.5	1,309	11.5	8,123	71.1	901.520	0.000	1,859	16.4	1,386	12.2	8,097	71.4	906.394	0.000
Private	5,342	29.3	2,497	13.7	10,397	57.0			6,470	29.3	2,858	13.0	12,734	57.7		
Both public and private	368	11.4	441	13.7	2,406	74.8			442	13.1	492	14.6	2,434	72.3		
Traditional	119	26.9	94	21.2	230	51.9			41	18.9	43	20.1	131	60.9		
**Medical aid**																
No	7,230	26.0	3,805	13.7	16,804	60.4	721.611	0.000	8,263	26.1	4,259	13.4	19,160	60.5	749.110	0.000
Yes	596	10.9	537	9.8	4,352	79.3			549	10.4	520	9.8	4,236	79.8		
**Community-level factors**																
**Dwelling type**																
Formal	5,674	20.3	3,569	12.8	18,687	66.9	1,000.000	0.000	6,563	20.8	4,016	12.7	20,931	66.4	1,400.000	0.000
Informal	2,152	39.9	772	14.3	2,469	45.8			2,250	41.1	764	13.9	2,466	45.0		
**Access to media**																

No	182	42.7	52	12.2	192	45.0	117.279	0.000	161	42.3	54	14.1	166	43.6	88.412	0.000
Yes	7,644	23.2	4,289	13.0	20,965	63.7			8,652	23.6	4,726	12.9	23,231	63.5		
**Municipality**																
City of Ekurhuleni	2,308	25.9	1,153	12.9	5,465	61.2	153.315	0.000	2,573	26.5	1,173	12.1	5,946	61.3	218.616	0.000
City of Johannesburg	2,551	21.8	1,643	14.0	7,533	64.2			2,909	22.6	1,842	14.3	8,120	63.1		
City of Tshwane	1,815	22.6	923	11.5	5,286	65.9			1,965	21.9	1,064	11.9	5,926	66.2		
Emfuleni	468	23.9	255	13.0	1,236	63.1			565	24.1	319	13.6	1,461	62.3		
Lesedi	49	18.0	29	10.8	193	71.2			63	19.3	41	12.6	220	68.1		
Midvaal	57	21.8	39	14.8	167	63.3			50	20.5	23	9.2	172	70.3		
Merafong	133	26.5	78	15.5	291	58.0			182	29.4	78	12.7	359	58.0		
Mogale City	257	26.0	147	14.9	586	59.2			289	24.9	155	13.3	718	61.8		
Rand West	188	28.4	75	11.3	399	60.3			216	27.9	85	11.0	475	61.2		
**Survey year**																
2009	575	30.4	405	21.4	909	48.1	543.243	0.000	1,105	38.0	531	18.3	1,271	43.7	984.975	0.000
2011	333	36.8	144	15.9	429	47.4			512	37.0	213	15.4	657	47.6		
2013-2014	1,835	22.8	794	9.9	5,416	67.3			1,926	22.0	815	9.3	6,028	68.7		
2015-2016	1,602	19.7	1,208	14.8	5,326	65.5			1,503	17.9	1,263	15.0	5,635	67.1		
2017-2018	1,407	22.7	713	11.5	4,091	65.9			1,530	22.4	843	12.3	4,474	65.3		
2020-2021	1,085	26.9	552	13.7	2,399	59.4			1,163	26.3	558	12.6	2,700	61.1		
2023-2024	990	24.1	527	12.8	2,587	63.0			1,074	25.2	557	13.1	2,633	61.7		

X^2 = Chi-square; Note ρ-value (ρ): *ρ < 0 001.

3.6. Choropleth Maps Illustrating the Spatial Distribution of Life Satisfaction (Suffering) Stratified by Sex in Gauteng Municipalities

Figure 4 and Figure 5 showed the spatial distribution of life satisfaction (suffering measured in high, moderate-high, moderate and low) by sex (males and females) across the different areas of the municipalities of Gauteng province using Choropleth maps. The gradient of colours on the maps showed that the darker shades indicate 'high suffering' and lighter shades indicate 'low suffering'.

3.6.1. Choropleth Maps Showing Distribution of Suffering Life Satisfaction among Males (n = 33,325)

Figure 4 below shows the proportion of suffering levels among male residents in municipalities of Gauteng province. The findings revealed that more males residing in Merafong city reported higher level of suffering (≥29.4), followed by those reporting moderate-high level of suffering residing in Rand West city and the city of Ekurhuleni municipalities (26.5–29.4). At the same time, males living in Mogale City and Emfuleni municipalities reported a moderate level of suffering (24.1–26.5). Also, the cities of Johannesburg, Midvaal, Lesedi, and the City of Tshwane reported a low level of suffering (≤24.1) (Figure 4).

3.6.2. Choropleth Maps Showing Distribution of Suffering Life Satisfaction among Females (n = 36,989)

Figure 5 below shows the proportion of suffering levels among female residents in municipalities of Gauteng province. The findings revealed that more females residing in Rand West city reported high suffering (≥28.4), while females residing in Mogale city, Merafong city, and the city of Ekurhuleni municipalities reported moderate-high suffering (25.9–28.4). Also, in Emfuleni and in the City of Tshwane municipalities, females reported moderate suffering (22.5–25.9). In the city of Johannesburg, Midvaal, and Lesedi municipalities, female respondents reported low suffering (≤22.6) (Figure 5).

3.7. Generalized Structural Equation Modelling (gSEM)

The Generalized Structural Equation Modelling (gSEM) presented the estimates of the model parameters to infer the underlying relationships of the direct, indirect, and total effects to understand the relationships between the explanatory variables and life satisfaction. Table 5 and Table 6 present the analysis of independent variables (with reference categories) and life satisfaction levels using gSEM, which comprehensively accounts for both direct and indirect effects.

3.7.1. GSEM showing the Determinants and Life Satisfaction among Male Respondents

Table 5 shows the analysis of the determinants (explanatory variables) and life satisfaction levels. As shown in Table 5, the findings revealed that demographic factors (education- higher: $\beta = 0.553$, SE = 0.181, 95% CI [0.199–0.908], $\varrho < 0.002$), employment- working: $\beta = -0.037$, SE = 0.017, 95% CI [-0.070–0.004], $\varrho < 0.030$), and income- low: $\beta = 0.436$, SE = 0.086, 95% CI [0.267–0.604], $\varrho < 0.000$; middle: $\beta = 1.009$, SE = 0.125, 95% CI [0.763–1.255], $\varrho < 0.000$); higher: $\beta = 0.953$, SE = 0.150, 95% CI [0.659–1.247], $\varrho < 0.000$) significantly predicted struggling life satisfaction levels (Table 5). Also for thriving life satisfaction, demographic factors such as education (higher: $\beta = 0.493$, SE = 0.122, 95% CI [0.254–0.732], $\varrho < 0.000$), employment (working: $\beta = 0.075$, SE = 0.035, 95% CI [0.006–0.143], $\varrho < 0.033$), income (low: $\beta = 0.480$, SE = 0.058, 95% CI [0.367–0.594], $\varrho < 0.000$; middle: $\beta = 1.462$, SE = 0.089, 95% CI [1.288–1.637], $\varrho < 0.000$; higher: $\beta = 1.875$, SE = 0.110, 95% CI [1.658–2.091], $\varrho<0.000$) significantly predicted thriving life satisfaction levels (Table 5).

Table 5. Showing the gSEM of the determinants of life satisfaction levels among male respondents (n = 33,325).

Males						
**Life Satisfaction (Outcome)**	**Coef. (base outcome)**	**Robust Std Err.**	**z**	**P>|Z|**	**[95% Conf. Interval]**	
**Struggling**						
No education	**Ref.**					
Primary	0.251	0.183	1.370	0.170	-0.107	0.609
Secondary	0.304	0.171	1.780	0.075	-0.031	0.638
Higher	0.553	0.181	3.060	0.002	0.199	0.908
Survey years	-0.037	0.017	-2.170	0.030	-0.070	-0.004
Not employed	**Ref.**					
Employed	0.140	0.050	2.780	0.005	0.041	0.239
No income	**Ref.**					
Low	0.436	0.086	5.070	0.000	0.267	0.604
Middle	1.009	0.125	8.050	0.000	0.763	1.255
Higher	0.953	0.150	6.350	0.000	0.659	1.247
_cons	-1.419	0.206	-6.890	0.000	-1.823	-1.015
**Thriving**						
No education	**Ref.**					
Primary	0.124	0.123	1.000	0.316	-0.118	0.365
Secondary	0.060	0.115	0.530	0.599	-0.165	0.285
Higher	0.493	0.122	4.040	0.000	0.254	0.732
Survey years	-0.007	0.011	-0.610	0.543	-0.029	0.015
Not employed	**Ref.**					
Employment	0.075	0.035	2.140	0.033	0.006	0.143
No income	**Ref.**					
Low	0.480	0.058	8.300	0.000	0.367	0.594
Middle	1.462	0.089	16.460	0.000	1.288	1.637
Higher	1.875	0.110	16.970	0.000	1.658	2.091
_cons	0.159	0.136	1.170	0.242	-0.108	0.425
**Survey years**						
No education	**Ref.**					
Primary	-0.062	0.092	-0.670	0.500	-0.243	0.118
Secondary	0.104	0.086	1.220	0.224	-0.064	0.273
Higher	0.138	0.089	1.560	0.120	-0.036	0.313
Not employed	**Ref.**					
Employment	-0.029	0.025	-1.170	0.243	-0.078	0.020
No income	**Ref.**					
Low	0.783	0.033	23.660	0.000	0.718	0.847
Middle	0.945	0.048	19.610	0.000	0.851	1.040
Higher	1.028	0.054	19.170	0.000	0.923	1.133
_cons	3.510	0.093	37.810	0.000	3.328	3.692
var(e.year)	2.476	0.021			2.435	2.517

Note: * p < 0 05, ** p < 0 01, *** p < 0 001.

3.7.2. GSEM showing the Determinants and Life Satisfaction among Female Respondents

Table 6 shows the analysis of the determinants (explanatory variables) and life satisfaction levels. As shown in Table 6, the findings revealed that demographic factors (education- higher: β = 0.396, SE = 0.129, 95% CI [0.144–0.648], p < 0.002), employment- employed: β = −0.156, SE = 0.045, 95% CI [0.067–0.245], p < 0.001), and income- low: β = 0.175, SE = 0.081, 95% CI [0.016–0.334], p < 0.031; middle: β = 0.664, SE = 0.123, 95% CI [0.422–0.906], p < 0.000); higher: β = 0.932, SE = 0.155, 95% CI [0.628–1.236], p < 0.000) predicted struggling life satisfaction levels (Table 6). Also, for thriving life satisfaction, the findings showed that demographic factors such as education (primary: β = 0.309, SE = 0.086, 95% CI [0.140–

0.477], ϱ < 0.000; higher: β = 0.456, SE = 0.088, 95% CI [0.284–0.629], p < 0.000), and income (low: β = 0.221, SE = 0.057, 95% CI [0.109–0.332], p < 0.000; middle: β = 1.295, SE = 0.089, 95% CI [1.121–1.469], p < 0.000; high: β = 1.872, SE = 0.118, 95% CI [1.640–2.103], p < 0.000) significantly predicted thriving life satisfaction levels (Table 6).

Table 6. Showing the gSEM of the determinants of life satisfaction levels among female respondents (n = 36,989).

		Females				
**Life Satisfaction (Outcome)**	**Coef. (base outcome)**	**Robust Std. Err.**	**z**	**p >|Z|**	**[95% Conf. Interval]**	
**Struggling**						
No education	**Ref.**					
Primary	0.224	0.127	1.760	0.078	-0.025	0.472
Secondary	0.139	0.117	1.180	0.238	-0.091	0.368
Higher	0.396	0.129	3.080	0.002	0.144	0.648
Survey years	0.017	0.014	1.210	0.228	-0.010	0.044
Not employed	**Ref.**					
Employed	0.156	0.045	3.440	0.001	0.067	0.245
No income	**Ref.**					
Low	0.175	0.081	2.160	0.031	0.016	0.334
Middle	0.664	0.123	5.380	0.000	0.422	0.906
Higher	0.932	0.155	6.010	0.000	0.628	1.236
_cons	-1.275	0.152	-8.410	0.000	-1.572	-0.978
**Thriving**						
No education	**Ref.**					
Primary	0.309	0.086	3.600	0.000	0.140	0.477
Secondary	0.131	0.079	1.660	0.096	-0.023	0.285
Higher	0.456	0.088	5.180	0.000	0.284	0.629
Survey years	0.047	0.010	4.740	0.000	0.028	0.066
Not employed	**Ref.**					
Employment	0.011	0.033	0.340	0.733	-0.054	0.077
No income	**Ref.**					
Low	0.221	0.057	3.880	0.000	0.109	0.332
Middle	1.295	0.089	14.590	0.000	1.121	1.469
Higher	1.872	0.118	15.850	0.000	1.640	2.103
_cons	0.205	0.105	1.950	0.052	-0.001	0.412
**Survey years**						
No education	**Ref.**					
Primary	0.118	0.068	1.730	0.083	-0.015	0.251
Secondary	0.354	0.062	5.670	0.000	0.231	0.476
Higher	0.537	0.067	8.030	0.000	0.406	0.668
Not employed	**Ref.**					
Employment	-0.096	0.025	-3.860	0.000	-0.145	-0.047
No income	**Ref.**					
Low	0.672	0.034	20.040	0.000	0.606	0.737
Middle	0.821	0.052	15.870	0.000	0.719	0.922
Higher	0.985	0.056	17.560	0.000	0.875	1.095
_cons	3.321	0.072	45.940	0.000	3.179	3.463
var(e.year)	2.692	0.020			2.653	2.731

Note: * p < 0 05, ** p < 0 01, *** p < 0 001.

3.8. Pathway Model (Pathway Analysis) of the Structural Equation Modelling (SEM)

The pathway model of the structural equation modelling (SEM) showed the impact of selected determinants (education, employment, income) on life satisfaction, with survey years, to estimate the direct and indirect paths (Figure 6 and Figure 7).

3.8.1. Pathway Model of the Determinants influencing Life Satisfaction among Male Respondents

Figure 6 shows the pathway model to illustrate the relationship between the independent variables (demographic factors and year) and the dependent variables (life satisfaction) by sex (males) (Figure 6).

3.8.2. Pathway Model of the Determinants influencing Life Satisfaction among Female Respondents

Figure 7 below shows the pathway model to illustrate the relationship between the independent variables (demographic factors and year) and the dependent variables (life satisfaction) by sex (females) (Figure 7).

3.9. Generalized Structural Modelling (gSEM) Analyses for Male Respondents (n = 33,325)

Table 7 and table 8 involves the generalized structural equation modelling (gSEM), which indicated the strength and direction of the relationships between the determinants and life satisfaction and the effects they have on each other, as direct, indirect and total effects.

3.9.1. Generalized Structural Modelling (gSEM) Analyses for Male Respondents (n = 33,325)

Table 7 presents the effects of education, employment, and income on life satisfaction (suffering, struggling, thriving). Our results show that higher education, employment, and income significantly enhance life satisfaction ($p < 0.000$). Higher education directly influences life satisfaction, promoting struggling and thriving among males, while employment status notably increases life satisfaction for those struggling or thriving. Income levels (low, middle, high) also positively affect life satisfaction ($p < 0.000$). Indirectly, higher education shapes employment status, which in turn influences life satisfaction ($p < 0.000$). The combined direct and indirect effects highlight the comprehensive role of higher education, employment, and income in improving life satisfaction. Overall, employment status emerges as a key driver, directly boosting life satisfaction and amplifying the effects of education and income.

Table 7. Generalized structural equation modelling for the determinants of life satisfaction as a confirmed factor for the pooled samples of the male respondents (n = 33,325).

		Direct effects				Indirect effects				Total effects			
		Coef.	P>|Z|	[95% Conf. Interval]		Coef.	P>|Z|	[95% Conf. Interval]		Coef.	P>|Z|	[95% Conf. Interval]	
**Effect of highest education on life satisfaction via year of survey**													
Primary	Suffering	Ref.											
	Struggling	0.251	0.170	-0.107	0.609	0.002	0.520	-0.005	0.009	0.253	0.166	-0.105	0.611
	Thriving	0.124	0.316	-0.118	0.365	0.000	0.651	-0.001	0.002	0.124	0.314	-0.117	0.365
Secondary	Suffering	Ref.											
	Struggling	0.304	0.075	-0.031	0.638	-0.004	0.287	-0.011	0.003	0.300	0.079	-0.035	0.634
	Thriving	0.060	0.599	-0.165	0.285	-0.001	0.588	-0.003	0.002	0.060	0.604	-0.165	0.284
Higher	Suffering	Ref.											
	Struggling	0.553	0.002	0.199	0.908	-0.005	0.204	-0.013	0.003	0.548	0.002	0.193	0.903
	Thriving	0.493	0.000	0.254	0.732	-0.001	0.571	-0.004	0.002	0.492	0.000	0.253	0.731
**Effect of employment on life satisfaction via year of survey**													
Employed	Suffering	Ref.											

	Struggling	0.140	0.005	0.041	0.239	0.001	0.321	-0.001	0.003	0.141	0.005	0.043	0.240
	Thriving	0.075	0.033	0.006	0.143	0.000	0.594	-0.001	0.001	0.075	0.032	0.006	0.144
**Effect of income on life satisfaction via year of survey**													
Low	Suffering	Ref.											
	Struggling	0.436	0.000	0.267	0.604	-0.029	0.030	-0.055	-0.003	0.407	0.000	0.238	0.575
	Thriving	0.480	0.000	0.367	0.594	-0.005	0.543	-0.023	0.012	0.475	0.000	0.362	0.588
Middle	Suffering	Ref.											
	Struggling	1.009	0.000	0.763	1.255	-0.035	0.031	-0.067	-0.003	0.974	0.000	0.729	1.219
	Thriving	1.462	0.000	1.288	1.637	-0.007	0.543	-0.028	0.015	1.456	0.000	1.282	1.629
High	Suffering	Ref.											
	Struggling	0.953	0.000	0.659	1.247	-0.038	0.031	-0.073	-0.003	0.915	0.000	0.622	1.207
	Thriving	1.875	0.000	1.658	2.091	-0.007	0.543	-0.030	0.016	1.868	0.000	1.652	2.083

Note: * p < 0 05, ** p < 0 01, *** p < 0 001.

3.9.2. Generalized Structural Modelling (gSEM) Analyses for Female Respondents (n = 36,989)

Table 8 presents the effects of demographic factors—education, employment, and income—on life satisfaction (suffering, struggling, and thriving) among female respondents. Significant positive total effects were observed for struggling and thriving life satisfaction across higher education, employment status, and income levels (p ≤ 0.000). Education at all levels (primary, secondary, higher) directly increased struggling and thriving life satisfaction, as did employment and income status (p ≤ 0.000). Indirectly, education influenced life satisfaction by improving employment status, which in turn raised income levels, both contributing to higher life satisfaction (p ≤ 0.000). The combined direct and indirect pathways highlight the comprehensive impact of education, employment, and income on life satisfaction among females.

Table 8. Generalized structural equation modelling for the determinants of life satisfaction as a confirmed factor for the pooled samples of the female respondents (n = 36,989).

Demographic Determinants		Direct effects				Indirect effects				Total effects			
		Coef.	P>|Z|	[95% Conf. Interval]		Coef.	P>|Z|	[95% Conf. Interval]		Coef.	P>|Z|	[95% Conf. Interval]	
**Effect of highest education on life satisfaction via year of survey**													
Primary	Suffering												
	Struggling	0.224	0.078	-0.025	0.472	0.002	0.321	-0.002	0.006	0.226	0.075	-0.023	0.474
	Thriving	0.309	0.000	0.140	0.477	0.006	0.105	-0.001	0.012	0.314	0.000	0.145	0.484
+ Secondary	Suffering												
	Struggling	0.139	0.238	-0.091	0.368	0.006	0.237	-0.004	0.016	0.144	0.219	-0.086	0.374
	Thriving	0.131	0.096	-0.023	0.285	0.017	0.000	0.008	0.026	0.148	0.062	-0.008	0.303
Higher	Suffering												
	Struggling	0.396	0.002	0.144	0.648	0.009	0.233	-0.006	0.024	0.405	0.002	0.153	0.657
	Thriving	0.456	0.000	0.284	0.629	0.025	0.000	0.013	0.037	0.482	0.000	0.308	0.655
**Effect of employment on life satisfaction via year of survey**													
Employed	Suffering												
	Struggling	0.156	0.001	0.067	0.245	-0.002	0.251	-0.004	0.001	0.155	0.001	0.066	0.243
	Thriving	0.011	0.733	-0.054	0.077	-0.005	0.003	-0.008	-0.002	0.007	0.836	-0.058	0.072
**Effect of income on life satisfaction via year of survey**													
Low	Suffering												
	Struggling	0.175	0.031	0.016	0.334	0.011	0.228	-0.007	0.029	0.186	0.022	0.027	0.345
	Thriving	0.221	0.000	0.109	0.332	0.032	0.000	0.018	0.045	0.252	0.000	0.140	0.364
Middle	Suffering												
	Struggling	0.664	0.000	0.422	0.906	0.014	0.229	-0.009	0.036	0.678	0.000	0.436	0.920
	Thriving	1.295	0.000	1.121	1.469	0.039	0.000	0.022	0.055	1.334	0.000	1.159	1.508
High	Suffering												
	Struggling	0.932	0.000	0.628	1.236	0.016	0.228	-0.010	0.043	0.948	0.000	0.645	1.251
	Thriving	1.872	0.000	1.640	2.103	0.046	0.000	0.026	0.066	1.918	0.000	1.688	2.148

Note: * ρ < 0 05, ** ρ < 0 01, *** ρ < 0 001.

3.9.3 Percentage distribution of GSEM mediation analysis of life satisfaction among male and female respondents

Table 9 below shows SEM mediation percentages by gender, highlighting differences in life satisfaction determinants in Gauteng. Income was the strongest predictor, with high-income individuals reporting the highest total effects for both sexes. Thriving high-income women had a total effect of 1.918 ($p < 0.001$) with 2.4% mediation; men had slightly higher effects with similar mediation. Education positively influenced life satisfaction, especially at higher levels. Among thriving women, 5.2% of higher education's effect was mediated by survey year, compared to a lower rate for men. Secondary education for thriving women showed the highest mediation (11.5%), reflecting sensitivity to historical and policy shifts; men showed lower, more stable mediation. Employment effects differed by gender: for struggling women, employment increased life satisfaction (0.155, $p = 0.001$) with negligible mediation (-1.3%), while men had stronger direct and slightly higher indirect effects mediated by survey year. Overall, mediation was low (< 5%) except for secondary education among thriving women, indicating socio-economic factors primarily drive life satisfaction with modest gendered mediation by temporal and policy changes.

3.9.4 Key Gendered Findings

Income is the strongest predictor of life satisfaction for both men and women, with only modest influence from historical or policy changes. Education positively affects life satisfaction across genders, but secondary education among women shows the greatest responsiveness to socio-political shifts. Employment enhances life satisfaction for both sexes, with more stable and slightly better mediation for men; women's gains are most evident among vulnerable groups, showing little policy-driven change over time. Overall, mediation effects remain small for most socio-economic factors, though gender differences appear in education and employment.

Table 9. GSEM Mediation Percentages for Life Satisfaction Determinants among Male (n = 33,325) and Female (n = 36,989) Respondents

Demographic determinants	Males						Females				
	Category	Direct effects	Indirect effects	Total effect	% Mediated	Significance (Total)	Direct effects	Indirect effects	Total effect	% Mediated	Significance (Total)
Education (primary)	Suffering	**RC**	**RC**	**RC**	**RC**	**RC**	**RC**	**RC**	**RC**	**RC**	**RC**
	Struggling	0.251	0.002	0.253	0.79%	0.166	0.224	0.002	0.226	0.88%	0.075
	Thriving	0.124	-0.001	0.124	-0.81%	0.314	0.309	0.006	0.314	1.91%	0.000
Education (secondary)	Suffering	**RC**	**RC**	**RC**	**RC**	**RC**	**RC**	**RC**	**RC**	**RC**	**RC**
	Struggling	0.304	-0.004	0.300	-1.33%	0.079	0.139	0.006	0.144	4.17%	0.219
	Thriving	0.060	-0.003	0.060	-5.00%	0.604	0.131	0.017	0.148	11.9%	0.062
Education (higher)	Suffering	**RC**	**RC**	**RC**	**RC**	**RC**	**RC**	**RC**	**RC**	**RC**	**RC**
	Struggling	0.553	-0.005	0.548	-0.91%	0.002	0.396	0.009	0.405	2.22%	0.002
	Thriving	0.493	-0.004	0.492	-0.81%	0.000	0.456	0.025	0.482	5.19%	0.000
Employment (employed)											
	Suffering	**RC**	**RC**	**RC**	**RC**	**RC**	**RC**	**RC**	**RC**	**RC**	**RC**
	Struggling	0.140	-0.001	0.141	-0.71%	0.005	0.156	-0.002	0.155	-1.29%	0.001
	Thriving	0.075	-0.001	0.075	-1.33%	0.032	0.011	-0.002	0.007	-28.57%	0.836
Income (low)											
	Suffering	**RC**	**RC**	**RC**	**RC**	**RC**	**RC**	**RC**	**RC**	**RC**	**RC**
	Struggling	0.436	0.030	0.407	7.37%	0.000	0.175	0.007	0.186	3.76%	0.022
	Thriving	0.480	0.012	0.475	2.53%	0.000	0.221	0.018	0.252	7.14%	0.000
Income (middle)	Suffering	**RC**	**RC**	**RC**	**RC**	**RC**	**RC**	**RC**	**RC**	**RC**	**RC**
	Struggling	1.009	0.035	0.974	3.59%	0.000	0.664	0.014	0.678	2.06%	0.000
	Thriving	1.462	0.015	1.456	1.03%	0.000	1.295	0.039	1.334	2.92%	0.000
Income (higher)	Suffering	**RC**	**RC**	**RC**	**RC**	**RC**	**RC**	**RC**	**RC**	**RC**	**RC**
	Struggling	0.953	0.031	0.915	3.39%	0.000	0.932	0.016	0.948	1.69%	0.000
	Thriving	1.875	0.016	1.868	0.86%	0.000	1.872	0.066	1.918	3.44%	0.000

Note: * p < 0 05, ** p < 0 01, *** p < 0 001.

4. Discussion

This study identified key determinants of life satisfaction among adults in Gauteng, South Africa, highlighting the influence of age, education, income, employment, household size, medical aid, and media access. While gender differences were modest, women generally reported higher satisfaction levels, shaped by cultural and socioeconomic factors. The findings support the U-curve hypothesis, with life satisfaction lowest in midlife and higher in early adulthood and after 48 years. Regional variations persist, as life satisfaction declines with age in many low- and middle-income contexts due to economic and social instability. These findings underscore the importance of addressing employment, education, and health access while considering gendered and age-specific needs to improve life satisfaction outcomes.

Career fulfilment, job satisfaction, financial stability, and concerns about retirement and debt significantly shape life satisfaction in middle age [70]. Supportive family and social relationships improve satisfaction, while conflict and poor work-life balance reduce it [76,79,80]. Personal development opportunities and a sense of purpose further enhance well-being [79,80]. For older adults, life satisfaction depends on health, financial security, social ties, living conditions, and healthcare access, with good health and economic stability promoting satisfaction and chronic illness or financial strain diminishing it [77,81]. A strong sense of community and accessible healthcare are also vital [77]. This study found significant associations between individual-, household-, and community-level factors and life satisfaction, underscoring their interconnected roles. Positive links between personal achievement, household stability, and community resources reinforce the importance of social cohesion. These findings support integrated policies addressing inequality, promoting equity, and advancing sustainable development goals [1,78]. Interventions targeting these key factors can meaningfully enhance life satisfaction across age groups.

Younger and older adults report higher life satisfaction than middle-aged individuals, reflecting differences in life stages, responsibilities, and stress levels [1,82]. Higher education improves life satisfaction by enhancing job prospects, income, and problem-solving abilities [82,83]. Cultural background and social identity also shape satisfaction through differing expectations and support systems [1,2]. Although higher income improves life satisfaction, its impact decreases once basic financial needs are met and access to essential resources is secured [1,24]. Employment contributes more to life satisfaction than unemployment by providing financial security, purpose, and social interaction [1,17]. In South Africa, key drivers of life satisfaction include higher income, stable employment, good health, education, supportive relationships, and low fear of crime [1,24]. Smaller households report greater satisfaction owing to reduced financial strain [84,85], and medical insurance is strongly associated with well-being [86,87]. Higher household income improves living conditions, healthcare, and education opportunities [57,87], while social grants help ease financial burdens in low-income households [1,24]. Access to media enhances life satisfaction by offering information, entertainment, and social connection [88,89]. Lastly, stable, quality housing contributes to security and life satisfaction, whereas poor housing diminishes it [1,90,91].

Community-level factors such as safety, housing quality, media access, and public amenities play a crucial role in shaping life satisfaction for both men and women in South Africa [88–91]. Access to media fosters connection and reduces isolation, while safe, well-maintained housing offers stability and improves psychological well-being. Communities with strong social support networks, clean environments, and accessible facilities report higher life satisfaction [1,92]. In Gauteng, urban municipalities report greater life satisfaction due to better infrastructure, healthcare, education, and employment opportunities, while rural areas experience lower satisfaction due to limited services and resources [1,24]. These findings underscore the importance of physical living conditions, community infrastructure, and social cohesion in promoting life satisfaction across diverse settings. Employment opportunities and safety perceptions impact men's and women's life satisfaction differently. Urban areas offer diverse jobs, boosting life satisfaction, while rural areas have

limited, gender-specific employment [1,24]. Higher urban crime rates particularly affect women's safety and satisfaction [92]. Stronger community ties and traditional gender roles in rural areas influence life satisfaction differently by gender [93–95]. Tailored policies addressing the distinct needs of men and women in urban and rural settings—improving infrastructure, services, and safety—can enhance overall life satisfaction.

This study used generalized structural equation modeling (gSEM) to examine how education, employment, and income mediate the relationship between sex and life satisfaction (struggling and thriving). For males, primary to higher education, employment, and income significantly influenced struggling life satisfaction, with higher education, employment, and income boosting thriving life satisfaction consistently across survey years [96–102]. Stable, well-paying jobs and job security were linked to better male life satisfaction. Similarly, these determinants influenced female life satisfaction throughout the survey period, with higher education and income promoting thriving life satisfaction [99,100,103–122]. Education enhances job opportunities, income, and personal achievement, while income reduces stress and offers fulfilling experiences [1,57,89,111]. Employment provides purpose and financial independence, with job quality and work-life balance being key [89,112]. Women's physical and mental health, along with strong social networks, are crucial for life satisfaction, offering emotional support and belonging [69,99,103,110]. These findings align with prior research showing that demographic factors foster life satisfaction for both sexes, moderated by individual and cultural differences [1,24].

Female-headed households often face lower quality of life and greater economic vulnerability than male-headed ones, partly due to commuting challenges that limit employment access [89–94, 97,101]. These households rely more on social grants, reflecting gender disparities in urbanization and intersecting with race and ethnicity to affect resource access and well-being [90,91,94]. Addressing these inequalities requires targeted policies to support female-headed households—often single-parent or skipped-generation families—by enhancing earning capacities and reducing grant dependency [90,98,103,106]. Mapping data from the GCRO (2009–2024) can guide transportation and urban planning to improve accessibility, especially for women, informing strategies to promote gender equality and service access in Gauteng [28–34, 98,103]. Income remains the strongest determinant of life satisfaction for both genders, with limited mediation over time, highlighting persistent socio-economic inequalities shaping well-being in South Africa.

Education's impact on life satisfaction shows clear gender differences. While higher education improves life satisfaction for both sexes, women—especially with secondary education—are more affected by policy and temporal changes, indicating middle-tier education as a key intervention point. Employment effects also differ as men experience more stable and mediated benefits over time, whereas women, particularly those struggling, see direct but less mediated gains. This reflects persistent structural barriers in the labour market for women, where employment alone may not improve quality of life without gender-responsive policies. These findings call for gender-sensitive approaches that address the evolving and complex socio-economic inequalities beyond income and education.

4.1. Further Discussion: Implementation Science for Gender-Sensitive Psychodemographic and Sociology Practices

Gender sensitivity promotes respect across sexes, helping to avoid stereotypes and benefit all [113,114]. Gender-transformative interventions challenge harmful norms, especially around masculinity, and are vital to addressing complex inequalities in resources, power, and opportunities [115]. Effective gender-sensitive policies must address specific needs to promote equality and life satisfaction. Implementation science plays a crucial role by translating research into practice, identifying effective interventions, overcoming barriers, and enabling continuous monitoring and adjustment [112,115–117]. Engaging stakeholders ensures relevance and acceptance, supporting sustainable, impactful gender equality initiatives [101,118].

South Africa's gender equality efforts, including the National Policy Framework and UN Women's strategy [104,108], seek to close gender gaps, but disparities persist. Gender-sensitive policies that ensure equal access to resources, opportunities, and protections can improve life satisfaction and promote equity [114,115]. Implementation science is essential to making these policies effective and advancing outcomes for all genders [115–118]. Raising awareness and promoting gender equality further enhances life expectations and social well-being.

4.2. Strengths and Limitations

This study's key strength lies in its gender-specific analysis of life satisfaction determinants within Gauteng's social context, providing nuanced insights into male and female experiences. Utilizing the provincially representative GCRO QoL dataset (2009–2024) enhances reliability and generalizability while minimizing self-report biases. Integrating perspectives from sociology, psychology, and demography further deepens understanding of how individual, household, and community factors shape life satisfaction and highlight areas for improvement. However, the cross-sectional design limits causal inference and trend analysis. Future research should adopt longitudinal approaches and advanced methods like multilevel modeling to better capture gender-specific variations over time. The omission of variables such as marital status and religion—due to non-response—may affect model accuracy, so including these in future surveys is recommended. Overall, this study expands the literature on life satisfaction in Gauteng, offering a comprehensive foundation for future gender-focused research.

4.3. Implications for Sociology of Life Satisfaction and Pyschodemographic Research

Our research shows that social factors mediate the relationship between demographic determinants and life satisfaction. Understanding these determinants aids in creating targeted interventions to improve life satisfaction in South Africa. Gender equality plays a crucial role by challenging traditional roles, improving resource access, and promoting fairness, which correlates with higher life satisfaction for both men and women [70,110]. Gender equality empowers women's decision-making and reduces pressures on men to conform, fostering diverse interests and careers. Psycho-demographic and sociological research should guide gender-sensitive interventions that address the distinct needs of both sexes. Implementing evidence-based policies and programs based on these insights can build a more equitable society where everyone can achieve greater well-being.

5. Conclusions

Our study examined how demographic determinants and gender influence life satisfaction among men and women. We found strong links between education, employment, income, and gendered experiences of struggling or thriving life satisfaction. Gender moderated the association between municipalities and suffering-related life satisfaction, reflecting how lived experiences and social roles vary by location. Including gender in life satisfaction research is vital, as it shapes career, family, and identity outcomes. Promoting gender equality and challenging stereotypes fosters a society with equal rights and opportunities for all [100,101]. Gender-responsive programs enhance well-being across genders [100,101]. Although socio-economic factors directly affect life satisfaction in Gauteng, the impact of historical and policy changes remains modest, especially for women. These results highlight the need for targeted, gender-sensitive policies that address structural inequalities and strengthen public policy's mediating role. Based on these findings, we propose three key recommendations for South Africa:

Research and Data Collection

The Gauteng City Region Observatory (GCRO) should integrate variables from sociology, psychology, and psychodemographics into its surveys to enable intersectional analyses of gendered life satisfaction and well-being. This will enhance the understanding of how gender intersects with socio-economic and spatial inequalities, informing more targeted, inclusive policy responses.

Targeted Intervention and Programme Initiatives

Municipalities facing economic and social decline need inclusive economic empowerment initiatives for both women and men. Concurrently, public sensitisation campaigns on gender roles and stereotypes should promote equality, enabling individuals to pursue personal and professional goals free from traditional constraints.

Policy Advocacy

Provinces must adopt comprehensive, gender-responsive policy reforms addressing systemic inequalities. Strengthening and funding gender advocacy networks is essential to ensure sustained support for the diverse needs of women and men and to advance equitable socio-economic development.

Author Contributions: Conceptualization: Monica Ewomazino Akokuwebe; Data curation: Monica Ewomazino Akokuwebe, Salmon Likoko, Shamsunisaa Miles-Timotheus; Formal analysis: Salmon Likoko, Monica Ewomazino Akokuwebe, Shamsunisaa Miles-Timotheus; Interpretation and critical review of results: Monica Ewomazino Akokuwebe; Funding acquisition: None; Investigation: Monica Ewomazino Akokuwebe; Methodology: Monica Ewomazino Akokuwebe, Salmon Likoko; Project administration: Monica Ewomazino Akokuwebe; Software: Salmon Likoko, Shamsunisaa Miles-Timotheus; Supervision: Monica Ewomazino Akokuwebe; Writing – Proposal draft, First original draft, Second original draft, Third original draft: Monica Ewomazino Akokuwebe; Writing – review & editing: Monica Ewomazino Akokuwebe, Salmon Likoko; Validation: Monica Ewomazino, Akokuwebe, Salmon Likoko, Shamsunisaa Miles-Timotheus, Moyahabo Mabala; Manuscript Revisions: Monica Ewomazino Akokuwebe, Salmon Likoko; Consent - All authors read and consented to submit the manuscript for peer review and published version of the manuscript.

Funding: The author(s) received no specific funding for this work.

Ethics Approval and Consent to Participate: The Human Research Ethics Committee (non-medical) at the University of the Witwatersrand, through the Johannesburg Research Office, granted ethical clearance to the GCRO organisation for the design, procedures, and survey instruments used in its regular Quality of Life (QoL) surveys. Additionally, the University of the Witwatersrand's Institutional Review Board (IRB) in Johannesburg reviewed the GCRO QoL survey methodology. Before taking part in the study, all human subjects provided their informed consent, according to the GCRO Field Staff. The Human Research Ethics Committee (non-medical), University of the Witwatersrand, Johannesburg, has examined and approved the protocols and questions for the standard GCRO QoL surveys. This study has no human volunteers; it only uses secondary data. Consequently, no official ethical approval was needed. However, to utilise the 2009 to 2021 GCRO QoL survey datasets from the GCRO repository (https://www.data first.etc.ac.za/data portal/index.php/collections/GCRO), authorisation was secured by submitting a written request to the GCRO. The study was carried out in accordance with the Declaration of Helsinki and the applicable ethical standards and laws. The Declaration of Helsinki and all applicable ethical rules and regulations were followed in the conduct of the study. The University of the Witwatersrand, Johannesburg GCRO QoL surveys' Ethics Committee (Project identification code:

Round 1 GCRO QoL 2009; Round 2 GCRO QoL 2011; Round 3 GCRO QoL 2013–2014; Round 4 GCRO QoL 2015–2016; Round 5 GCRO QoL 2017–2018; Round 6 GCRO QoL 2020–2021, and Round 6 GCRO QoL 2021–2024) approved the protocol. Only the use of the data for this research topic and the publication of the results in a peer-reviewed journal were conditions of the permission granted.

Institutional Review Board Statement: The GCRO obtains ethical clearance from the Human Research Ethics Committee (non-medical) from the University of the Witwatersrand, Johannesburg Research Office. Verbal consent was obtained from the respondents aged 18+. All respondents were informed about the voluntary nature of participation, including confidentiality and anonymity.

Informed Consent Statement: All human subjects gave their informed consent for inclusion before they participated in the study. We obtained approval to use the 2009 to 2024 GCRO data from the GCRO repository (https://www.datafirst.uct.ac.za/dataportal/index.php/catalog/GCRO/?page=1& sort_by=title&sort_order=asc&ps=15&repo=GCRO). The questionnaires, data, and documentation for our most recent survey (QoL 7 Survey 2023/24) as well as previous surveys (2009, 2011, 2013/14, 2015/16, 2017/18 & 2020/21) are available for download through DataFirst: https://www.datafirst.uct.ac.za/dataportal/index.php/catalog/GCRO. The study was conducted in accordance with the Declaration of Helsinki, as well as with the relevant ethical guidelines and regulations. The protocol was approved by the University of the Witwatersrand Ethics Committee for the GCRO Programme in partnership with the University of Johannesburg, the Gauteng Provincial Government (GPG), and organized Local government in Gauteng (SALGA-Gauteng) (Project identification code: 2009, 2011, 2013/14, 2015/16, 2017/18 & 2020/21 QoL surveys).

Data Availability Statement: Data are from the Gauteng City-Region Observatory (GCRO) and the dataset is open to qualified researchers free of charge. In order to access the data from the GCRO, a written request was submitted to the GCRO and permission was granted to use the data for this survey. To request access to the dataset, please apply at https://www.datafirst.uct.ac.za/dataportal/index.php/catalog/GCRO/?page=1&sort_by=title&sort_order=asc&ps=15&repo=GCRO.

Supplementary Information for Data and Materials: All data generated or analysed during this study are included in this published article [and its supplementary information files].

Acknowledgments
The authors extend their sincere gratitude to the Gauteng City-Region Observatory (GCRO) for granting access to the survey dataset, which was instrumental in conducting this research. Monica Ewomazino Akokuwebe acknowledges her affiliation with the Department of Science and Innovation and the National Research Foundation Centre of Excellence in Human Development at the University of the Witwatersrand, Johannesburg, South Africa. The views expressed in this study are solely those of the authors and do not necessarily reflect the perspectives of the GCRO or any affiliated institutions. Finally, we would like to thank Mrs. Helen Thomas for her meticulous support in language editing, which significantly enhanced the clarity and precision of this work.

Competing Interests: The authors declare no competing interests.

Consent for Publication: Not applicable.

Abbreviations

QoL	Quality of Life
GCRO QoLS	Gauteng City-Region Observatory's Quality of Life Survey
RSA	Republic of South Africa
SALGA	South African Local Government Association
GCR	Gauteng City Region
SWLS	Satisfaction with Life Scale
gSEM	generalized Structural Equation Modelling
HH	household
SASSA	South African Social Security Agency
GCRO	the Gauteng City Region Observatory

References

1. Akokuwebe, M.E., Likoko, S., Osuafor, G.N., & Idemudia ES. (2023a). Determinants of life satisfaction among migrants in South Africa: an analysis of the GCRO's quality of life survey (2009–2021). *BMC Public Health* 23, 2030 (2023). https://doi.org/10.1186/s12889-023-16868-1
2. Akokuwebe, M. E., Osuafor, G. N., Likoko, S., & Idemudia, E. S. (2023b). Health services satisfaction and medical exclusion among migrant youths in Gauteng Province of South Africa: A cross-sectional analysis of the GCRO survey (2017−2018). *PLOS ONE*, *18*(11), e0293958. https://doi.org/10.1371/journal.pone. 0293958
3. Ebrahim, A., Botha, F., & Snowball, J. (2013) Determinants of life satisfaction among race groups in South Africa, Development Southern Africa, 30:2, 168-185. https://doi.org/10.1080/0376835X.2013.797227
4. Nyagweta, D. T. (2024). Investigating the Immigrant Paradox in South Africa: Exploring Reported Health, Depression, and Life Satisfaction. *Forum for Social Economics*, 1–31. https://doi.org/10.1080/0736 0932.2024.2312138
5. López-Ortega, M., Torres-Castro, S. & Rosas-Carrasco, O. (2016). Psychometric properties of the Satisfaction with Life Scale (SWLS): secondary analysis of the Mexican Health and Aging Study. *Health Qual Life Outcomes* 14, 170. https://doi.org/10.1186/s12955-016-0573-9
6. Margolis, S., Schwitzgebel, E., Ozer, D. J., & Lyubomirsky, S. (2019). A New Measure of Life Satisfaction: The Riverside Life Satisfaction Scale. *Journal of Personality Assessment*, *101*(6), 621–630. https://doi.org/ 10.1080/00 223891.2018.1464457
7. Møller, V. (2007). Satisfied and dissatisfied South Africans: results from the General Household Survey in international comparison. *Soc Indic Res* 81, 389–415. https://doi.org/10.1007/s11205-006-9004-3
8. Posel, D. R., & Casale, D. M. (2011). Relative Standing and Subjective Well-Being in South Africa: The Role of Perceptions, Expectations and Income Mobility. *Social Indicators Research*, *104*(2), 195–223. http://www.jstor.org/stable/41476598
9. Meyer, D. F. & Dunga, S. H. (2014). The Determinants of Life Satisfaction in a Low-Income, Poor Community in South Africa. Mediterranean Journal of Social Sciences, 5(13): 163-171. Doi:10.5901/mjss.2014.v5n13 p163
10. Pana, Y., Sunny Chanb, H. W., Xua, Y., & Yeung, K. C. (2019). Determinants of life satisfaction and self-perception of ageing among elderly people in China: An exploratory study in comparison between physical and social functioning. *Archives of Gerontology and Geriatrics 84, 103910.*
11. Statista 2024. Socioeconomic Indicators – South Africa. Accessed on the 16th of April, 2024 from https:// www.statista.com/outlook/co/socioeconomic-indicators/south-africa.
12. Shen, X.; Yin, F.; & Jiao, C. (2023). Predictive Models of Life Satisfaction in Older People: A Machine Learning Approach. Int. J. Environ. Res. Public Health, 20, 2445. https://doi.org/10.3390/ijerph200324 45
13. Osuafor GN, Akokuwebe ME, & Idemudia ES (2024). Family planning decision making among young males in Southern Africa. Africa Journal of Reproductive Health, 27(10): 16-35.
14. Owusu, A. K., Dey, N. E. Y., Adade, A. E., & Agbadi, P. (2022). Determinants of life satisfaction among Ghanaians aged 15 to 49 years: a further analysis of the 2017/2018 multiple cluster Indicator Survey. PLoS ONE, 17 (1), e0261164.

15. Akokuwebe ME, Idemudia ES & Ugwu ES (2023). Universal health coverage and the 2030 SDG Targets: Interventions to prevent and combat drug/substance use, abuse and addiction amidst the Covid-19 pandemic. Afr. J. Biomed. Res., 26(3): 305-317.
16. Ngoo, Y. T., Tan, E. C., & Tey, N. P. (2020). Determinants of life satisfaction in Asia: a Quantile Regression Approach. J Happiness Stud.; 22(2): 907–926.
17. Akokuwebe ME, Idemudia ES, & Okuneye FC (2024). Excessive alcohol intake and health outcomes nexus among youths in Mushin area of Lagos State, Southwest Nigeria. Afr. J. Biomed. Res., 27(1): 29-38.
18. Kollamparambil, U. (2020). Happiness, Happiness Inequality and Income Dynamics in South Africa. *J Happiness Stud* 21, 201–222 (2020). https://doi.org/10.1007/s10902-019-00075-0
19. Botha, F. & Booysen, F. (2013). The relationship between marital status and life satisfaction among South African adults. Acta Academica; 45 (2):150–78.
20. Akokuwebe ME, & Idemudia ES (2023). Gender differences in positive sexual behaviour among a young adult cohort in South Africa: A cross-sectional study. African Journal of Reproductive Health, 27(7): 43-55.
21. Wijayatunga, S. L. (2021). Life Satisfaction among Healthcare Workers: A Cross-Sectional Study in Private Hospitals in Ragama, Sri Lanka. The International Journal of Indian Psychology, 9 (3): 1966-1983. Doi: 10.25215/0903.185.
22. Akokuwebe ME, Osuafor GN, Idemudia ES (2024). Prevalence and association of HIV and tuberculosis status in older adults in South Africa: an urgent need to escalate the scientific and political attention to aging and health. Frontiers in Public Health, section, Aging and Public Health, 12(124555): 01-15.
23. Tian, H. & Chen, J. (2022) Comparing the impact of social support on the life satisfaction of widowed and non-widowed elders. *Front. Psychol.* 13:1060217. doi: 10.3389/fpsyg.2022.1060217.
24. Ajaero CK, Ebimgbo S, Ezeibe C, Ugwu C, Nzeadibe C, & Osabede N. (2023). Life Satisfaction in South Africa: The Influence of Inter-Provincial Migration Status. Psychol Stud (April–June 2023) 68(2):197–210. https://doi.org/10.1007/s12646-022-00697-0
25. Muhammad, T., Paul, R., Meher, T., Rashmi, R., Srivastava, S. (2022). Decomposition of caste differential in life satisfaction among older adults in India. *BMC Geriatr* 22, 832. https://doi.org/10.1186/s12877-022-03526-1
26. Likoko S, Akokuwebe ME, Osuafor GN, & Idemudia ES (2023). "Health outcomes of grandparents caring for double orphans in South Africa": what are the determinants? International Journal of Environmental Research and Public Health, 20(24): 7158.
27. Mafini, C., & Meyer, D. F. (2016). Satisfaction with Life amongst the Urban Poor: Empirical Results from South Africa. AUDOE, 12 (5): 33-50.
28. Gauteng City-Region Observatory (GCRO). (2009). GCRO Quality of Life Survey I (2009). Gauteng City-Region Observatory, October 2009. Accessed on January 14th 2023, from https://cdn.gcro.ac.za/media/documents/GCRO_2009_QoL_survey_Life_in_the_city-region.pdf.
29. Gauteng City-Region Observatory (GCRO), Life Survey II. (2011). GCRO Quality of (2011). Gauteng City-Region Observatory, October 2011. Accessed on January 14th, 2023, from https://gcro.ac.za/research/ project/detail/ quality-of-life-survey-ii-2011/.
30. Gauteng City-Region Observatory (GCRO). (2013). GCRO Quality of Life Survey III (2013). Gauteng City-Region Observatory, October 2013. Accessed on January 14th, 2023, from https://gcro.ac.za/research/ project/detail/ quality-of-life-survey-iii-2013/.
31. Gauteng City-Region Observatory (GCRO). (2015/16). GCRO Quality of Life Gauteng City-Region Observatory (GCRO), Survey IV. (2015). GCRO Quality of Life Survey IV (2015). Gauteng City-Region Observatory, October 2015. Accessed on January 14th, 2023, from https://gcro.ac.za/research/project/ detail/quality-of-life-survey-iv-2015/.
32. Gauteng City-Region Observatory (GCRO). (2017/18). GCRO Quality of Life Gauteng City-Region Observatory (GCRO), Survey V. (2017/2018). Gauteng City-Region Observatory, October 2018. Accessed on January 14th, 2023, from https://gcro.ac.za/research/project/detail/quality-of-life-survey-v-201718/.
33. Gauteng City-Region Observatory (GCRO). (2020/21). GCRO Quality of Life Survey VI (2020/21). Gauteng City-Region Observatory, October 2021. Accessed on January 14th, 2023, from https://gcro.ac.za/ research/project/detail/quality-life-survey-vi-202021/.
34. Gauteng City-Region Observatory (GCRO). (2023/24). GCRO Quality of Life Survey VI (2023/24). Gauteng City-Region Observatory, June 2024. Accessed on June 30th, 2024, from https://gcro.ac.za/research/ project/detail/quality-life-survey-vi-202324/.
35. Statistics South Africa (Stats SA) (2011). Census 2011: Migration Dynamics in South Africa. Statistics South Africa (Stat SA), 2015, Report No. 03-01-79, 215 p. Published by Statistics South Africa, Private Bag X44, Pretoria 0001. Accessed on the 10th of December 2022 from https://www.statssa.gov.za/publications/ Report-03-01-79/Report-03-01-792011.pdf.
36. Statistics South Africa (Stat SA) (2020). Statistics South Africa releases results of 2020 General Household Survey – 2020 mid-year population estimates. Statistics South Africa (Stat SA), 2020. Published by Statistics South Africa, Private Bag X44, Pretoria

0001. Accessed on the 10th of. December 2022 from https://www.gov.za/speeches/statistics-south-africa-release-results-2020-general-household-survey-2-dec%C2%A0-1-dec-2021.
37. Statistics South Africa (Stat SA) (2022a). Draft National Labour Migration Policy for South Africa. Statistics South Africa., 2022, 115 p. Accessed on the 18th March 2022 from https://www.labour.gov.za/Document Center/Publications/Public%20Employment%20 services/National%20Labour%20Migration%20Policy%2 02021%202.pdf
38. Statistics South Africa (Stat SA) (2023a). Census 2022 shows South Africa's population grew to 62 million. Accessed on May 5, 2024 from https://www.statssa.gov.za/?p=16711.
39. Statistics South Africa (Stat SA) (2023b). Stats in brief 2023 - Improving lives through lives through Data Ecosystems. Accessed on May 5, 2024 from https://www.statssa.gov.za/publications/StatsInBrief/Stats InBrief2023.pdf
40. Statistics South Africa (Stat SA) (2022b). Mid-year population estimates, 2022 (P0302). Statistical Release P0302. Department: Statistics South Africa, Republic of South Africa. Accessed on the 20th of May, 2024, from https://www.statssa.gov.za/publications /P0302/P03022022.pdf
41. Statistics South Africa. (2012). *Provinces at a glance* (03-01–43). Statistics South Africa. https://www.statssa.gov.za/census/census2011/ censusproducts/Provinces%20at%20a%20glance%2016%20Nov%202012%20corrected.pdf
42. Statistics South Africa. (2023). *Census 2022* (P0301.4). Statistics South Africa. https://census.statssa.gov.za/assets/documents/2022/P0 3014_Census_2022_Statistical_Release.pdf
43. Statistics South Africa (Stat SA) (2022c). Census 2022: Gauteng Province. Department: Statistics South Africa, Republic of South Africa. Accessed on the 20th of May, 2024, from https://census.stat.ssa .gov.za/#/province/1/2.
44. Mercandalli S, Losch B, Belebema MN, Bélières J-F, Bourgeois R, Dinbabo MF, Fréguin-Gresh S, Mensah C, and Nshimbi CC (2019). Rural migration in sub-Saharan Africa, editors. Patterns, drivers and relation to structural transformation. Rome, FAO and CIRAD, 2019.
45. Akanle O, Kayode D, and Abolade I. (2022). Sustainable development goals (SDGs) and remittances in Africa. Cogent Social Sciences. 2022; 8(1): 2037811.
46. Republic of South Africa (1995). No. 88 of 1995: South African Citizenship Act, 1995. Government Gazette, 6 October 1995. Registered at the Post office as a Newspaper, Cape Town, 6 October 1995; vol. 364 (No. 16751): Pp. 1-26.
47. Lagani, V., Karozou, A.D., Gomez-Cabrero, D. et al. (2016). A comparative evaluation of data-merging and meta-analysis methods for reconstructing gene-gene interactions. BMC Bioinformatics 17 (Suppl 5), S194. https://doi.or g/10.1186/s12859-016-1038-1.
48. Lin D, Tantithamthavorn C, Hassan AE (2021). The impact of data merging on the interpretation of cross-project just-in-time defect models. IEEE Transactions on Software Engineering, 48 (8): 2969–2986.
49. Zhao, E. Y., Xia, D., Greenhalgh, M., Colicino, E., Monaro, M., Hitching, R., Harris, O. A., & Adamson, M. M. (2021). Combining International Survey Datasets to Identify Indicators of Stress during the COVID-19 Pandemic: A Machine Learning Approach to Improve Generalization. *COVID*, *1*(4), 728-738. https://doi.org/10.3390/covid10400 58.
50. Muehlenhard, C.L., Peterson, Z.D. (2011). Distinguishing Between *Sex* and *Gender*: History, Current Conceptualizations, and Implications. *Sex Roles* 64, 791–803 (2011). https://doi.org/10.1007/s11199-011-9932-5
51. McDonald, B., & Kanske, P. (2023). Gender differences in empathy, compassion, and prosocial donations, but not theory of mind in a naturalistic social task. *Scientific Reports*, *13*(1), 1-15. https://doi.org/10.1038/s41598-023-47747-9
52. Diener E, Emmons RA, Larsen RJ, and Griffin S. (1995). The satisfaction with Life Scale. J Pers Assess. 1985; 49 (1): 71–75.
53. Diener E, Pressman SD, Hunter J, and Delgadillo-Chase D. (2017). If, why, and when Subjective Well-Being Influences Health, and future needed research. Volume 9. Applied Psychology: Health and Well-Being; 2017. pp. 133–67. 2.
54. Deaton A. (2008). Income. Health, and well-being around the World: evidence from the Gallup World Poll. J Economic Perspect.; 22(2): 53–72.
55. Cantril H (1965). The pattern of human concerns. New Brunswick, NJ: Rutgers University Press; 1965.
56. Gallup (2009). World Poll Methodology. Technical Report, 2009. Washington, DC.
57. Milovanska-Farrington S, & Farrington, S. (2022). Happiness, domains of life satisfaction, perceptions, and valuation differences across genders. Acta Psychol.; 230:103720.
58. Ginsburg C, Collinson MA, Gómez-Olivé FX, Gross M, Harawa1 S, Lurie MN, Mukondwa K, Pheiffer CF, Tollman S, Wang R, & White MJ (2021). Internal migration and health in South Africa: determinants of healthcare utilisation in a young adult cohort. BMC Public Health; 21:554.
59. Prati A, & Senik C. (2022). Feeling good is feeling better. Psychol Sci.; 33 (11):1828–1841.
60. Joshanloo M. (2018). Gender differences in the predictors of life satisfaction across 150 nations. Pers Indiv Differ, 135.

61. Fan, Y., Chen, J., Shirkey, G., John, R., Wu, S. R., Park, H., & Shao, C. (2016). Applications of structural equation modeling (SEM) in ecological studies: An updated review. *Ecological Processes*, *5*(1), 1-12.
62. Oyedokun, A.O., Jegede, P.O., Onyeka, B.C. (2023). Structural Equation Modeling with Stata: Illustration Using a Population-Based, Nationally-Representative Dataset. In: Awe, O.O., Vance, E.A. (eds) Sustainable Statistical and Data Science Methods and Practices. STEAM-H: Science, Technology, Engineering, Agriculture, Mathematics & Health. Springer, Cham. https://doi.org/10.1007/978-3-031-41352-0_16
63. Lombardi, S., Santini, G., Marchetti, G. M., & Focardi, S. (2017). Generalized structural equations improve sexual-selection analyses. *PLoS ONE*, *12*(8).
64. Gómez-Balcácer, L., Somarriba Arechavala, N. & Gómez-Costilla, P. (2023). The Importance of Different Forms of Social Capital for Happiness in Europe: A Multilevel Structural Equation Model (GSEM). *Applied Research Quality Life* **18**, 601–624.
65. Cain, M. K. (2020). Structural equation modeling using Stata. Journal of Behavioral Data Science, 1(2),156–177.
66. Audette, A.P., Lam, S., O'Connor, H. *et al.* (2019). (E)Quality of Life: A Cross-National Analysis of the Effect of Gender Equality on Life Satisfaction. *J Happiness Stud* **20**, 2173–2188.
67. Meisenberg, G., Woodley, M.A. (2015). Gender Differences in Subjective Well-Being and Their Relationships with Gender Equality. *J Happiness Stud* **16**, 1539–1555.
68. Becchetti, L., Conzo, G. (2022). The Gender Life Satisfaction/Depression Paradox. *Soc Indic Res* **160**, 35–113.
69. Joshanloo, M., Jovanović, V. (2020). The relationship between gender and life satisfaction: analysis across demographic groups and global regions. *Arch Womens Ment Health* **23**, 331–338 (2020).
70. Graham C (2020). Are women happier than men? Do gender rights make a difference? This piece is part of 19A: The Brookings Gender Equality Series. Accessed on October 11th, from https://www.brookings.edu/articles/ar e-women-happier-than-men-do-gender-rights-make-a-difference/
71. Black, J., Mendenhall, M. (1991). The U-Curve Adjustment Hypothesis Revisited: A Review and Theoretical Framework. *J Int Bus Stud* **22**, 225–247 (1991).
72. Moran, T. P., Bengtsson, E., Missiaia, A., Olsson, M., Svensson, P., Kwon, R., Wisman, J. D., Pacitti, A., Wisman, J. D., Wisman, J. D., Davis, M. E., Tausch, A., Heshmati, A., Tausch, A., Korzeniewicz, R. P., Moran, T. P., & Gullette, G. S. (2005). Kuznets's Inverted U-Curve Hypothesis: The Rise, Demise, and Continued Relevance of a Socioeconomic Law. *Sociological Forum*, *20*(2), 209-244.
73. Moran, T. P. (2005). Kuznets's Inverted U-Curve Hypothesis: The Rise, Demise, and Continued Relevance of a Socioeconomic Law. *Sociological Forum*, *20*(2), 209-244.
74. Schafer MH, Mustillo SA, Ferraro KF (2013). Age and the Tenses of Life Satisfaction. *The Journals of Gerontology: Series B*, 68 (4), 571–579.
75. Huber, BR. (2014). Does life satisfaction increase with age? Only in some places, new study finds. Woodrow Wilson School of Public and International Affairs. Accessed on the October 11th, 2024 from https://www.princeton.edu/news/2014/11/06/does-life-satisf action-increase-age-only-some-places-new-study-finds.
76. Karwetzky, C., Michaelsen, M. M., Werdecker, L., & Esch, T. (2022). The U-Curve of Happiness Revisited: Correlations and Differences in Life Satisfaction Over the Span of Life—An Empirical Evaluation Based on Data From 1,597 Individuals Aged 12–94 in Germany. *Frontiers in Psychology*, *13*, 837638.
77. Handa, S., Pereira, A. & Holmqvist, G. (2023). The Rapid Decline of Happiness: Exploring Life Satisfaction among Young People across the World. *Applied Research Quality Life* **18**, 1549–1579.
78. Huber, BR. (2014). Does life satisfaction increase with age? Only in some places, new study finds. Woodrow Wilson School of Public and International Affairs. Accessed on the October 11th, 2024 from https://www.princeton.edu/news/2014/11/06/does-life-satisfaction-increase-age-only-some-places-new-study-finds.
79. Gondek, D., Bernardi, L., McElroy, E. *et al.* (2024). Why do Middle-Aged Adults Report Worse Mental Health and Wellbeing than Younger Adults? An Exploratory Network Analysis of the Swiss Household Panel Data. *Applied Research Quality Life* **19**, 1459–1500 (2024).
80. Li, A., Wang, D., Lin, S., Chu, M., Huang, S., Lee, C., & Chiang, Y. (2021). Depression and Life Satisfaction among Middle-Aged and Older Adults: Mediation Effect of Functional Disability. *Frontiers in Psychology*, *12*, 755220.
81. Park, J., & Kang, S. (2022). Factors Related to Life Satisfaction of Older Adults at Home: A Focus on Residential Conditions. *Healthcare*, *10*(7), 1279.
82. da Silva, F.R., De Paula, T.M., Gerhard, F. *et al.* Does tackling the sustainable development goals lead to life satisfaction?. *Environ Dev Sustain* (2024). https://doi.org/10.1007/s10668-024-04640-3

83. Wang, W., Li, J., Sun, G., Cheng, Z., & Zhang, X. (2017). Achievement goals and life satisfaction: The mediating role of perception of successful agency and the moderating role of emotion reappraisal. *Psicologia: Reflexão e CríTica*, *30*(1), 1-12.
84. Booysens F, Botha F. (2013). Family functioning and life satisfaction and happiness in South African households. Economic Research Southern Africa (Ersa), Working Paper. Accessed on the 11th, of October, 2024 from https://econrsa.org/publications/family-functioning-and-life-satisfaction-and-happiness-in-south-african-household/
85. Botha, F., Booysen, F. & Wouters, E. (2018). Satisfaction with Family Life in South Africa: The Role of Socioeconomic Status. *J Happiness Stud* **19**, 2339–2372.
86. Malvaso, A., & Kang, W. (2022). The relationship between areas of life satisfaction, personality, and overall life satisfaction: An integrated account. *Frontiers in Psychology*, *13*. https://doi.org/10.3389/f psyg.2022.894610
87. Chowhan, J., Samavatyan, H. & HakemZadeh, F. (2024). Life Satisfaction and the Roles of Work, Family, and Social Factors in a Social Production Function Framework. *J Happiness Stud* **25**, 18.
88. Lin, Y., Xiao, H., Lan, X. *et al.* (2020). Living arrangements and life satisfaction: mediation by social support and meaning in life. *BMC Geriatr.* **20**, 136.
89. Acolin, A., Reina, V. (2022). Housing cost burden and life satisfaction. *J Hous and the Built Environ* **37**, 1789–1815. https://doi.org/10.1007/s10901-021-09921-1
90. Botha, F., Booysen, F. (2014). Family Functioning and Life Satisfaction and Happiness in South African Households. *Soc Indic Res* 119, 163–182.
91. Au, A., Lai, D. W., Yip, H., Chan, S., Lai, S., Chaudhury, H., Scharlach, A., & Leeson, G. (2020). Sense of Community Mediating Between Age-Friendly Characteristics and Life Satisfaction of Community-Dwelling Older Adults. *Frontiers in Psychology*, *11*, 511384.
92. Dayimani A, Musvoto G. (2023). Chapter 8: Built environment strategies for reducing crime in informal settlements: A case study. In: The dynamics of residential satisfaction in low-income human settlements in South Africa. Edited by Godfrey G. Musvoto. Unisa Press, pp 147. ISBN 978-1-77615-190-5 and 978-1-77615-191-2.
93. Musvoto, G.G. (2023). Chapter 1: Residential satisfaction in South Africa's low-income urban neighbourhoods: an introduction. In: The dynamics of residential satisfaction in low-income human settlements in South Africa. Edited by Godfrey G. Musvoto. Unisa Press, pp 395. ISBN 978-1-77615-190-5 and 978-1-77615-191-2.
94. Botha, F., Snowball, J., de Klerk, V., Radloff, S. (2015). Determinants of Student Satisfaction with Campus Residence Life at a South African University. In: Maggino, F. (eds) A New Research Agenda for Improvements in Quality of Life. Social Indicators Research Series, vol 57. Springer, Cham. https://doi.org/10.1007/978-3-319-15904-1_2
95. Ramklass, S., Anderson, S., Keating, N., & Cassim, B. (2015). Features of home and neighbourhood and the liveability of older South Africans. *European Journal of Ageing*, *12*(3), 215-227.
96. Jones, B. A., Bouman, W. P., Haycraft, E., & Arcelus, J. (2019). The Gender Congruence and Life Satisfaction Scale (GCLS): Development and validation of a scale to measure outcomes from transgender health services. *The International Journal of Transgenderism*, *20*(1), 63-80.
97. Chen, X., Cai, Z., He, J. *et al.* (2020). Gender Differences in Life Satisfaction among Children and Adolescents: A Meta-analysis. *J Happiness Stud* **21**, 2279–2307.
98. Vinod Kumar, T. K. (2020). The Influence of Demographic Factors and Work Environment on Job Satisfaction among Police Personnel: An Empirical Study. *International Criminal Justice Review*.
99. Sirgy, M.J. (2021). Effects of Demographic Factors on Wellbeing. In: The Psychology of Quality of Life. Social Indicators Research Series, vol 83. Springer, Cham.
100. Malvaso, A., & Kang, W. (2022). The relationship between areas of life satisfaction, personality, and overall life satisfaction: An integrated account. *Frontiers in Psychology*, *13*, 894610.
101. Itani, A., Hasan, I., Younes, L., & Alzaatreh, A. (2024). Statistical analysis of the effect of socio-political factors on individual life satisfaction. Scientific Reports, 14(1), 1-16.
102. Fernández-Ballesteros, R., Dolores Zamarrón, M., & Angel Ruíz, M. (2001). The contribution of socio-demographic and psychosocial factors to life satisfaction. *Ageing and Society*, *21*(1), 25–43.
103. Botha, F. (2014). Life Satisfaction and Education in South Africa: Investigating the Role of Attainment and the Likelihood of Education as a Positional Good. *Soc Indic Res* 118, 555–578.
104. Cheung, F., & Lucas, R. E. (2015). When Does Money Matter Most? Examining the Association between Income and Life Satisfaction over the Life Course. *Psychology and Aging*, *30*(1), 120.
105. Salland, J. (2018). Income comparison, gender roles and life satisfaction. *Applied Economics Letters*, *25*(20), 1436–1439.

106. Becchetti, L., Conzo, G. (2022). The Gender Life Satisfaction/Depression Paradox. *Soc Indic Res* 160, 35–113 (2022).
107. FitzRoy, F.R., Nolan, M.A. (2022). Income Status and Life Satisfaction. *J Happiness Stud* 23, 233–256 (2022).
108. Gschwandtner, A., Jewell, S. & Kambhampati, U.S. (2022). Lifestyle and Life Satisfaction: The Role of Delayed Gratification. *J Happiness Stud* 23, 1043–1072 (2022). https://doi.org/10.1007/s10902-021-00440-y
109. Montgomery, M. (2022). Reversing the gender gap in happiness. *Journal of Economic Behavior & Organization*, *196*, 65-78.
110. He, X., Huang, X. & Huang, C. (2024). Social participation and life satisfaction among middle-aged and elderly people in China: the mediating role of depression and the moderating role of relative income. *Curr Psychol* 43, 27521–27533 (2024).
111. Akokuwebe ME, Idemudia ES. (2023). Factors associated with choice of home birth as place of delivery among women of reproductive age in South Africa. *Afr J Reprod Health; 27 [1]: 22-40).*
112. Paul R., Pai M., Thalil M., & Srivastava S. (2024). Differences in Life Satisfaction among Older Adults in India. In: Helliwell, J. F., Layard, R., Sachs, J. D., De Neve, J.-E., Aknin, L. B., & Wang, S. (Eds.). (2024). World Happiness Report 2024. University of Oxford: Wellbeing Research Centre. http://doi.org/10.18724/whr-2f21-he52.
113. Masuda, Y. J., Williams, J. R., & Tallis, H. (2021). Does Life Satisfaction Vary with Time and Income? Investigating the Relationship among Free Time, Income, and Life Satisfaction. *Journal of Happiness Studies*, *22*, 2051.
114. Akokuwebe ME, Idemudia ES. (2023). Prevalence and Knowledge of Kidney Disease Risk Factors among Nigerians resident in Lagos State Metropolitan District, South West, Nigeria. *Ann Afr Med*.; 22 (1): 18-32.
115. Stirman, S. W., & Beidas, R. S. (2020). Expanding the Reach of Psychological Science Through Implementation Science: Introduction to the Special Issue. *The American Psychologist*, *75*(8), 1033.
116. Akokuwebe, M. E., & Oluwawole, A. O. (2016). Factors influencing acceptability of family planning among women in rural communities in Ife central local government area, Osun State. Ibadan J. Soc. Sci., 14, 250–261.
117. Oertelt-Prigione S., Dalibert L., Verdonk P., Zemp Stutz E., Klinge I (2017). Implementation Strategies for Gender-Sensitive Public Health Practice: A European Workshop. Journal of Women's Health, 26 (11), 1255-1261.
118. Akokuwebe ME, Idemudia ES. (2022). Knowledge and Risk Perceptions of Chronic Kidney Disease among Women of Childbearing Age in Lagos State, Nigeria: From a Health Demography Approach. *Int J Nephrol;* 5511555, 1-16.
119. Bauer, M. S., & Kirchner, J. (2020). Implementation science: What is it and why should I care?. *Psychiatry research*, *283*, 112376.
120. Holtrop, J. S., Scherer, L. D., Matlock, D. D., Glasgow, R. E., & Green, L. A. (2021). The Importance of Mental Models in Implementation Science. *Frontiers in Public Health*, *9*.
121. Stewart, R., Wright, B., Smith, L., Roberts, S., & Russell, N. (2021). Gendered stereotypes and norms: A systematic review of interventions designed to shift attitudes and behaviour. *Heliyon*, *7*(4).
122. Tannenbaum, C., Greaves, L., & Graham, I. D. (2016). Why sex and gender matter in implementation research. *BMC Medical Research Methodology*, *16*.

Disclaimer/Publisher's Note: The statements, opinions and data contained in all publications are solely those of the individual author(s) and contributor(s) and not of MDPI and/or the editor(s). MDPI and/or the editor(s) disclaim responsibility for any injury to people or property resulting from any ideas, methods, instructions or products referred to in the content.

Figure 1

Click here to access/download;Figure;Figure 1.tif

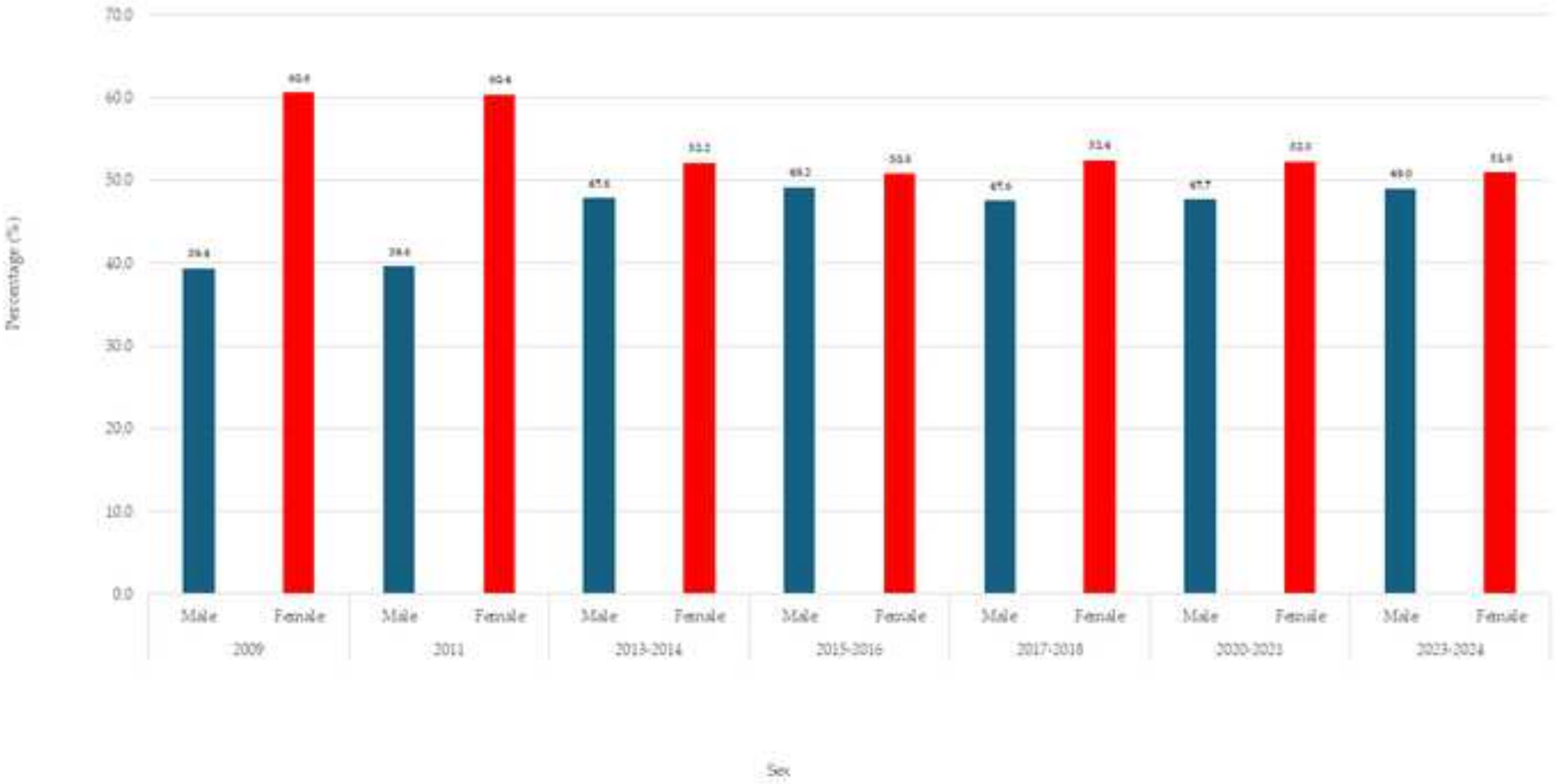

Figure 2

Click here to access/download;Figure;Figure 2.tif

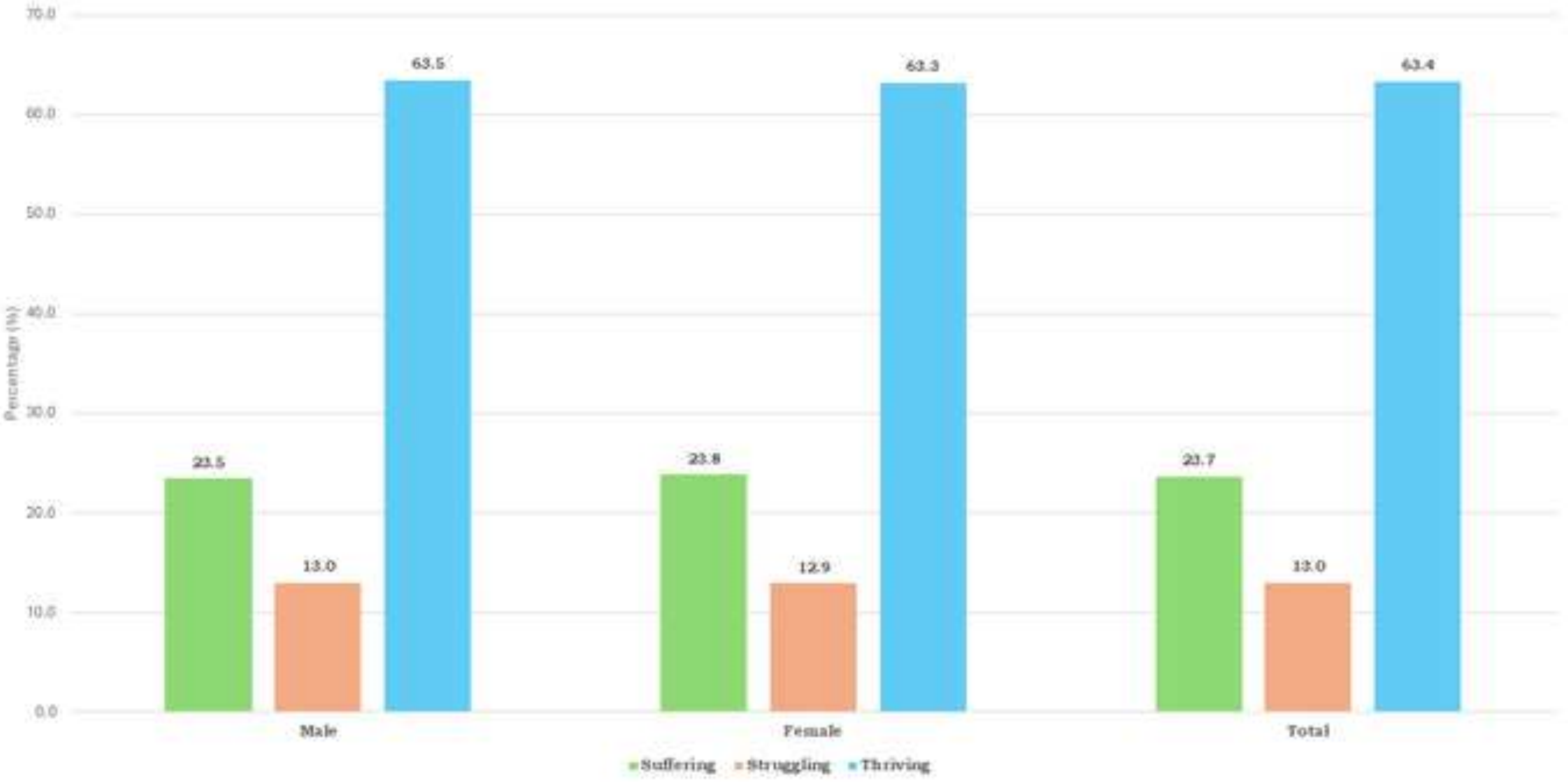

Figure 3

Click here to access/download;Figure;Figure 3.tif

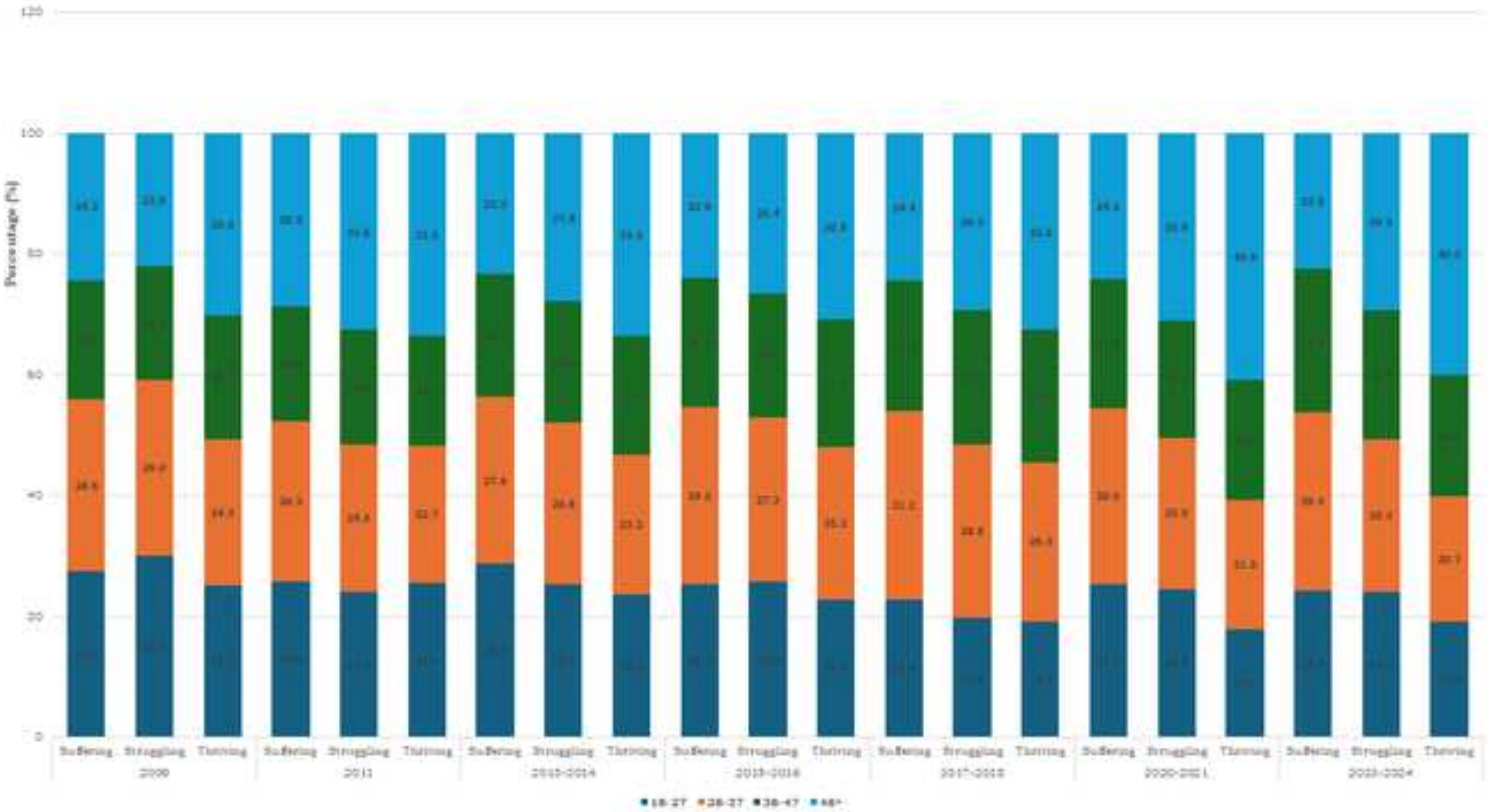

Figure 4

Click here to access/download;Figure;Figure 4.tif

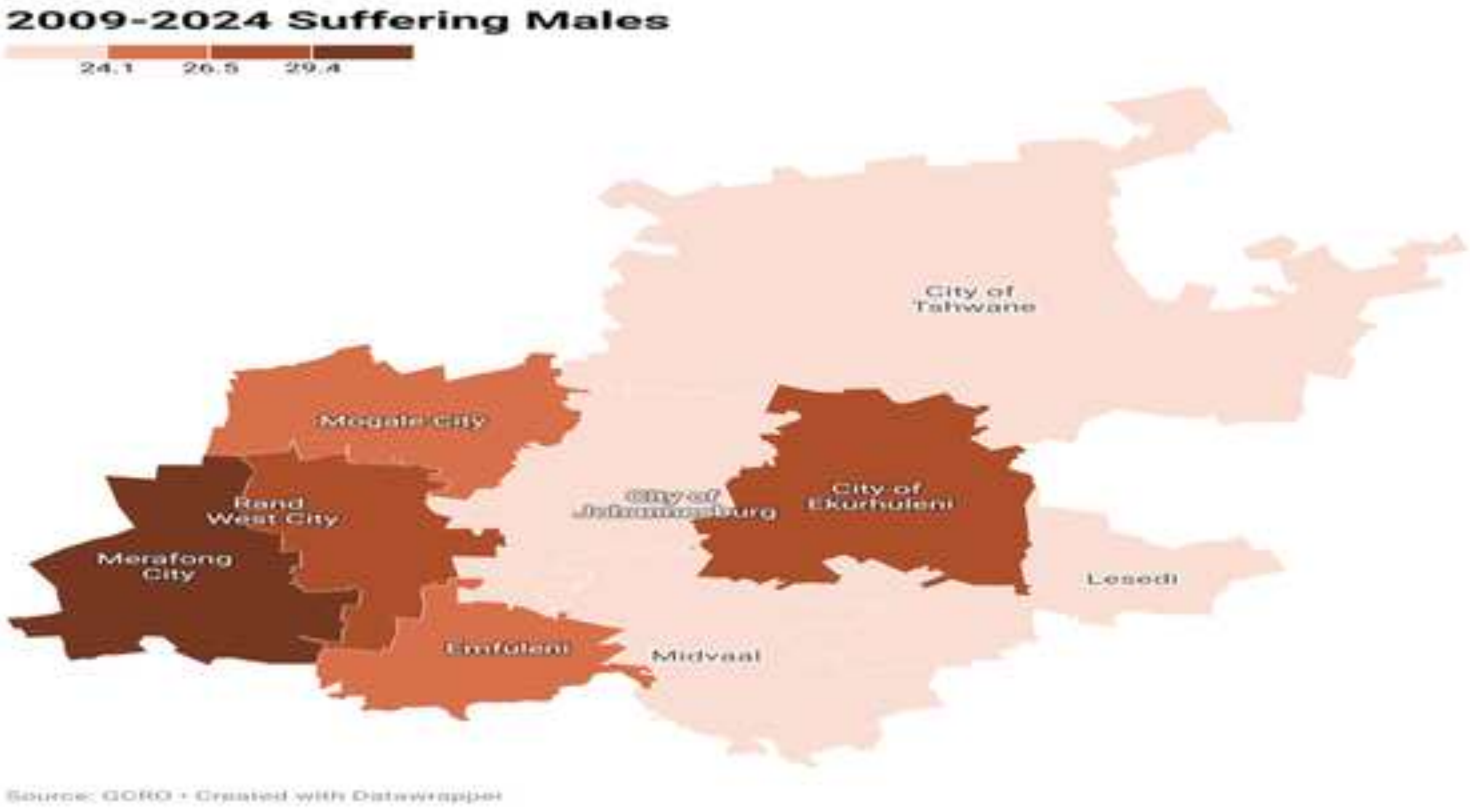

Figure 5

Click here to access/download;Figure;Figure 5.tif

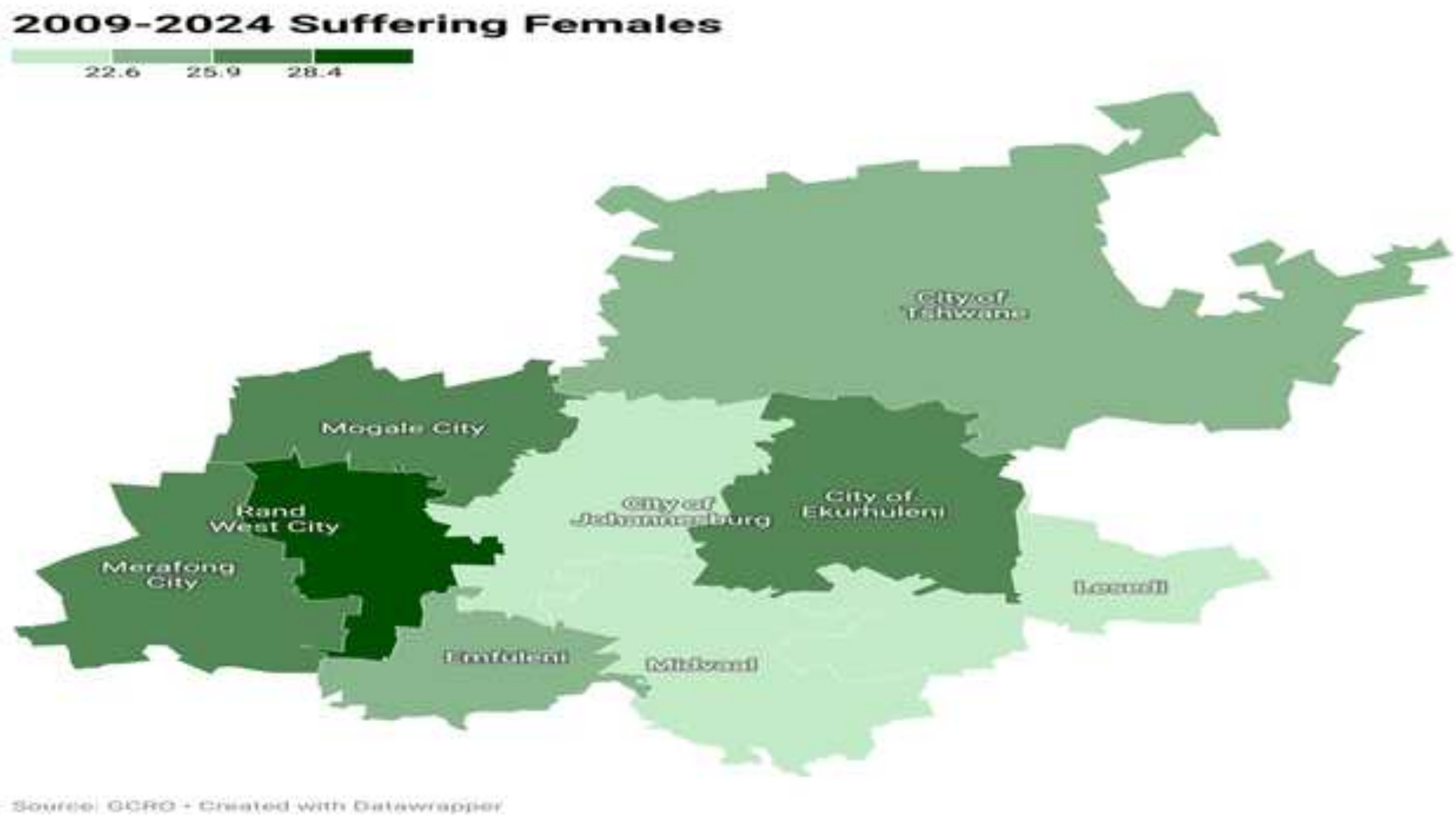

Figure 6

Click here to access/download;Figure;Figure 6.tif

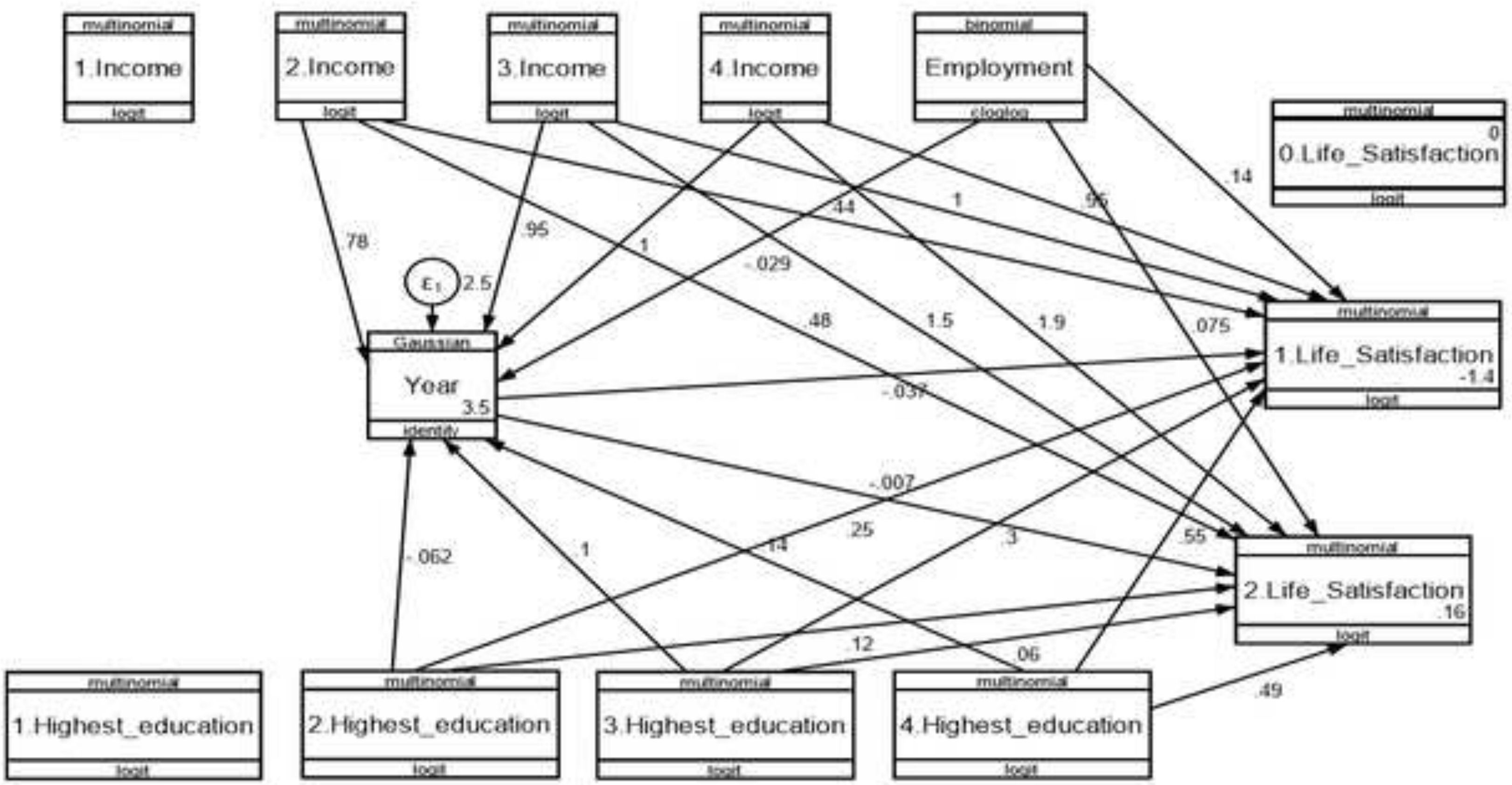

Figure 7

Click here to access/download;Figure;Figure 7.tif

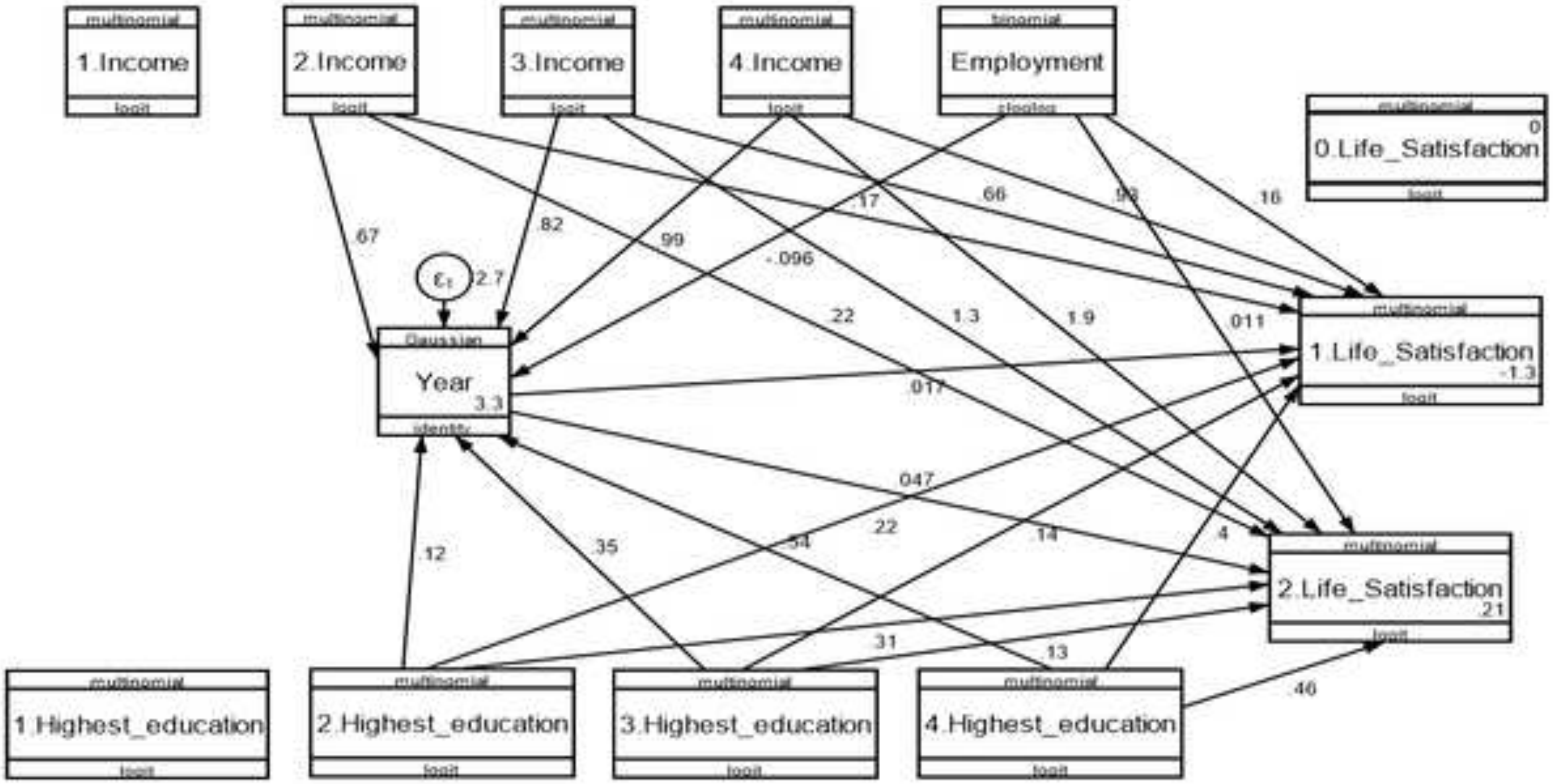

Click here to access/download
Supporting Information
Figure 1-7 with Legends.docx

Click here to access/download
Supporting Information - Compressed/ZIP File Archive
Do files as stata file.zip
