# Supplementary material for: “Gender dimensions of quality of life”: The determinants of life satisfaction among South Africans residing in the Gauteng province—Using a multilevel psychodemographic analysis of the GCRO’s quality of life survey (2009–2024)
Source: PLoS One. 2025 Sep 15;20(9):e0332626. doi: 10.1371/journal.pone.0332626 (PMC12435731; doi:10.1371/journal.pone.0332626)
Supplement: Attachment — Submitted filename: Recommendations.docx [file pone.0332626.s002.docx]

Recommendations:

1. Condense Abstract and Introduction. Highlight novelty in work and policy implications.
2. Include more interpretation in Results.
3. Discussion can be included with specific policy actions.
