# Supplementary material for: “Gender dimensions of quality of life”: The determinants of life satisfaction among South Africans residing in the Gauteng province—Using a multilevel psychodemographic analysis of the GCRO’s quality of life survey (2009–2024)
Source: PLoS One. 2025 Sep 15;20(9):e0332626. doi: 10.1371/journal.pone.0332626 (PMC12435731; doi:10.1371/journal.pone.0332626)
Supplement: Attachment — Submitted filename: Response to Editor Comment.docx [file pone.0332626.s005.docx]

**Rebuttal letter**

**Response to Academic Editor**

**Academic Editor**: We've checked your submission and before we can proceed, we need you to address the following issues:

1. All relevant data underlying the findings of this study will be made available in a public repository upon acceptance of the manuscript. De-identified data will be deposited in the Journal repository and a DOI link will be provided in the final published article. Any potentially identifying or sensitive participant information will be removed to ensure compliance with ethical approval and participant confidentiality. Further, data are from the Gauteng City-Region Observatory (GCRO) and the dataset is open to qualified researchers free of charge. In order to access the data from the GCRO, a written request was submitted to the GCRO and permission was granted to use the data for this survey. To request access to the dataset, please apply at <https://www.datafirst.uct.ac.za/dataportal/index.php/catalog/GCRO/?page=1&sort_by=title&sort_order=asc&ps=15&r> epo=GCRO

Supplementary Information for Data and Materials: All relevant data supporting the findings of this study are included in the published article and its supplementary information files. The do-files used to analyze the datasets are also provided within the supplementary information to ensure full reproducibility of the results.

**Authors’ Response:** The Authors have address the above following issues as follows:

**Data Availability Statement:**

All relevant data underlying the findings of this study will be made available in a public repository upon acceptance of the manuscript. De-identified data will be deposited in the Journal repository and a DOI link will be provided in the final published article. Any potentially identifying or sensitive participant information will be removed to ensure compliance with ethical approval and participant confidentiality. Further, data are from the Gauteng City-Region Observatory (GCRO) and the dataset is open to qualified researchers free of charge. In order to access the data from the GCRO, a written request was submitted to the GCRO and permission was granted to use the data for this survey. To request access to the dataset, please apply at https://[www.data](http://www.data)first.uct.ac.za/dataportal/index.php/catalog/GCRO/?page=1&sort_by=title&s ort_order=asc&ps=15&repo=GCRO

**Supplementary Information for Data and Materials:** All relevant data supporting the findings of this study are included in the published article and its supplementary information files. The do-files used to analyze the datasets are also provided within the supplementary information to ensure full reproducibility of the results.
